# Determining structure and Zn-specific Lewis acid-base descriptors for diorganozincs in non-coordinating solvents using X-ray spectroscopy

**DOI:** 10.1038/s42004-025-01704-x

**Published:** 2025-10-03

**Authors:** Lewis G. Parker, Frances K. Towers Tompkins, Jake M. Seymour, Najaat Alblewi, Ekaterina Gousseva, Megan R. Daw, Shusaku Hayama, Richard P. Matthews, Adam E. A. Fouda, Joshua D. Elliott, Christopher D. Smith, Kevin R. J. Lovelock

**Affiliations:** 1 Department of Chemistry, University of Reading, Reading, UK; 2 Diamond Light Source, Harwell Science and Innovation Campus, Didcot, UK; 3 Department of Biosciences, University of East London, London, UK; 4 Department of Physics, The University of Chicago, Chicago, IL USA; 5 Chemical Sciences and Engineering Division, Argonne National Laboratory, Lemont, IL USA

**Keywords:** Organometallic chemistry, Physical chemistry

## Abstract

Diorganozinc reagents (ZnR_2_, e.g. R = Et, Ph, C_6_F_5_) are widely used as Lewis acid catalysts or Lewis base reagents in their own right. However, descriptors for predicting the influence of the R substituent on ZnR_2_ Lewis acidity/basicity are very sparse. This is because ZnR_2_ liquid-phase speciation and electronic structure are unknown to date due to zinc’s ‘*spectroscopically quiet*’ nature and inability to measure ‘*at zinc*’. Here, we identify the geometric structures of ZnR_2_ in weakly coordinating solvents, demonstrating that electronic structure factors will dominate reactivity. We quantify the electronic structure properties that dictate ZnR_2_ Lewis acidity/basicity using three newly developed zinc-specific descriptors by combining the results from three zinc-specific X-ray spectroscopy methods and calculations. We provide accessible methods to pre-screen ZnR_2_ reactivity. Furthermore, our X-ray spectroscopy toolkit offers opportunities to develop liquid-phase descriptors that dictate reactivity for other zinc species, e.g. zinc bis-amides, battery electrolytes and enzymes.

## Abstract (web summary)

Diorganozinc reagents (ZnR_2_, e.g. R = Et, Ph, C_6_F_5_) are widely used as Lewis acid catalysts or Lewis base reagents, however, descriptors for predicting the influence of the R substituent are scarce. Here, by using liquid-phase X-ray spectroscopy, the authors have identified the geometric structures of diorganozincs in weakly coordinating solvents and then established Zn-specific descriptors to quantify the properties of their underlying Lewis acidity/basicity.

## Introduction

Diorganozincs (ZnR_2_)^1,2^ have a special place in chemical history; diethylzinc (ZnEt_2_) was amongst the first organometallics (Frankland C19^th^)^3^. ZnR_2_ can range in reactivity from Lewis basic/nucleophilic (*i.e*. electron donating) in e.g. transmetalation^4^ or nucleophilic substitution reactions^5–12^ through to Lewis acidic/electrophilic^9,11–20^ (*i.e*. electron acceptor). This diversity of reactivity has led to considerable liquid-phase applications in synthetic chemistry: the Nobel Prize-recognised Negishi cross-coupling reaction^21–25^; asymmetric autocatalysis of the Soai reaction^26^; Lewis acid catalysis^27^; frustrated Lewis pair catalysts (FLPs)^15^; insertion chemistries (CO_2_ and SO_2_)^28,29^; transfer reagents^30–33^; and in nanomaterial preparation^13,14^.

**Fig. 1:**
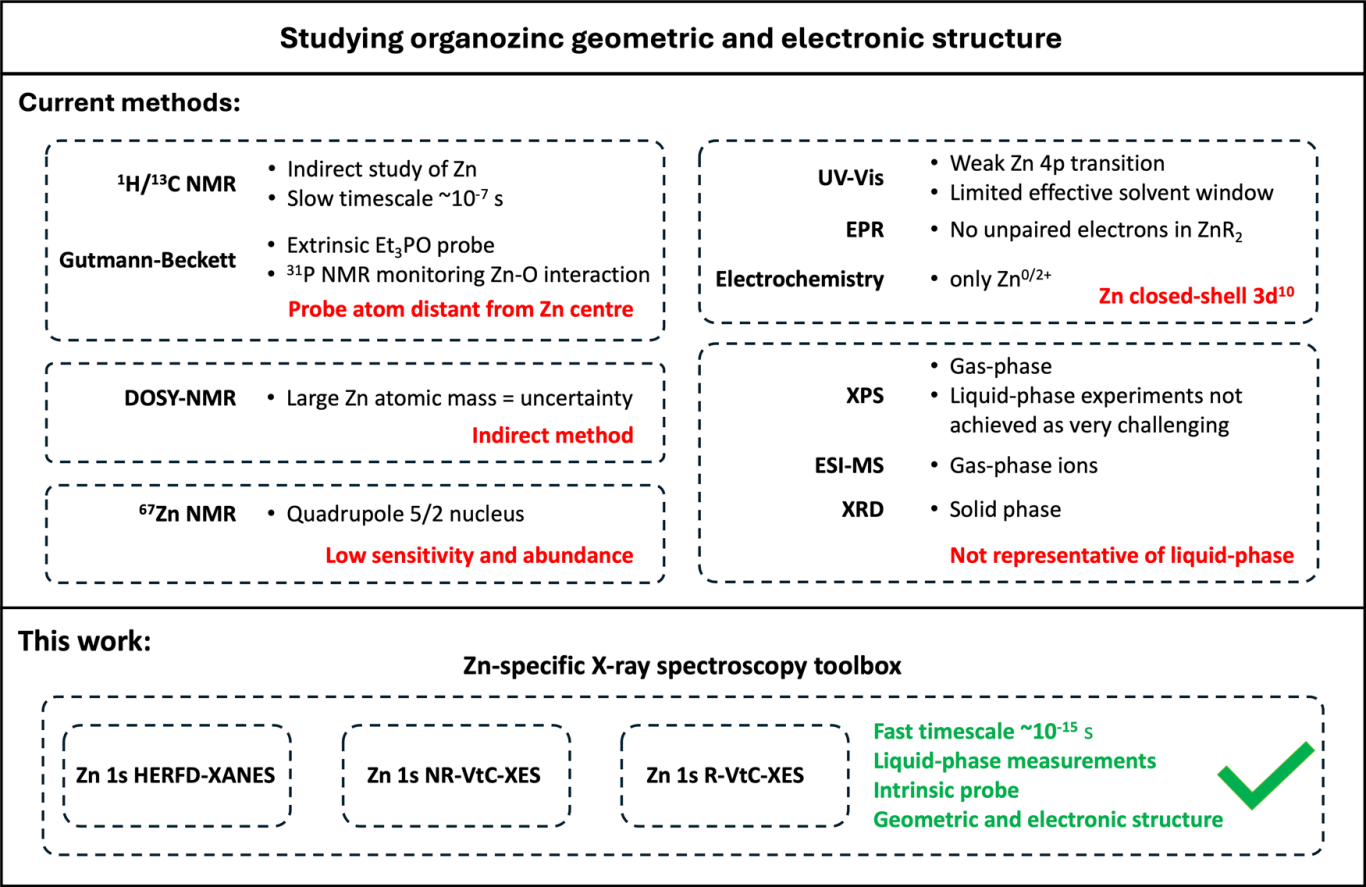
Current diorganozinc analysis methods, their limitations, and our liquid-phase X-ray spectroscopy approach. Overview of current methods for studying ZnR_2_ complexes and their shortcomings and our work here using an advanced X-ray spectroscopy toolkit for studying liquid-phase ZnR_2_ complexes.

These significant advances in ZnR_2_ chemistry have been driven mainly by empirical, iterative synthetic experimentation, as liquid-phase ZnR_2_ species are *spectroscopically quiet* (Fig. 1)^34,35^. In a telling approach, the enzymatic community at times have substituted Zn^2+^ for other metal cations (e.g. Co^2+^, Cu^2+^, Cd^2+^ etc.) that were easier to observe spectroscopically in model systems^34^. This spectroscopic near-silence has led to a dearth of descriptors for ZnR_2_, severely limiting the interpretation, prediction and fine-tuning of ZnR_2_ reactivity. Liquid-phase EPR and UV-Vis spectroscopies are ineffective for producing descriptors for closed-shell d^10^ Zn^2+^. One quantitative Lewis acidity descriptor, the electron acceptor number measured using ^31^P NMR spectroscopy, has been used for three ZnR_2_ compounds^11^. This method investigates the interaction of ZnR_2_ with triethylphosphine oxide. However, it is unsatisfactory because the phosphorus atom is positioned at a distance from the zinc centre^36^, which is the site of ZnR_2_ reactivity e.g. electron acceptance for Lewis acidic ZnR_2_.

Experimental or calculated electronic structure data is often used to produce Lewis basicity/acidity descriptors^37–40^. Advantageously, these intrinsic descriptors capture Lewis basicity/acidity independent of any probe molecule. However, liquid-phase experimental electronic structure spectroscopy has been severely limited by experimental challenges. Due to the vacuum conditions required, liquid-phase photoelectron spectroscopy studies are very challenging even for simple aqueous solutions^41^, let alone very reactive ZnR_2_ solutions. Gas-phase photoelectron spectroscopy has been used to produce a Lewis basicity descriptor, the highest occupied molecular orbital (HOMO) energy, for four ZnR_2_ (R = alkyl)^38^; however, this descriptor is not representative of liquid-phase ZnR_2_, leaving a huge unanswered question over solvent influence. Therefore, for liquid-phase ZnR_2_ the frontier orbitals (e.g. the HOMO and the lowest unoccupied molecular orbital, LUMO) have never been experimentally investigated let alone quantified.

In this article we use zinc-specific X-ray spectroscopies to produce zinc-specific, intrinsic descriptors, as hard X-ray spectroscopies overcome experimental challenges for liquid-phase studies as vacuum conditions are not required^42,43^. The zinc-specificity of these X-ray spectroscopy-derived descriptors give direct insight into the zinc centre, where ZnR_2_ reactivity occurs, e.g. electron donation for Lewis basic/nucleophilic ZnR_2_. Zn 1s X-ray absorption near edge structure (XANES) spectroscopy has been used widely for liquid-phase zinc compounds but sparingly for organozinc compounds^44–46^. Only two liquid-phase studies of Zn 1s non-resonant valence-to-core X-ray emission spectroscopy (NR-VtC-XES) are known, both for ZnCl_2_-based samples^47,48^, and none for Zn 1s resonant VtC-XES (R-VtC-XES), highlighting the novelty of our approach. For metals, the main usage of XANES, NR-VtC-XES and R-VtC-XES has been fourth period (i.e. first row) transition metals^42,43,49–52^.

**Fig. 2:**
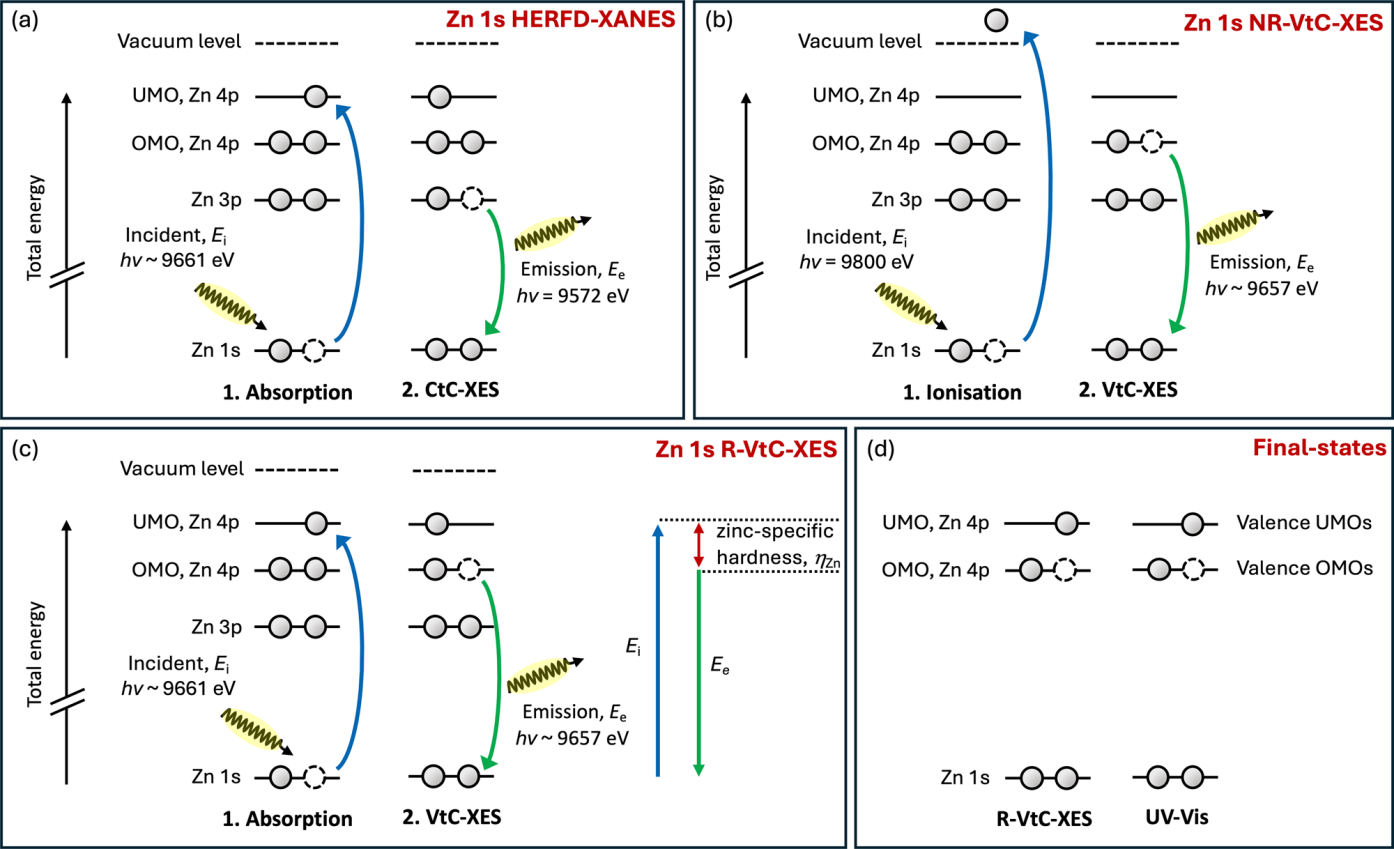
Probing electronic transitions in diorganozinc complexes. A new spectroscopic approach for ZnR_2_. Electronic transition schematic of ZnR_2_ complexes studied: **a** Zn 1s HERFD-XANES, **b** Zn 1s NR-VtC-XES, **c** Zn 1s R-VtC-XES, **d** final-states of Zn 1s R-VtC-XES and UV-Vis spectroscopy.

In part one of our article, we determine *speciation and geometric structure* for 14 ZnR_2_ using a combination of synchrotron-based Zn 1s high-energy-resolution fluorescence detected (HERFD)-XANES spectroscopy (Fig. 2a), density functional theory (DFT) and time-dependent DFT (TDDFT) calculations. To produce intrinsic descriptors, the structure of ZnR_2_ must be known, so the influence on reactivity of steric factors relative to electronic structure factors can be considered. Synthetic chemists are in the very unusual position where the starting geometric structures of ZnR_2_ in toluene/hexane^9–12^ are poorly understood; indeed, the geometric structures of ZnR_2_ “*reagents in non-coordinating solvents remain scattered and elusive*”^53^. The ^67^Zn nucleus (spin 5/2) possesses low NMR sensitivity and gives very broad features so geometric structural conclusions cannot be drawn. Other NMR active nuclei (e.g. ^1^H and ^13^C) are distant from the reactive Zn centre and, coupled with the nature of NMR (through-bond and through-space effects), makes relating chemical shift with geometric structure unreliable. Linearity (i.e. C-Zn-C angle of 180°) has been inferred for some ZnR_2_ using indirect methods, e.g. melting points, boiling points, solubility, and dipole moment determination^54,55^. In contrast, diffusion-ordered spectroscopy (DOSY) NMR studies in [D_8_]toluene suggested that ZnPh_2_ can form dinuclear (ZnPh_2_)_2_^9^, although the relatively high atomic weight of zinc and elongated shape of ZnPh_2_ make this interpretation very uncertain^9,56,57^. Furthermore, in the solid-phase, ZnPh_2_ displayed dinuclear three-coordinate bridging (ZnPh_2_)_2_^54^. XANES spectroscopy showed that ZnEt_2_ is linear in toluene (i.e. C-Zn-C angle of 180°), but no other R substituents were studied^45^.

In part two of our article, we use a combination of NR-VtC-XES (Fig. 2b) and Zn 1s HERFD-XANES spectroscopy (Fig. 2a) to identify the zinc-containing occupied molecular orbitals (OMOs) and unoccupied molecular orbitals (UMOs). This identification is essential for producing zinc-specific descriptors, and also allows us to highlight the OMOs and UMOs that give ZnR_2_ their remarkable reactivity compared to other common zinc-containing species such as [Zn(OH_2_)_6_]^2+^ and ZnCl_2_(THF)_2_.

In part three of our article, we describe a *new approach* using zinc-specific X-ray spectroscopy experiments and calculations to produce three zinc-specific (*i.e*. local), intrinsic^36^ descriptors: zinc-specific *hardness* (*η*_Zn_), zinc-specific *absolute electronegativity* (*χ*_Zn_, the negative of the zinc-specific electronic chemical potential, *μ*_Zn_) and zinc-specific global electrophilicity index (*ω*_Zn_). To obtain *η*_Zn_, *χ*_Zn_ and *ω*_Zn_, we combine our knowledge on experimental R-VtC-XES (Fig. 2c), zinc-containing OMOs and UMOs, and DFT calculations (underpinned by the theories of Pearson^37,39,40^).

## Results and discussion

### Speciation and geometry

**Fig. 3:**
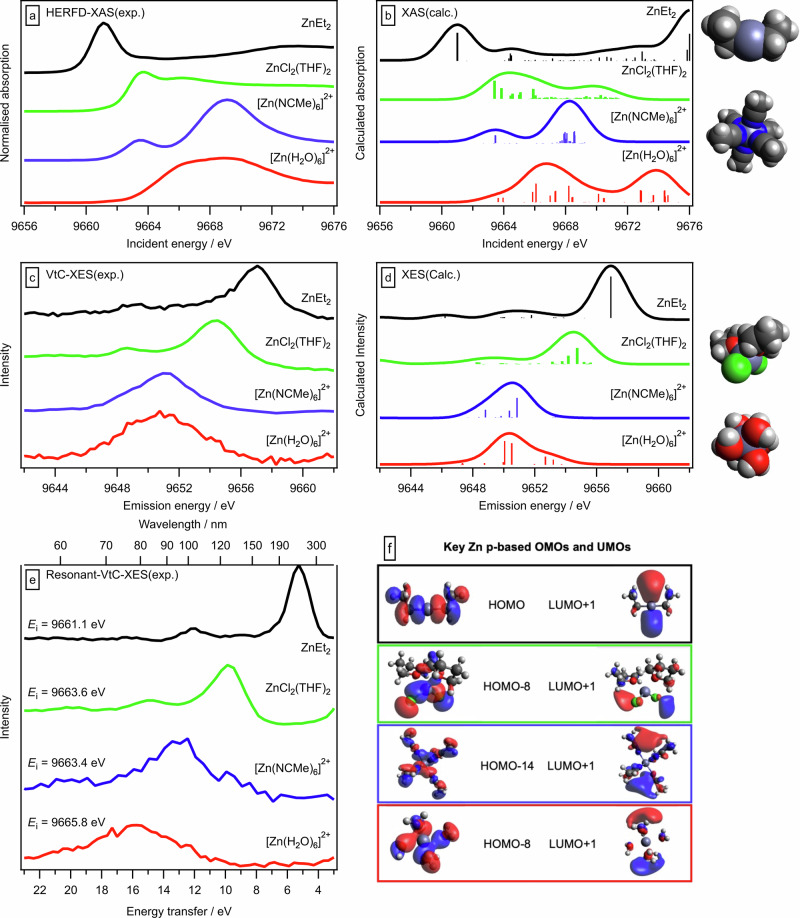
Zn 1s HERFD-XANES, NR-/R-VtC-XES, and DFT calculated spectra of ZnEt_2_ and common zinc-containing compounds. 0.1 M ZnEt_2_ in toluene, ZnCl_2_(THF)_2_ (0.1 M ZnCl_2_ in THF), [Zn(NCMe)_6_]^2+^ (0.1 M Zn(OTf)_2_ in MeCN) and [Zn(OH_2_)_6_]^2+^ (0.1 M ZnCl_2_ in H_2_O): **a** Zn 1s HERFD-XANES spectra (y intensities normalised), **b** TDDFT calculations of Zn 1s HERFD-XANES (shifted by –8.90 eV to visually match experimental Zn 1s HERFD-XANES spectra^69^), **c** Zn 1s NR-VtC-XE spectra, **d** KS-DFT calculations of Zn 1s NR-VtC-XE spectra (shifted by −8.90 eV to visually match experimental Zn 1s NR-VtC-XE spectra). **e** R-VtC-XE spectra. **f** Visual representations for key Zn p-based OMOs and UMOs; for ZnEt_2_ the LUMO + 2 is degenerate (or near-degenerate) with another UMO (Supplementary Fig. 8).

**Fig. 4:**
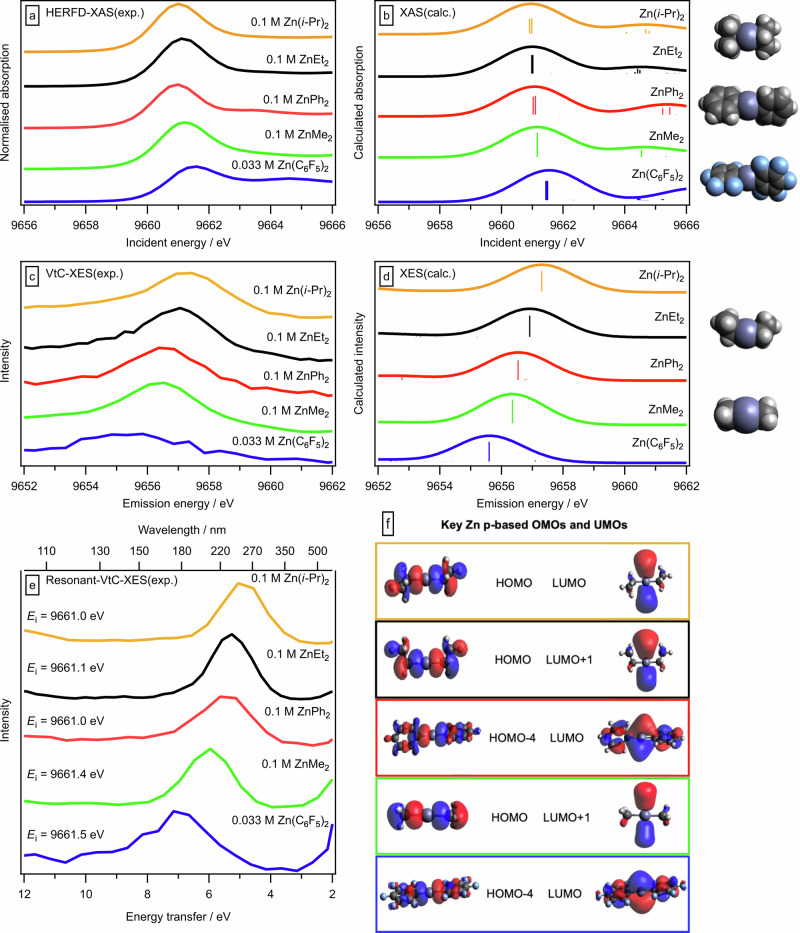
Zn 1s HERFD-XANES, NR-/R-VtC-XES, and DFT calculated spectra of ZnR_2_ (R = Me, Et, *i*-Pr, C_6_F_5_) complexes. Experimental and calculated X-ray spectra for the diorganozinc compounds ZnR_2_ (R = Me, Et, *i*-Pr, Ph, C_6_F_5_, concentration 0.1 M for all apart from C_6_F_5_ which was 0.033 M); calculated C_6_F_5_ shown here is the staggered conformer. **a** Zn 1s HERFD-XANES spectra (y intensities normalised), **b** TDDFT calculations of Zn 1s XANES spectra (shifted by −8.90 eV to visually match experimental Zn 1s HERFD-XANES spectra^69^), **c** Zn 1s NR-VtC-XE spectra, **d** KS-DFT calculations of Zn 1s NR-VtC-XE spectra (shifted by −8.90 eV to visually match experimental Zn 1s NR-VtC-XE spectra), **e** Zn 1s R-VtC-XE spectra, **f** Visual representations for key Zn p-based OMOs and UMOs; for ZnR_2_ the UMO shown here is degenerate (or near-degenerate) with another UMO (Supplementary Fig. 8).

All ZnR_2_ compounds (typically 0.1 M) in toluene studied here have a *linear* C-Zn-C structure, *i.e*. a C-Zn-C angle of 180°. The Zn 1s HERFD-XANES spectra for all ZnR_2_ in toluene (Figs. 3a, 4a and Supplementary Fig. 1) and ZnEt_2_ in hexane (Supplementary Fig. 2) gave a single sharp, intense peak at a low incident energy of ~9661 eV. This common motif for linear two-coordinate complexes, e.g. copper complexes^58^, has previously been observed for ZnEt_2_ in toluene using standard resolution XANES spectroscopy^45^, and is due to two absorption transitions; Zn 1s to two degenerate UMOs with very strong Zn p contributions (discussed further below). In contrast to linear ZnR_2_, the Zn 1s HERFD-XANES spectra for octahedral [Zn(NCMe)_6_]^2+^ and [Zn(OH_2_)_6_]^2+^ and tetrahedral ZnCl_2_(THF)_2_ gave broader, lower intensity peaks at higher incident absorption energy (Fig. 3a). The excellent visual match of experimental Zn 1s HERFD-XANES spectra (Figs. 3a, 4a) and calculated XANES spectra (Figs. 3b, 4b), give high confidence that we have captured the speciation and geometric structure correctly in our calculations. These linear structures in relatively non-coordinating solvents are analogous to gas- and solid-state linear structures for ZnR_2_, i.e. in the absence of any solvent molecules^38,59^.

There is the potential for liquid-phase flex in the C-Zn-C linear bonds for ZnR_2_ away from a C-Zn-C angle of 180°. Using calculated total energies for ZnEt_2_, the structure at 180° is the most stable; decreasing the C-Zn-C angle decreases the structure stability (Supplementary Table 1 and Supplementary Fig. 3). However, when the C-Zn-C angle is greater than 160° the calculated XANES spectra are very similar to that for the structure with C-Zn-C angle at 180° (Supplementary Fig. 3); our experimental data cannot distinguish between structures with a C-Zn-C angle of between 180° and 160°. Furthermore, the calculated total energies for structures with a C-Zn-C angle greater than 160° are within 10 kJ mol^−1^; therefore, whilst the structure at C-Zn-C angle of 180° is the most stable, we cannot rule out slight flexing of the C-Zn-C angle away from 180° (i.e. linear) in the liquid-phase. The dihedral angles (eclipsed 0° or staggered 90°) between the two R substituents gave, within calculated uncertainty, the same total energies (Supplementary Figs. 4,5 and Supplementary Table 2–4). Therefore, both conformers are very likely to be present in the solution for ZnR_2_.

No evidence for the existence of bimetallic bridging structures was found. The calculated XANES spectrum for dimeric (ZnPh_2_)_2_, based on a solid-state structure^54^, shows a clear loss of the sharp, intense peak at low incident energy (Supplementary Fig. 6). This is as expected for a three-coordinate, non-linear zinc centre, as the UMO degeneracy is lifted compared to two-coordinate linear ZnPh_2_^58^. Furthermore, all ZnR_2_ compounds studied here gave very similar experimental and calculated Zn 1s HERFD-XANES spectra with relatively sharp absorption features at low incident energy, showing that bridging species were not present in significant concentration.

Electronic-structure-based descriptors will explain the reactivity of two-coordinate, linear ZnR_2_ because the zinc centre is sterically accessible. The vdW space filling structures for ZnR_2_ studied here, even Zn(C_6_F_5_)_2_ and Zn(*t*-Bu)_2_ with their relatively bulky ligands, all have a clearly visible zinc centre (Figs. 3, 4, right-hand side, Supplementary Fig. 7). In contrast, for the four- and six-coordinate species, the zinc centre is shielded by the ligands and is not visible in the vdW space filling structures (Fig. 3, right-hand side). Therefore, steric hindrance by the R substituents on ZnR_2_ is unlikely to be a factor in deciding reactivity at the zinc, especially when the zinc centre is acting as an electron acceptor. Furthermore, all ZnR_2_ in non-coordinating solvents are coordinatively unsaturated. Hence, all ZnR_2_ can form a bond to an electron donor ligand without the need to first break a Zn-ligand bond, e.g. ZnMe_2_ + THF to form ZnMe_2_(THF)_2_^60^. Furthermore, binding of a chiral Lewis donor substrate/ligand to the coordinatively unsaturated zinc centre has been shown to allow a range of stereoselective reactions to be performed^26,61^.

### Molecular orbital identification—Why are ZnR_2_ so remarkable?

Given the importance of the sterically accessible zinc centre in all ZnR_2_, it is vital to build a picture of the frontier molecular orbitals with significant zinc contributions, as these MOs will control reactivity instead of stereochemical factors. We will demonstrate why the remarkable reactivity of ZnR_2_ occurs relative to other common zinc-containing species.

For all ZnR_2_ where R = alkyl the only MO with a significant Zn p contribution ( ~ 16% Zn p, Supplementary Table 5) is the HOMO (Fig. 4f and Supplementary Figs. 9–14). This is a remarkable result given that the formal oxidation state is Zn^2+^, with the expectation being that electron density of frontier OMOs would reside mainly on the ligands. For R = aryl the OMO,Zn p is the HOMO-4 (with the HOMO to HOMO-3 from the aryl substituents, Fig. 4f, Supplementary Table 5 and Supplementary Figs. 15–24). These identifications are based on the intense, narrow, high energy peak observed for all ZnR_2_ in both the experimental (Fig. 3c, Fig. 4c) and calculated (Fig. 3d, Fig. 4d) Zn 1s NR-VtC-XE spectra (the selection rules for Zn 1s NR-VtC-XES are OMO,Zn p to Zn 1s^35,47,48^), along with a visual analysis of the MO, which shows a clear sigma bond along the axis of the linear C-Zn-C bonds (Fig. 3f, 4f). In contrast, for the 4/6-coordinate zinc-containing species, the main zinc contributions were for multiple OMOs far deeper than the HOMO, captured by the broader and lower energy peaks in Zn 1s NR-VtC-XE spectra (Fig. 3c), e.g. for [Zn(OH_2_)_6_]^2+^ the HOMO-8 to HOMO-10 each had ~6% Zn p contribution (Supplementary Table 6). The OMO,Zn p is very likely to be more influential on reactivity than OMO, Zn s, as the only OMO with a strong Zn s contribution is HOMO-1 for ZnR_2_ (where R = alkyl) and HOMO-5 for ZnR_2_ (where R = aryl) (Supplementary Figs. 25–28 and Supplementary Table 5), which are ~1.5 eV deeper than the single Zn p OMO.

The three lowest energy UMOs (LUMO, LUMO + 1 and LUMO + 2) for almost all ZnR_2_, and also the common zinc-containing species, were composed of two Zn p-based UMOs and one Zn s-based UMO (Figs. 3f, 4f, Supplementary Figs. 9–24 and Supplementary Table 5). This identification is based on a combination experimental and calculated Zn 1s HERFD-XANES spectra plus ground state DFT calculations. All three of these UMOs for ZnR_2_ are similar in energy (Supplementary Figs. 25–28). The two degenerate (or near-degenerate) Zn p-containing UMOs have Zn p contributions of ~75% (R = alkyl) and ~40% (R = aryl), all perpendicular to C-Zn-C bond (Figs. 3f, 4f and Supplementary Table 5).

Our results demonstrate that Zn p orbitals contribute strongly to both OMOs and UMOs that are key in bond breaking and forming, contradicting theoretical findings which suggested that Zn p contributions could be neglected for zinc-containing species, including ZnR_2_^62,63^. Furthermore, previous reports utilising a simplified hypervalent scheme, with no Zn p orbital contributions for ZnMe_2_^63^, were clearly too much of a simplification.

Overall, the UMOs for ZnR_2_ are unremarkable compared to common zinc-containing species, highlighting that the electronically unusual states for ZnR_2_ are the OMOs. Therefore, the strong reactivity of ZnR_2_ compared to common zinc-containing species comes mainly from a combination of the OMOs and the two-coordinate structure.

### Quantifying the effect of R on ZnR_2_ reactivity: three new descriptors

To capture the zinc-specific, intrinsic *hardness*, *η*_Zn_, our first new descriptor, we use the experimental Zn 1s R-VtC-XES peak energy (Fig. 2c), which represents the experimental energy gap between OMO,Zn p and UMO,Zn p, *E*(gap,exp). *E*(gap,exp) captures the Zn p-specific energy gap *η*_Zn_ = *E*(OMO,Zn p)—*E*(UMO,Zn p), where *E*(OMO,Zn p) and *E*(UMO,Zn p) represent the energies of the OMO and UMO with strongest Zn p contribution, respectively. Very importantly, the final-state electronic structure of R-VtC-XES is analogous to UV-Vis spectroscopy (Fig. 2d)^42^, i.e. one electron in an UMO bound state and a hole in a valence OMO, but R-VtC-XES is an overall two-photon process which can bypass the dipole selection rules of UV-Vis spectroscopy to effectively access the optically dark Zn p to Zn p transitions. Therefore, our energy gap is essentially a zinc-specific, experimental version of the Pearson *η* (which is *η* = (*E*(HOMO) − *E*(LUMO))/2)^37^). A small zinc-specific *η*_Zn_ means relatively strong Zn-reactant molecule covalent interactions; large zinc-specific *η*_Zn_ means relatively strong Zn-reactant molecule electrostatic interactions, i.e. more ionic. In addition, we have used both DFT and multiconfigurational, multistate-restricted active space second-order perturbation theory (MS-RASPT2) calculations to guide our approach.

**Fig. 5:**
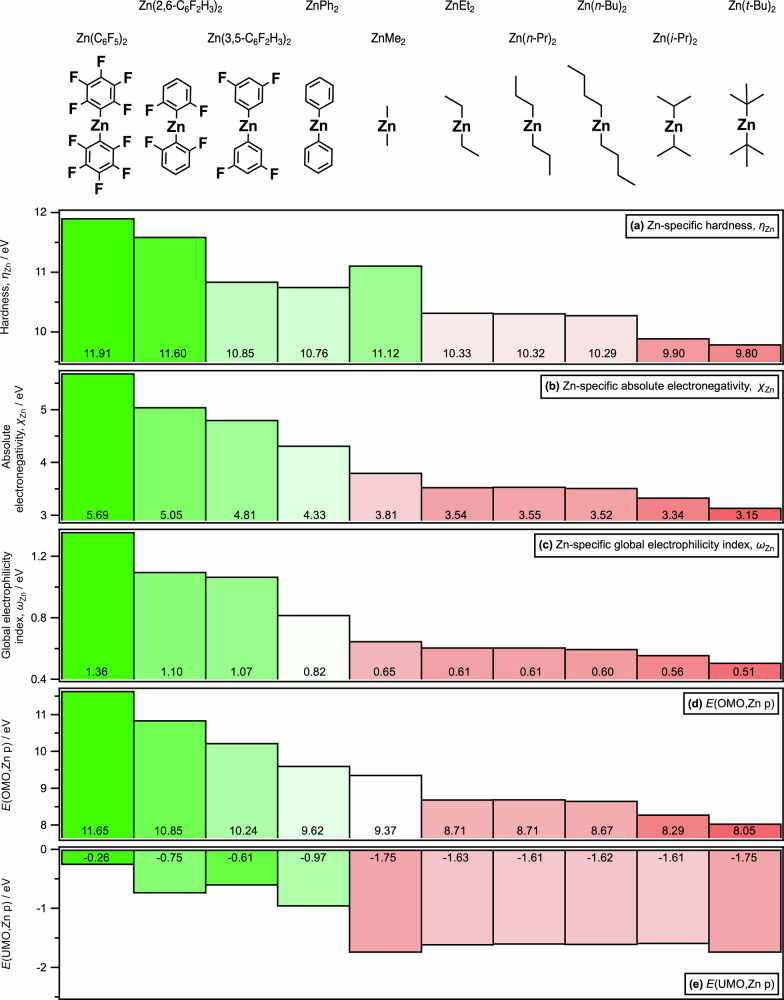
Computed Zn-specific electronic structure descriptors: *η*_Zn_, *χ*_Zn_, *ω*_Zn_, *E*(OMO,Zn p), and *E*(UMO,Zn p). Electronic structure properties for ZnR_2_: **a** zinc-specific hardness (*η*_Zn_), **b** zinc-specific absolute electronegativity (*χ*_Zn_), **c** zinc-specific global electrophilicity index (*ω*_Zn_), **d ***E*(OMO,Zn p), **e** *E*(UMO,Zn p). Tabulated values available in Supplementary Table 7.

**Fig. 6:**
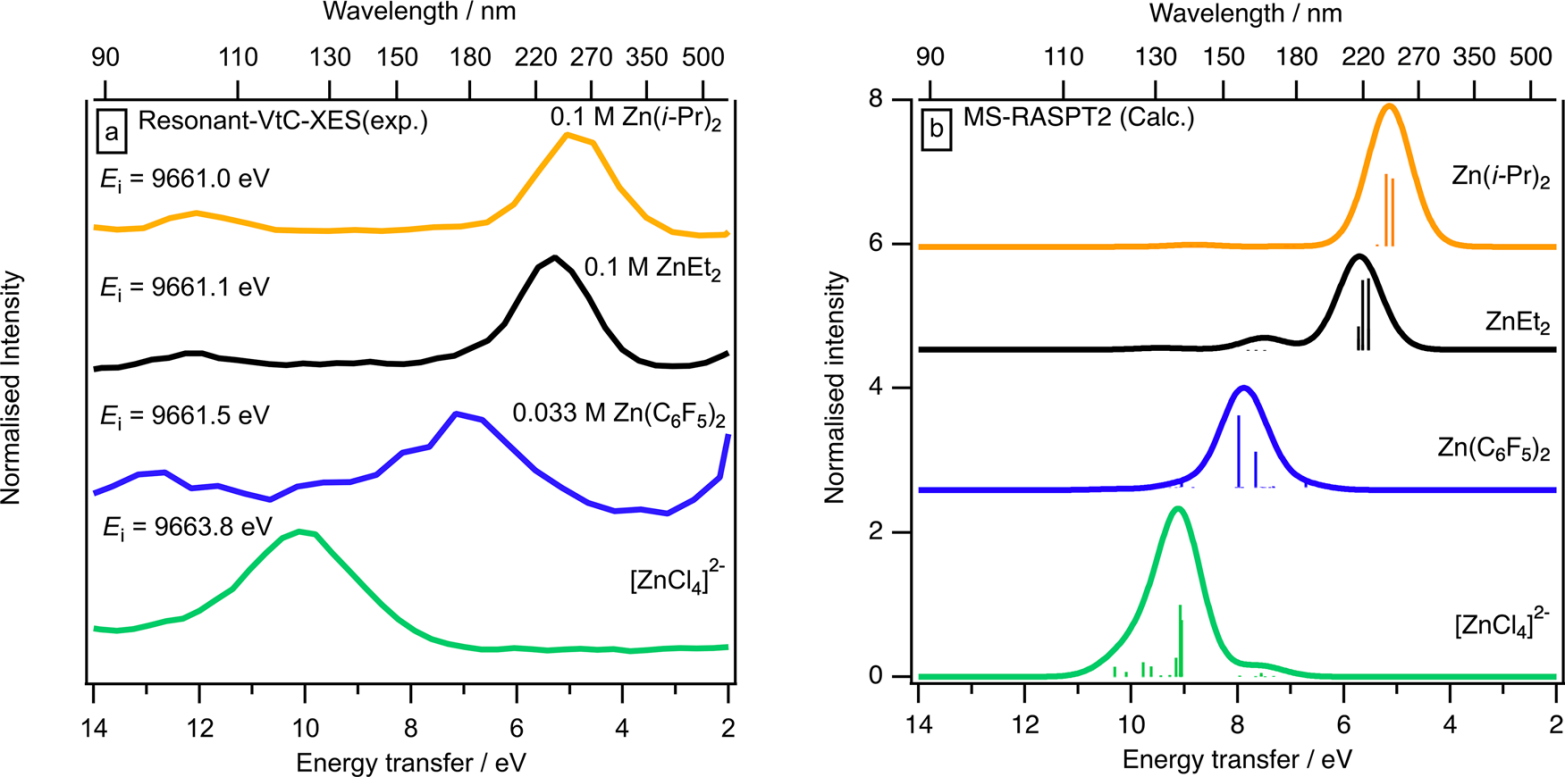
Experimental and calculated spectra of ZnR_2_ (R = Me, Et, *i*-Pr, C_6_F_5_) and [ZnCl_4_]^2-^. Comparison of experimental and calculated R-VtC-XE spectra: **a** experimental spectra for ZnR_2_ (R = Me, Et, *i*-Pr and C_6_F_5_; concentration 0.1 M for all except C_6_F_5_, which was 0.03 M) and *x* = 0.33 ZnCl_2_ in [C_8_C_1_Im]Cl (which gives only [ZnCl_4_]^2-^ in solution^47,69^); **b** Calculated RASPT2 (MS-RASPT2(12,1,1;1,5,3) ANO-RCC-VTZP) spectra for ZnR_2_ (R = Et, *i-*Pr and C_6_F_5_; the staggered conformer of C_6_F_5_ was used) and [ZnCl_4_]^2-^. All calculated spectra include Gaussian broadening with a full width at half maximum (FWHM) of 2.5 eV.

**Fig. 7:**
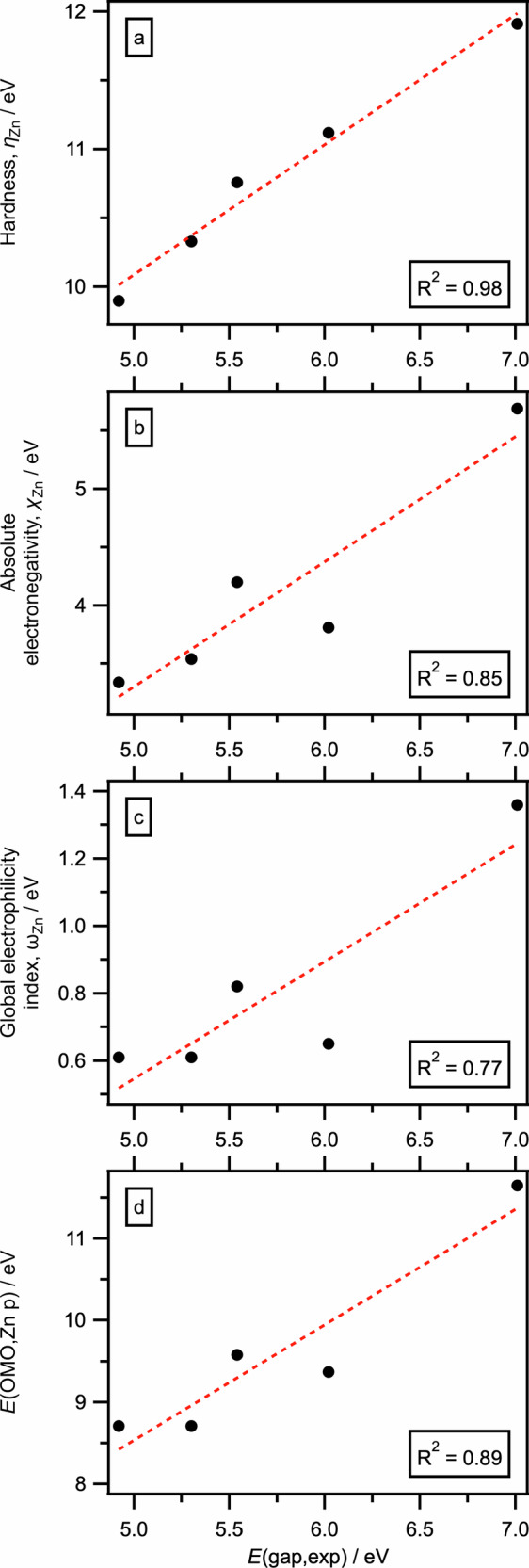
Computed Zn-specific descriptor correlations with experimental R-VtC-XES gap. Linear correlation fits for calculated descriptors against experimental R-VtC-XES energy gap (*E*(gap,exp)): **a** zinc-specific hardness (*η*_Zn_) versus *E*(gap,exp); **b** zinc-specific absolute electronegativity (*χ*_Zn_) versus *E*(gap,exp); **c** zinc-specific global electrophilicity index (*ω*_Zn_) versus *E*(gap,exp) and **d ***E*(OMO,Zn p) versus *E*(gap,exp).

The experimental R-VtC-XES-derived descriptor *η*_Zn_ was: Zn(C_6_F_5_)_2_ ≫ ZnMe_2_ > ZnPh_2_ > ZnEt_2_ > Zn(*i*-Pr)_2_ (Fig. 4e and Supplementary Fig. 29). Based on a combination of experimental and calculated data, the zinc-based *η*_Zn_ for ZnR_2_ is: Zn(C_6_F_5_)_2_ > Zn(2,6-F_2_C_6_H_3_)_2_ > Zn(3,5-F_2_C_6_H_3_)_2_ > ZnMe_2_ > ZnPh_2_ > ZnEt_2_ ≈ Zn(*n*-Pr)_2_ ≈ Zn(*n*-Bu)_2_ > Zn(*i*-Pr)_2_ > Zn(*t*-Bu)_2_ (Fig. 5a). This experimental *η*_Zn_ can only be measured using R-VtC-XES as the experimental energy gaps for ZnR_2_ correspond to ~175 nm to ~250 nm respectively (Fig. 4e), which cannot be measured using standard UV-Vis spectroscopy due to the energy gap being too large (for peaks up to ~200 nm) and the peaks being overwhelmed by the toluene solvent (for peaks up to ~285 nm)^64^. Furthermore, this experimental R-VtC-XES-derived *η*_Zn_ effectively captures a Zn p to Zn p transition, rather than the classic *E*(OMO) – *E*(UMO) energy gap measured using UV-Vis spectroscopy (Fig. 2d). MS-RASPT2 R-VtC-XES calculations, where the valence hole is included, match both the experimental R-VtC-XES energy gap order and absolute energy gap values for ZnR_2_ (Fig. 6), demonstrating that the R-VtC-XES calculations capture the same physical effect as the experiments. Whilst an excellent match, these MS-RASPT2 calculations are time intensive and technically challenging. The ground state Zn p-specific *η*_Zn_ = *E*(OMO,Zn p) – *E*(UMO,Zn p) energy gap order (but not the absolute energies) is the same as observed using both experimental R-VtC-XES (Fig. 7a, R^2^ = 0.98, Supplementary Fig. 32), demonstrating that the much cheaper and simpler ground state calculations capture *η*_Zn_.

We calculate a zinc-specific, intrinsic *absolute electronegativity*, *χ*_Zn_ = (*E*(OMO,Zn p) + *E*(UMO,Zn p)) / 2, our second new descriptor. These DFT calculations are validated against data from both Zn 1s HERFD-XANES spectroscopy and NR-VtC-XES (Fig. 3b, 3d); we have high confidence in given the excellent matches of our experimental and calculated spectra (Fig. 3 and Fig. 4). A large (or small) zinc-specific *χ*_Zn_ corresponds to a strong Lewis acidic (or Lewis basic) zinc centre.

Using both *η*_Zn_ and *χ*_Zn_, a zinc-specific version of the global electrophilicity index (*ω*)^39,40^, where *ω*_Zn_ = (*χ*_Zn_)^2^ / (2 × *η*_Zn_), was calculated, our third new descriptor. A large (or small) zinc-specific *ω*_Zn_ corresponds to a strong Lewis acidic (or Lewis basic) zinc centre.

The zinc-specific descriptors *χ*_Zn_ and *ω*_Zn_ for ZnR_2_ are: Zn(C_6_F_5_)_2_ > Zn(2,6-F_2_C_6_H_3_)_2_ > Zn(3,5-F_2_C_6_H_3_)_2_ > ZnPh_2_ > ZnMe_2_ > ZnEt_2_ ≈ Zn(*n*-Pr)_2_ ≈ Zn(*n*-Bu)_2_ > Zn(*i*-Pr)_2_ > Zn(*t*-Bu)_2_ (Fig. 5b, Fig. 5c, Supplementary Fig. 29 and Supplementary Table 7). *χ*_Zn_ and *ω*_Zn_ correlate linearly (R^2^ = 0.99, Supplementary Fig. 33c), demonstrating that either can be used for quantifying Lewis acidity/basicity strength. Our results match quantitative reports on ZnR_2_ and SacNacZnR Lewis acidity of Zn(C_6_F_5_)_2_ > ZnPh_2_^31,65^. Our results also match qualitative reports from empirical patterns of reactivity; ZnEt_2_ as a relatively weak/mild Lewis acid^65^ compared to ZnPh_2_ and Zn(C_6_F_5_)_2_^10,19^, and Zn(C_6_F_5_)_2_ as a strong Lewis acid^9,12^. Results from DFT calculations suggest that ZnMe_2_ can act as a nucleophile^4^; our results show that any other ZnR_2_ (where R = alkyl) would be a better nucleophile than ZnMe_2_ (ignoring steric constraints).

Using zinc-specific *η*_Zn_, *χ*_Zn_ and *ω*_Zn_ descriptors means we directly capture the electronic structure of the location where both electron acceptance (controlling Lewis acidity) and electron donation (controlling Lewis basicity^4^) occur for ZnR_2_, i.e. the zinc centre. This approach removes the problem of ligand electronic contributions clouding production of the descriptors, ensuring that comparable and consistent values for different R substituents are produced. Furthermore, our zinc-specific approach means that the zinc spectroscopic contributions are not overwhelmed by solvent/ligand contributions.

The *η*_Zn_, *χ*_Zn_ and *ω*_Zn_ descriptors show that strong Lewis acids are hard, and strong Lewis bases are soft. Zn(C_6_F_5_)_2_ is a hard, strong Lewis acid/electrophile and Zn(*t*-Bu)_2_ is a soft, strong Lewis base/nucleophile.

*χ*_Zn_ and *ω*_Zn_ descriptors exhibit a strong correlation with the R Taft inductive substituent constant, *I*_R_, for ZnR₂; as evidenced by a high R² value. (Supplementary Fig. 34 and Supplementary Table 7). The ability of substituent R to donate/withdraw electron density to/from the zinc centre explains ZnR_2_ reactivity. The alkyl chain length makes very little difference for *n*-alkyl from Et onwards. This finding matches to results from gas-phase photoelectron spectroscopy of four ZnR_2_ (R = alkyl)^38^. For the partially fluorinated Ph, *χ*_Zn_ increased fairly consistently with the increasing number of F substituents, e.g. Zn(4-FC_6_H_4_)_2_ has a smaller *χ*_Zn_ than Zn(3,5-F_2_C_6_H_3_)_2_; the positioning of the fluorine on aryl matters: *ortho* has the largest effect relative to ZnPh_2_, with a weaker effect for *meta* (Supplementary Fig. 29).

*η*_Zn_, *χ*_Zn_ and *ω*_Zn_ descriptors have almost the same order (Fig. 5a-c, Supplementary Fig. 33), and correlate well with *E*(gap,exp) (Fig. 7). Therefore, our *experimentally measured* R-VtC-XES Zn p-specific energy gap *E*(gap,exp), i.e. *η*_Zn_, can be used with a good level of confidence as a single experimental descriptor to quantify the effect of substituent R on ZnR_2_ reactivity. Furthermore, *η*_Zn_, *χ*_Zn_ and *ω*_Zn_ descriptors are influenced strongly by *E*(OMO,Zn p) (Fig. 5 and Supplementary Fig. 35). Therefore, *E*(OMO,Zn p) can be used as a single descriptor to quantify the effect of substituent R on ZnR_2_ reactivity; *E*(OMO,Zn p) can be readily calculated—a ground state DFT calculation, then straightforward visual identification of OMO Zn p for ZnR_2_—allowing accessible in silico screening of ZnR_2_ species. Even simpler, *χ*_Zn_ and *ω*_Zn_ can be predicted if the induction ability of the R substituent is known (Supplementary Fig. 34).

### Conclusions and future work

Our combination of three synchrotron-based, solution-phase, zinc-specific *X-ray spectroscopies* coupled with calculations allows the comprehensive, systematic and quantitative elucidation of the electronic properties that define the reactivity of synthetically-relevant ZnR_2_. We have unambiguously and directly demonstrated that a range of ZnR_2_ reagents, in the solvents toluene and hexane, adopt a linear two-coordinate geometry, which establishes that electronic structure will be the primary controller of reactivity. We provide new, intrinsic, quantitative Lewis acidity/basicity descriptors for liquid-phase ZnR_2_ compounds, which opens up the possibility of fine-tuning ZnR_2_ reactivity and greatly reduces the need for empirical, iterative synthetic experimentation. Zinc-specific hardness, equivalent to the experimental energy gap between OMO,Zn p and UMO,Zn p, quantifies the chances of forming a more covalent or electrostatic bond with a reactant molecule. Zinc-specific absolute electronegativity and zinc-specific global electrophilicity index both quantify electron acceptor (*i.e*. Lewis acidity/electrophilicity) and electron donor (i.e. nucleophilicity/nucleophilicity) abilities. For rapid screening, both the induction strength of the R substituents of ZnR_2_ and the energy of the calculated OMO,Zn p, *E*(OMO,Zn p), are excellent descriptors for quantification of both zinc-specific hardness and absolute electronegativity, of potential use within the wider catalysis and FLP communities. These new empirical zinc-specific descriptors also provide excellent inputs for quantitative structure-property relationships (QSAR) or for reaction discovery/development using automated synthesis machines.

A combination of factors give rise to the remarkable reactivity of ZnR_2_ over common zinc-containing species. Firstly, the easily ionised Zn p-containing OMO; secondly, ZnR_2_ electronic softness; thirdly, the sterically accessible Zn p-containing UMOs due to the linear ZnR_2_ geometric structure.

Our *liquid-phase* and *zinc-specific* suite of X-ray spectroscopy techniques allow us to side-step many common spectroscopic issues for zinc in solution. Zn 1s R-VtC-XES, which we demonstrate the use of for the first time here, is incredibly powerful for studying zinc as we can *exclusively* focus on the electronic structure associated with the zinc centre rather than that of the associated ligands. R-VtC-XES effectively provides a *zinc-specific* version (with different selection rules) of UV-Vis spectroscopy that avoids the classic problems of solvent windows or ligand absorption. Therefore, R-VtC-XES is suitable for a vast range of functional zinc species, from enzymes through to batteries and under the diverse conditions they would encounter. In the area of organometallics, future work could focus on donor/coordinating solvents^60^, potentially intramolecularly coordinating R substituents (e.g. Zn[(CH_2_)_3_X]_2_ where X = SCH_3_, N(CH_3_)_2_)^66^, zincates/dinuclear complexes^67^, and zinc bis-amides^68^.

Our *liquid-phase* and *element-specific* suite of X-ray spectroscopy techniques offers exceptional opportunities for capturing geometric and electronic structure and producing descriptors for interpreting and predicting reactivity, for the first time for many elements. Plenty of XANES, NR-VtC-XES and R-VtC-XES studies exist for fourth period (i.e. first row) transition metals^42,43,49–52^, but studies for other elements are limited. Elements for which this approach is particularly attractive are those with oxidation states that give closed-shell d^10^, where p orbitals are crucial for bonding, i.e. main-group elements in the latter part of the fourth period and the start of the fifth period: Ga, Ge, As, Se, Br, Rb, Sr.

## Methods

### Sample preparation

All samples were prepared in a glove box, under inert conditions (nitrogen, H_2_O < 0.1 ppm, O_2_ < 0.5 ppm). 1 M diethylzinc (ZnEt_2_) in hexanes, 2 M dimethylzinc (ZnMe_2_) in toluene, 1 M di*iso*propylzinc (Zn(*i*-Pr)_2_) in toluene, diphenylzinc (ZnPh_2_) (98%), bis(pentafluorophenyl)zinc (Zn(C_6_F_5_)_2_) (98%), anhydrous zinc trifluoromethanesulfonate (Zn(TfO)_2_) (98%), and anhydrous zinc chloride (ZnCl_2_) (99%) were obtained from Sigma-Aldrich. 1-octyl-3-methylimidazolium chloride ([C_8_C_1_Im]Cl) (99%) was obtained from Iolitec. Neat solvents toluene, tetrahydrofuran and acetonitrile were obtained from Sigma-Aldrich. To avoid self-absorption, solutions were made/diluted to 0.1 M. Zn(C_6_F_5_)_2_ was prepared at 0.033 M due to the presence of insoluble matter at 0.1 M. Zinc tetrachloride, [ZnCl_4_]^2-^, was prepared from a mixture of ZnCl_2_ (0.838 g, 6.15 mmol) in [C_8_C_1_Im]Cl (2.882 g, 12.49 mmol) to the desired mole fraction *x* = 0.33, i.e. 2 × [C_8_C_1_Im]Cl + ZnCl_2_ → [C_8_C_1_Im]_2_[ZnCl_4_], following literature procedures^47,69^. The samples were prepared within plastic centrifuge vials (*ca*. 0.5 ml to 1.0 ml), sealed, and transported to the I20-Scanning experimental hutch. The samples were held in front of the X-ray beam either in a small plastic vice at the top and bottom; or the centrifuge vial was held with a small ring (Supplementary Fig. 36).

### HERFD-XAS experiments

X-ray absorption (XA) spectra were collected at Diamond Light source I20-Scanning^70^. A Si(111) four-bounce monochromator (energy range: Si(111): incident energy, *E*_i_ = 4 eV to 20 keV; energy resolution: δ*E*/*E* = 1.3×10^-4^) was used to scan from *E*_i_ = 9600 eV to 9900 eV. XA spectra were collected using an X-ray emission spectrometer^71^, and by fixing the emission energy to monitor the relaxation of the Kβ_1,3_ emission line at emission energy, *E*_e_ = 9572 eV. Scanning parameters were as follows: the pre-edge region (*E*_i_ = 9600 eV to 9650 eV) in 5.0 eV steps, the edge region *E*_i_ = 9650 eV to 9675 eV) in 0.3 eV steps, and the post-edge region (*E*_i_ = 9675 eV to 9698 eV) in 0.6 eV steps, and the post-XANES region (*E*_i_ = 9698 eV to 9750 eV in 1.6 eV steps; *E*_i_ = 9750 eV to 9900 eV in 2.0 eV steps, respectively). Samples were orientated at a 90° angle with respect to the incident beam at ambient temperature and pressure. Incident beam flux was lowered through attenuation (aluminium 0.2 mm, carbon 2.0 mm) to minimise radiation damage. XANES spectra were monitored throughout all scans to determine any degradation or damage to the sample over time. The energy of the monochromator was calibrated to the Zn K-edge of a Zn foil (9659.0 eV) (Supplementary Fig. 37).

### XES experiments

X-ray emission (XE) spectra were collected using the X-ray emission spectrometer at Diamond Light source I20-Scanning using three Ge(555) crystal to record the Kβ_2,5_ emission line, i.e. valence-to-core (VtC). For NR-VtC-XE spectra, the incident energy was set to *E*_i_ = 9800 eV, significantly above resonant conditions. For the R-VtC-XE experiments, the incident energy was set to the white-line intensity (near-edge region absorption maxima). Resonant and non-resonant VtC XE spectra were recorded over the emission energy range, *E*_e_ = 9630 eV to 9700 eV.

### Data processing

Experimental XA spectra were averaged and normalised using the Demeter software package, Athena^72^. NR-VtC-XES and R-VtC-XES data processing were performed using Igor Pro 8.0. The recorded emission spectra were adjusted to account for the incident beam energy calibration, ensuring accurate emission energy alignment. This was achieved by fitting a Gaussian distribution to the recorded elastic peak and adjusting so that the emission energy subtracted from incident energy equals zero. This shift was applied to all recorded emission spectra, assuming the spectrometer remained stable during the measurement of each solution studied (Supplementary Table 8).

## Calculations

### Geometry optimisations

All calculations were performed using the ORCA 5.0.3 electronic structure program^73^. Cartesian coordinates for all complexes were built using Avogadro^74^. Geometry optimisations were performed with tight convergence, an ultrafine grid (defgrid3) and without symmetry constraints using a range-separated hybrid functional, ωB97X-D3BJ^75^, with a modified ZORA-def2-TZVPP basis set^76^, paired with a decontracted SARC/J auxiliary basis set^77^. The RIJCOSX approximation was used to speed up optimisations. Scalar relativistic effects were introduced using the zeroth-order regular approximation (ZORA)^78^. Dispersion effects were taken into account using the atom-pairwise dispersion correction with the Becke-Jonson damping scheme. The solvent environment was modelled using the Conductor-like Polarisable Continuum Model (CPCM)^79,80^, *via* the PCM implicit solvent models for toluene, hexane, water, acetonitrile (MeCN), tetrahydrofuran (THF) and ionic liquid (relative permittivity = 11.40)^81^, respectively. Frequency analysis was carried out for all optimised structures, which are confirmed as minima by the absence of imaginary modes. Avogadro was used as an aid in the quantitative analysis of ORCA output files and the visualisation of molecular orbitals.

### XAS and XES calculations

All calculations were performed using the ORCA 5.0.3 electronic structure program^73^. Single point calculations utilising a larger basis set to better capture the electronic structure were performed with time-dependent DFT for XAS and Kohn-Sham DFT for XES, using the ωB97X-D3BJ functional, with a ZORA-def2-QZVPP basis set^76^ and SARC/J auxiliary basis set^77^. In all cases, electric-dipole, magnetic-dipole, and quadrupole contributions were allowed in spectral calculations. Time-dependent DFT calculations were performed with the Tamm-Dancoff approximation (TDA) applied. The solvent environment was modelled using the Conductor-like Polarisable Continuum Model (CPCM)^79,80^, *via* the PCM implicit solvent models for toluene, hexane, water, acetonitrile (MeCN), tetrahydrofuran (THF) and ionic liquid (relative permittivity = 11.40)^81^, respectively. For the XAS calculation, the Zn 1s orbital was excited into all virtual UMOs to mimic the Zn 1s K-edge XAS. Calculated XAS spectra were shifted by −8.90 eV to align with the experimental energies (Supplementary Fig. 38). Ground-state KS-DFT VtC-XES calculations were performed. A single relative shift of −8.90 eV was applied from the difference between the most intense VtC peak in the experiment and the calculation (Supplementary Fig. 39). All computational spectra were generated by convoluting the computed energies and oscillator strengths with Gaussian functions with a full width at half maximum (FWHM) of 2.50 eV.

### MS-RASPT2 calculations

RXES spectra were calculated with the state-averaged restricted active space self-consistent field (SA- RASSCF)^82,83^ method with multi-state restricted active space second-order perturbation theory (MS-RASPT2)^84,85^. Calculations were performed in OpenMolcas^86^, where the core-hole states are generated using the highly excited state scheme (HEXS)^87^ and the transition energies and dipole vectors between states are calculated across a set of biorthonormal orbitals using the restricted active space state interaction method^88^. All calculations use the ANO-RCC-VTZP basis set, which includes relativistic effects^89–92^, and an imaginary shift of 0.1 a.u. was applied to the MS-RASPT2 calculation to remove intruder states. Solvent effects were included using the implicit PCM solvent model for toluene and ionic liquid (relative permittivity = 11.40)^81^. To maintain a consistent level of theory, the RXES calculations of the different zinc complexes use the same active space size. The Zn 1s orbital was placed into RAS1 (maximum number of holes restricted to 1), the highest 5 occupied orbitals were placed into RAS2 (full CI space) and the lowest three unoccupied orbitals were placed into RAS3 (maximum number of electrons restricted to 1). 3 core excited and 15 valence excited states were included in each calculation and approximate relative RIXS intensities were calculated using a previously used simplified formalism of the Kramer-Heisenberg equation which assumes resonant conditions and a constant lifetime-broadening broadening^93–95^.

### Calculation processing

MOAnalyzer was employed to determine the percentage contribution of Zn s/p character within key molecular orbitals involved in both XAS and XES calculations using Löwdin population analysis^96^. MultiWFN was used to visualise the molecular orbitals constituting the occupied valence states^97^; this was achieved by analysing the total density-of-states (tDoS) and partial density-of-states (pDoS), specifically fragmenting both the Zn s/p orbitals.

## Supplementary information

Supplemental Information

## Data Availability

Raw data were collected using the I20-Scanning beamline at Diamond Light Source. Raw data and calculation output files supporting the findings of this study are available at University of Reading Research Data Archive, 10.17864/1947.001460. Calculations performed using ORCA can be reproduced using the provided coordinates and example input files included in the supplementary information. MS-RASPT2 calculation input files can be requested from A.E.A.F.
