# Supplementary material for: Determining structure and Zn-specific Lewis acid-base descriptors for diorganozincs in non-coordinating solvents using X-ray spectroscopy
Source: Commun Chem. 2025 Oct 3;8:294. doi: 10.1038/s42004-025-01704-x (PMC12494758; doi:10.1038/s42004-025-01704-x)
Supplement: Supplementary file 1 — Supplemental Information [file 42004_2025_1704_MOESM1_ESM.pdf]

Supplementary information material for:

Determining structure and Zn-specific Lewis acid-base descriptors for diorganozincs in non-coordinating solvents using X-ray spectroscopy

Lewis G. Parker,[1] Frances K. Towers Tompkins,[1] Jake M. Seymour,[1] Najaat Alblewi,[1] Ekaterina Gousseva,[1] Megan R. Daw,[1] Shusaku Hayama,[2] Richard P. Matthews,[3] Adam E. A. Fouda,[4,5] Joshua D. Elliott,[2] Christopher D. Smith,[1] Kevin R. J. Lovelock[1]

[1] Department of Chemistry, University of Reading, Pepper Lane, Reading, RG6 6DX, UK
[2] Diamond Light Source, Diamond House, Harwell Science and Innovation Campus, Didcot, Oxfordshire, OX11 0DE, UK
[3] Department of Biosciences, University of East London
[4] Department of Physics, The University of Chicago, Chicago, Illinois, 60637, USA
[5] Chemical Sciences and Engineering Division, Argonne National Laboratory, Lemont, Illinois, 60439, USA

Contact E-mail: k.r.j.lovelock@reading.ac.uk
c.d.smith@reading.ac.uk

This pdf includes:

Supplementary tables 1 – 8: pages 1 – 8
Supplementary figures 1 – 39: pages 9– 47
Supplementary notes: pages 48 – 87
Supplementary references: page 88

Supplementary tables

Supplementary Table 1. Calculated $ZnEt_2$ (PCM toluene) bend angle and self-consistent field (SCF) energy.

C-Zn-C bend angle / °	SCF energy / E_h	SCF energy / kJ mol^{-1}	SCF energy difference relative to 180° / kJ mol^{-1}
180	-1937.931040	-5088037.947	0.00
174	-1937.930684	-5088037.011	0.94
168	-1937.929975	-5088035.150	2.80
162	-1937.928594	-5088031.523	6.42
157	-1937.926747	-5088026.673	11.27
151	-1937.924378	-5088020.454	17.49
145	-1937.921562	-5088013.061	24.89
140	-1937.918185	-5088004.195	33.75

Supplementary Table 2. Calculated $ZnEt_2$ (PCM hexane) dihedral angle (C-C-Zn-C-C) and self-consistent field (SCF) energy.

C-C-Zn-C-C dihedral angle / °	SCF energy / E_h	SCF energy / kJ mol^{-1}	SCF energy difference relative to 180° / kJ mol^{-1}
180	-1964.018061	-5156529.419	0.00
165	-1964.018056	-5156529.406	0.01
150	-1964.018056	-5156529.406	0.01
135	-1964.018069	-5156529.439	-0.02
120	-1964.018077	-5156529.461	-0.04
105	-1964.018100	-5156529.520	-0.10
90	-1964.018084	-5156529.479	-0.06

Supplementary Table 3. Calculated $ZnPh_2$ (PCM hexane) dihedral angle (C-C-Zn-C-C) and self-consistent field (SCF) energy.

C-C-Zn-C-C dihedral angle / °	SCF energy / E_h	SCF energy / kJ mol^{-1}	SCF energy difference relative to 178° / kJ mol^{-1}
178	-2269.290069	-5958021.076	0.00
150	-2269.290141	-5958021.266	-0.19
138	-2269.290186	-5958021.385	-0.31
118	-2269.290311	-5958021.711	-0.64
101	-2269.290333	-5958021.771	-0.69

Supplementary Table 4. Calculated staggered and eclipsed conformers of $Zn(C_6F_5)_2$ and $ZnPh_2$ (PCM toluene) and resultant (SCF) energy.

Sample	Orientation	PCM	C-C-Zn-C-C angle / °	SCF energy / E_h	SCF energy / kJ mol^{-1}	SCF energy difference/ kJ mol^{-1}
$ZnPh_2$	Staggered	Toluene	90	-2269.292704	-5958027.995	0.63
	Eclipsed	Toluene	180	-2269.292466	-5958027.371	
$Zn(C_6F_5)_2$	Staggered	Toluene	110	-3263.583982	-8568539.744	0.59
	Eclipsed	Toluene	172	-3263.584204	-8568540.329	

Supplementary Table 5. Calculated valence molecular orbitals for ZnR_2 complexes. Valence molecular orbitals with strong Zn 4p (green) and Zn 4s (red) contributions have been highlighted.

	HOMO-5	HOMO-4	HOMO-3	HOMO-1	HOMO	LUMO	LUMO+1	LUMO+2	LUMO+3	LUMO+4
$ZnMe_2$				Zn s 27%	Zn p 18%	Zn s 59%	Zn p 86%	Zn p 86%		
$ZnEt_2$				Zn s 23%	Zn p 16%	Zn s 49%	Zn p 70%	Zn p 80%		
Zn(*n*-Pr)$_2$				Zn s 21%	Zn p 16%	Zn s 53%	Zn p 76%	Zn p 81%		
Zn(*n*-Bu)$_2$				Zn s 19%	Zn p 15%	Zn s 54%	Zn p 75%	Zn p 80%		
Zn(*i*-Pr)$_2$				Zn s 22%	Zn p 15%	Zn p 75%	Zn s 44%	Zn p 68%		
Zn(*t*-Bu)$_2$				Zn s 22%	Zn p 14%	Zn s 32%	Zn p 66%	Zn p 66%		
stag $ZnPh_2$	Zn s 18.4%	Zn p 15%				Zn p 42%	Zn p 46%	Zn s 54%		
ecl $ZnPh_2$	Zn s 18.3%	Zn p 15%				Zn p 41%	Zn s 52%	Zn p 70%		
ecl $Zn(C_6F_5)_2$	Zn s 23%	Zn p 17%				Zn p 38%	Zn p 38%	Zn s 49%		
stag $Zn(C_6F_5)_2$	Zn s 23%	Zn p 17%				Zn p 35%	Zn p 35%	Zn s 45%		
stag Zn(2,6-$C_6F_2H_3$)$_2$	Zn s 20%	Zn p 16%				Zn p 37%	Zn p 29%	Zn s 67%		
ecl Zn(3,5-$C_6F_2H_3$)$_2$	Zn s 20%	Zn p 16%				Zn p 36%	Zn p 52%	Zn s 55%		
stag Zn(3,4,5-$C_6F_3H_2$)$_2$	Zn s 21%	Zn p 16%				Zn p 41%	Zn p 42%	Zn s 53%		
ecl Zn(2,3,5,6-$C_6F_4H_1$)$_2$	Zn s 22%	Zn p 17%				Zn p 35%	Zn p 16%	Zn p 47%	Zn s 62%	
stag Zn(2,4,6-$C_6F_3H_2$)$_2$	Zn s 23%	Zn p 17%				Zn p 41%	Zn p 42%			Zn s 67%
stag Zn(4-$C_6F_1H_4$)$_2$	Zn s 21%	Zn p 16%				Zn p 48%	Zn p 50%	Zn s 54%		

Supplementary Table 6. Calculated valence molecular orbitals for organozinc complexes. Valence molecular orbitals with strong Zn 4p (green) and Zn 4s (red) contributions have been highlighted.

	HOMO-32	HOMO-16	HOMO-15	HOMO-14	HOMO-10	HOMO-9	HOMO-8	HOMO-7	HOMO-6	HOMO-5	HOMO-1	HOMO	LUMO	LUMO+1	LUMO+2	LUMO+3
$ZnEt_2$											Zn s 23%	**Zn p 16%**	Zn s 49%	**Zn p 70%**	**Zn p 80%**	
$ZnCl_2(THF)_2$								Zn s 3%<br>Zn p 6%	**Zn p 9%**	Zn p 2%			Zn s 25%<br>Zn p 22%	Zn p 27%	**Zn p 46%**	**Zn p 48%**
$[Zn(NCMe)_6]^{2+}$	Zn s 3%	**Zn p 7%**	**Zn p 7%**	**Zn p 7%**									Zn s 29%	**Zn p 35%**	**Zn p 36%**	**Zn p 36%**
$[Zn(OH_2)_6]^{2+}$				Zn 4s 10%	**Zn 4p 7%**	**Zn 4p 6%**	**Zn 4p 6%**						Zn s 19%	**Zn p 61%**	**Zn p 61%**	**Zn 4p 65%**

Supplementary Table 7. Electronic structure descriptors for ZnR_2 and common zinc-containing species: calculated *E*(Zn 1s) energy, *E*(OMO,Zn p), *E*(UMO,Zn p), Zn-specific hardness (μ_{Zn}), Zn-specific absolute electronegativity (χ_{Zn}), Global electrophilicity index (ω_{Zn}), Taft inductive substituent constant (I_R), and experimental R-VtC-XES energy gap, *E*(gap, exp).

Sample	PCM	*E*(Zn 1s) / eV	*E*(OMO,Zn p) / eV	*E*(UMO,Zn p) / eV	Zinc-specific hardness, η_{Zn} / eV	Zinc-specific absolute electronegativity, χ_{Zn} / eV	Global electrophilicity index, ω_{zn} / eV	Taft constant, I_R / kcal mol^{-1}	R-VtC-XES energy gap[c], *E*(gap,exp) / eV
$ZnMe_2$	Toluene	9674.62	9.37	-1.75	11.12	3.81	0.65	0.0 [a]	6.02
$ZnEt_2$	Toluene	9674.52	8.71	-1.63	10.33	3.54	0.61	-0.10 [a]	5.30
Zn(*i*-Pr)$_2$	Toluene	9674.50	8.29	-1.61	9.90	3.34	0.56	-0.19 [a]	4.92
Zn(*n*-Pr)$_2$	Toluene	9674.53	8.71	-1.61	10.32	3.55	0.61	0.10 [a]	
Zn(*n*-Bu$_{2)}$	Toluene	9674.53	8.67	-1.62	10.29	3.52	0.60		
Zn(*t*-Bu)$_2$	Toluene	9674.53	8.05	-1.75	9.80	3.15	0.51	-0.30 [a]	
Staggered $ZnPh_2$	Toluene	9675.02	9.58	-1.17	10.76	4.20	0.82	0.60 [a]	5.54
Eclipsed $ZnPh_2$	Toluene	9675.02	9.58	-1.01	10.59	4.29	0.87		
Staggered $Zn(C_6F_5)_2$	Toluene	9676.15	11.66	-0.58	12.24	5.54	1.25		7.01
Eclipsed $Zn(C_6F_5)_2$	Toluene	9676.15	11.65	-0.26	11.91	5.69	1.36		
Zn(4-$C_6F_1H_4$)$_2$	Toluene	9675.16	9.90	-1.20	11.10	4.45	0.85		
Zn(2,6-$C_6F_2H_3$)$_2$	Toluene	9675.57	10.85	-0.75	11.60	5.05	1.10		
Zn(3,5-$C_6F_2H_3$)$_2$	Toluene	9675.48	10.24	-0.61	10.85	4.82	1.07		
Zn(3,4,5-$C_6F_3H_2$)$_2$	Toluene	9675.59	10.46	-0.86	11.32	4.80	1.02		
Zn(2,4,6-$C_6F_3H_2$)$_2$	Toluene	9675.71	11.14	-0.74	11.88	5.20	1.14		
Zn(2,3,5,6-$C_6F_4H_1$)$_2$	Toluene	9676.02	11.45	-0.26	11.71	5.60	1.34		
$ZnEt_2$	Hexane	9674.61	8.75	1.58	7.17	5.17	1.86		
$[Zn(NCMe)_6]^{2+}$	MeCN	9675.29	15.53	1.75	13.78	8.64	2.71		13.23
$[Zn(OH_2)_6]^{2+}$	Water	9675.09	15.67	1.75	13.92	8.71	2.73		16.06
$ZnCl_2(THF)_2$	THF	9674.94	11.30	-2.30	13.60	4.50	0.74		10.04
$[ZnCl_4]^{2-}$	Ionic Liquid[b]	9673.96	10.69	-3.32	14.01	3.69	0.48		10.18

[a] Taken from reference [1]. [b] Dielectric constant of 11.40. [c] Obtained by fitting a gaussian peak to R-VtC-XE spectra and shifting by the elastic shift in Supplementary Table 8.

Supplementary Table 8. Experimental fitting of the R-VtC-XE spectra for ZnR_2 and zinc-containing species.

Compound	Scan type	Scan	Incident energy / eV	Fitting type	Elastic peak / eV	Difference / eV	Applied shift NR-VtC-XES / eV	Applied shift R-VtC-XES / eV[a]
0.1 M $ZnMe_2$ in toluene	RXES	207495	9661.40	Gauss	9661.46	-0.06	-0.06	0.06
0.1 M $ZnEt_2$ in toluene	RIXS map	164772	9661.10	Gauss	9660.73	0.37	0.37	-0.37
0.1 M Zn(*i*-Pr)$_2$ in toluene	RXES	183901	9661.00	Gauss	9660.56	0.44	0.44	-0.44
0.1 M $ZnPh_2$ in toluene	RXES	183933	9661.00	Gauss	9660.63	0.37	0.37	-0.37
0.033 M $Zn(C_6F_5)_2$ in toluene	RXES	184090	9661.50	Gauss	9661.15	0.35	0.35	-0.35
0.1 M $ZnCl_2$ in THF ($ZnCl_2(THF)_2$)	RXES	234217	9663.60	Gauss	9665.26	-1.66	-1.66	1.66
0.1 M $Zn(OTf)_2$ in MeCN ($[Zn(NCMe)_6]^{2+}$)	RXES	184237	9663.40	Gauss	9662.93	0.47	0.47	-0.47
0.1 M $ZnCl_2$ in water ($[Zn(OH_2)_6]^{2+}$)	RIXS map	164936	9665.80	Gauss	9665.43	0.37	0.37	-0.37
x = 0.33 $ZnCl_2$ in omimCl ($[ZnCl_4]^{2-}$)	RIXS map	142503	9663.80	Gauss	9663.90	-0.01	-0.01	0.01

[a] R-VtC-XES shifts are inverted to align with energy transfer plotting conventions.

Supplementary Figures

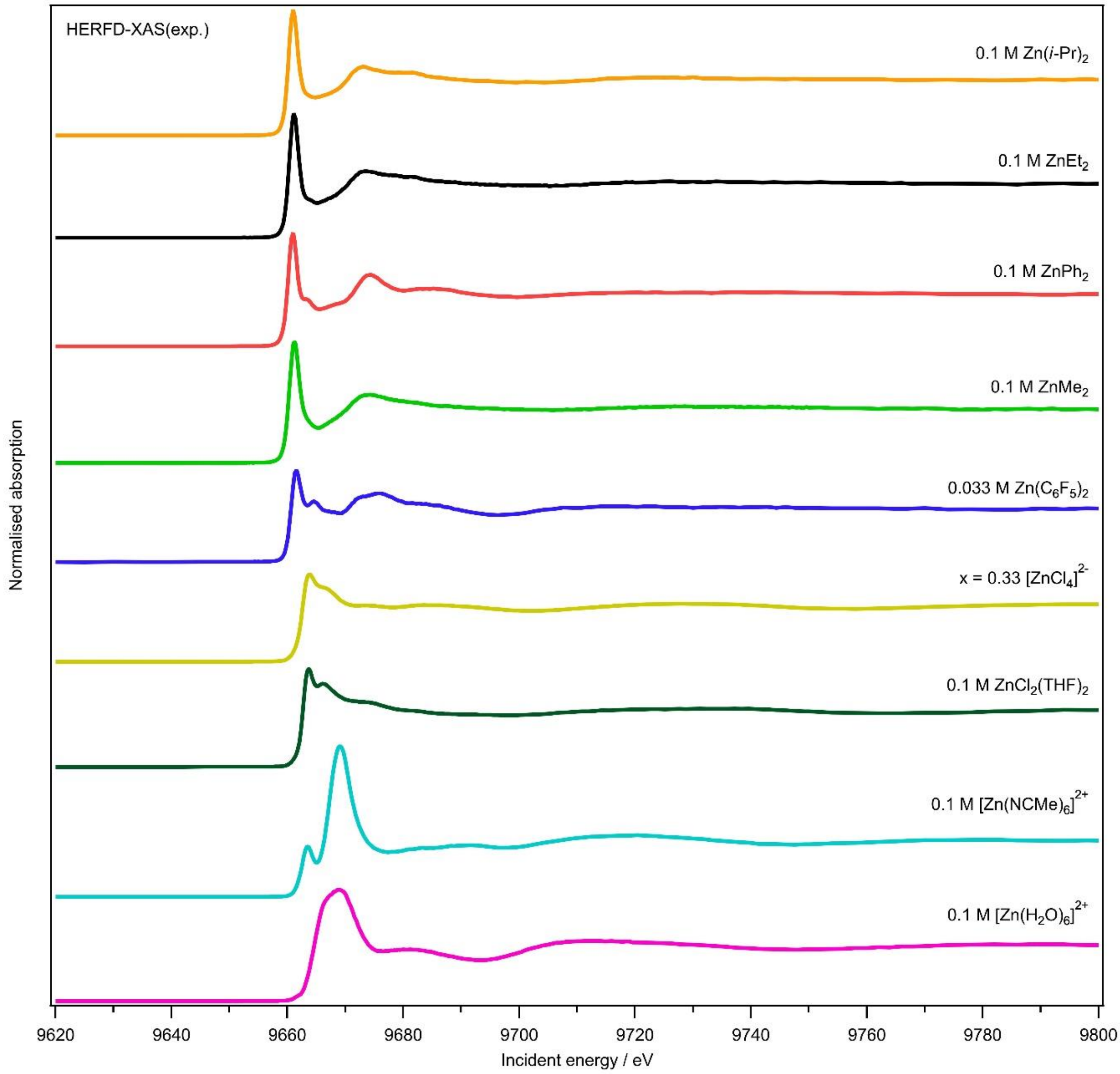

Supplementary Figure 1. Zn 1s HERFD-XAS (y intensities normalised) spectra of organozinc compounds ZnR_2 (R = Me, Et, *i*-Pr, Ph, C_6F_5, concentration 0.1 M for all apart from C_6F_5 which was 0.033 M), $[ZnCl_4]^{2-}$ (*x* = 0.33 $ZnCl_2$ in $[C_8C_1Im]Cl$), $ZnCl_2(THF)_2$ (0.1 M $ZnCl_2$ in THF), $[Zn(NCMe)_6]^{2+}$ (0.1 M $Zn(OTf)_2$ in MeCN) and $[Zn(OH_2)_6]^{2+}$ (0.1 M $ZnCl_2$ in H_2O).

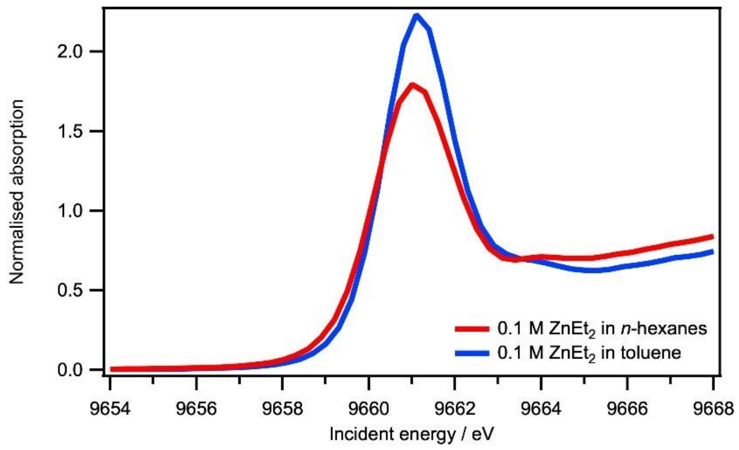

Supplementary Figure 2. Zn 1s XA spectra (y-intensities normalised) of 0.1 M $ZnEt_2$ in *n*-hexanes and toluene.

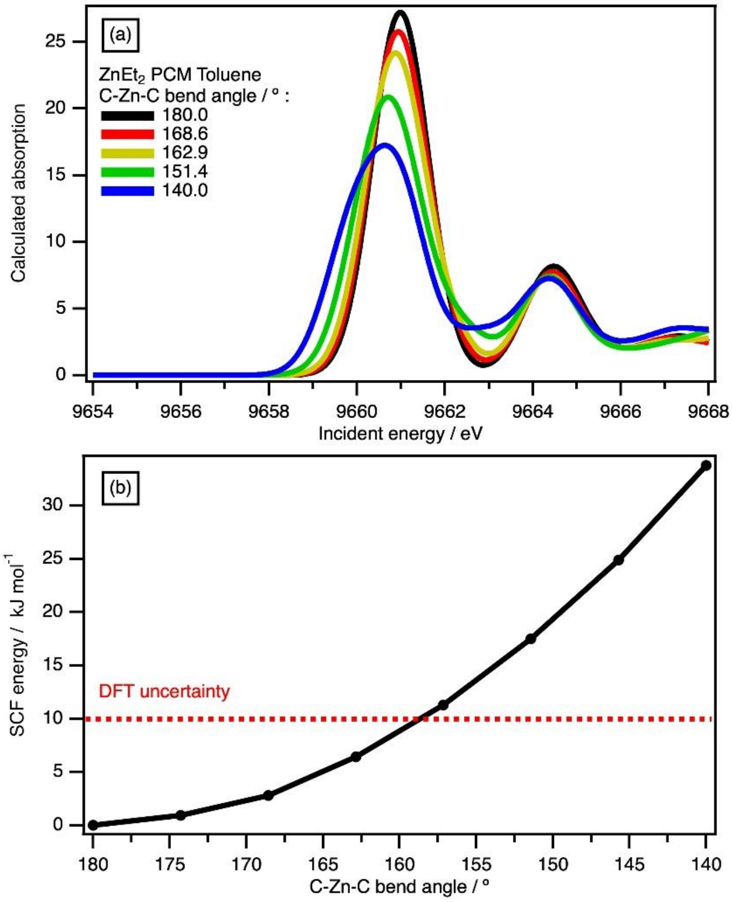

Supplementary Figure 3. (a) Calculated Zn 1s XA spectra of $ZnEt_2$ (PCM toluene) at different bend angles (FWHM: 2.5 eV, applied shift of -8.90 eV); (b) calculated SCF energy as a function of $ZnEt_2$ bend angle. The red line highlights the error of the DFT uncertainty.

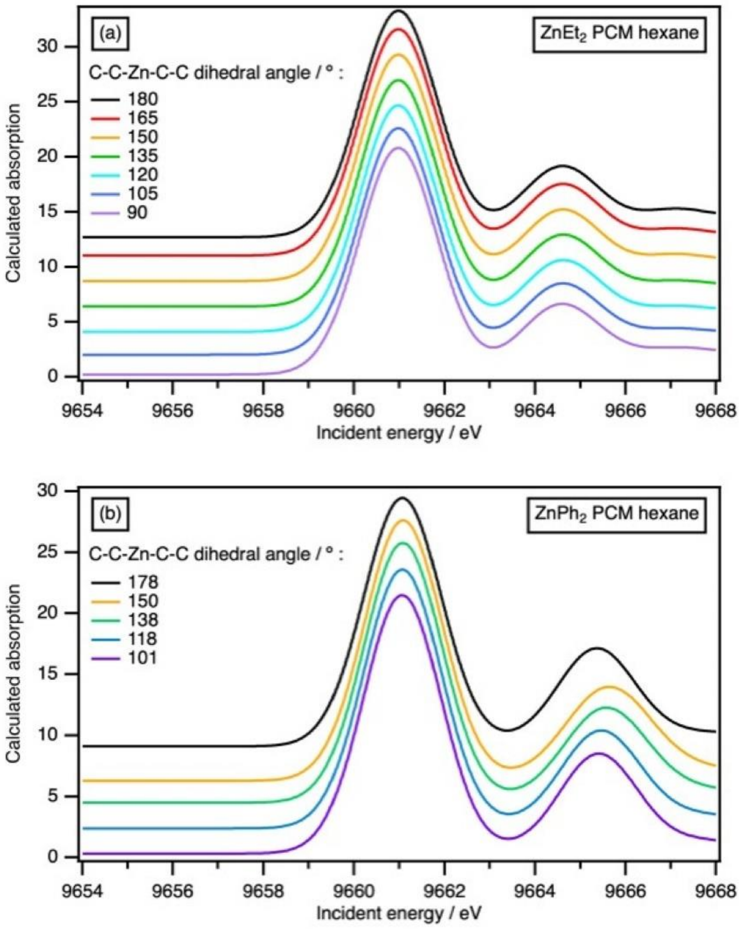

Supplementary Figure 4. Calculated Zn 1s XA spectra as a function of dihedral angle: (a) $ZnEt_2$ (PCM hexane, FWHM 2.5 eV, applied shift of -8.90 eV), (b) $ZnPh_2$ (PCM hexane, FWHM 2.5 eV, applied shift of -8.90 eV).

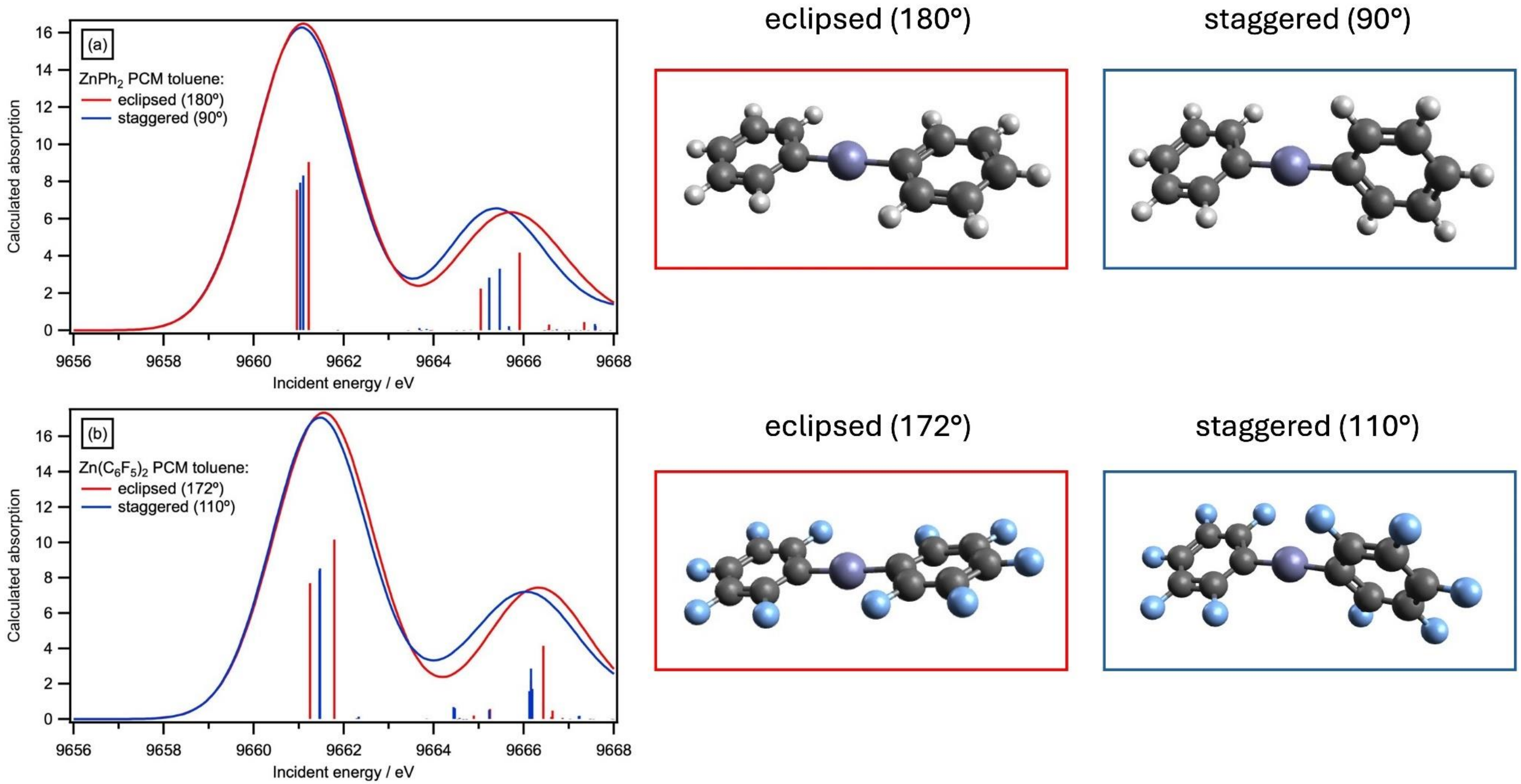

Supplementary Figure 5. Calculated Zn 1s XA spectra of staggered and eclipsed conformers of (a) $ZnPh_2$ (PCM toluene, FWHM 2.5 eV, applied shift of -8.9 eV) and (b) $Zn(C_6F_5)_2$ (PCM toluene, FWHM 2.5 eV, applied shift of -8.90 eV).

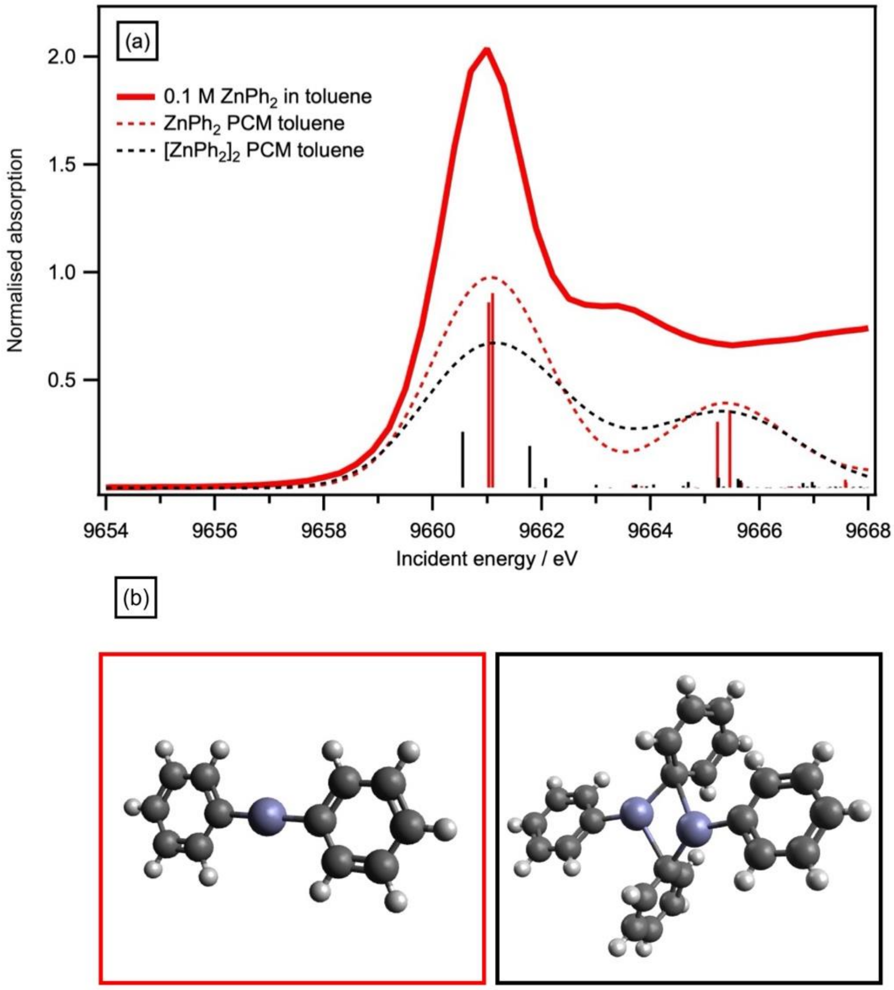

Supplementary Figure 6. (a) Zn 1s XA spectra of 0.1 M $ZnPh_2$ in toluene, calculated $ZnPh_2$ and $(ZnPh_2)_2$ (PCM toluene, FWHM 2.5 eV, applied shift of -8.90 eV; (b) Calculated structures for $ZnPh_2$ (left, red box) and $(ZnPh_2)_2$ (right, black box). Coordinates for $(ZnPh_2)_2$ were taken from the solid-state $(ZnPh_2)_2$ structure in reference [2].

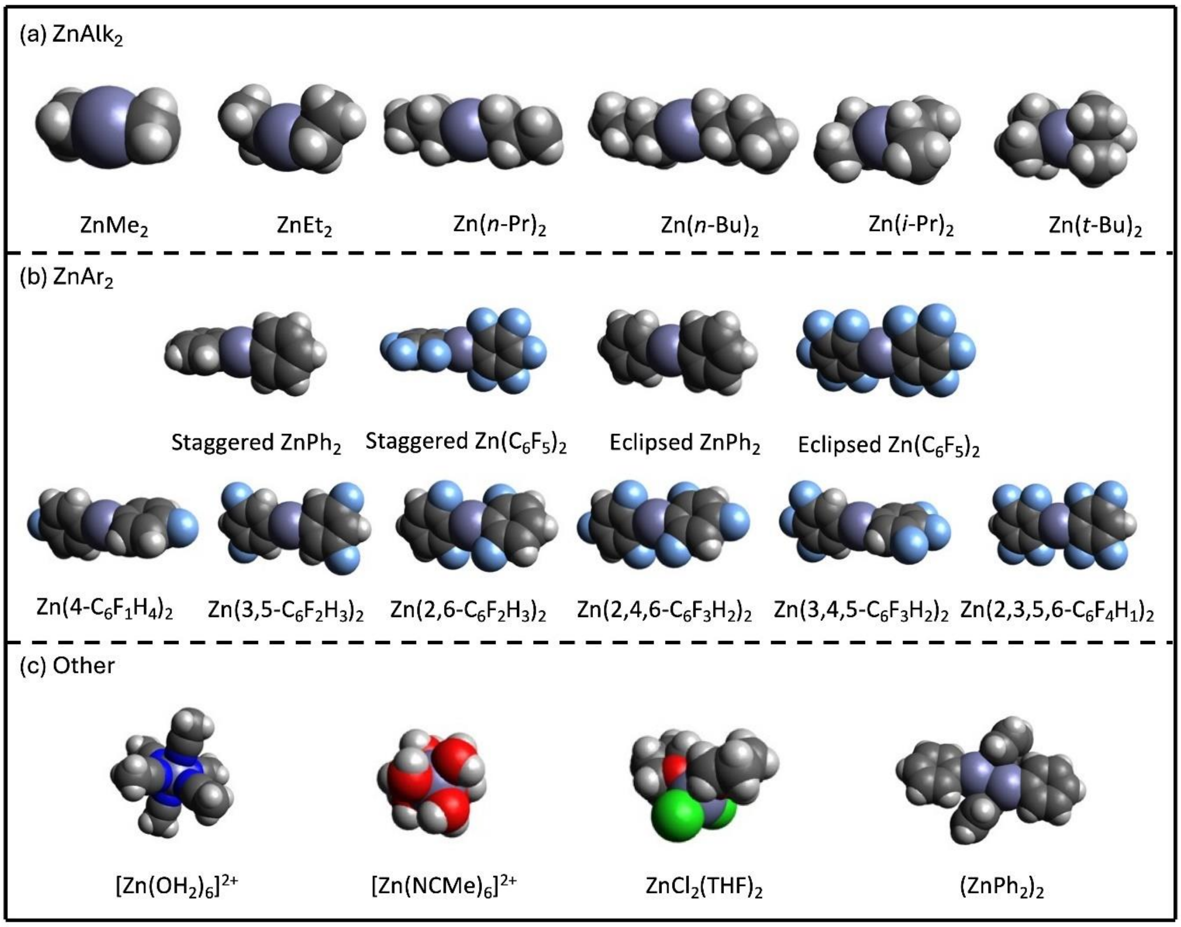

Supplementary Figure 7. Calculated ZnR_2 structures with a van der Waals space-filling model applied.

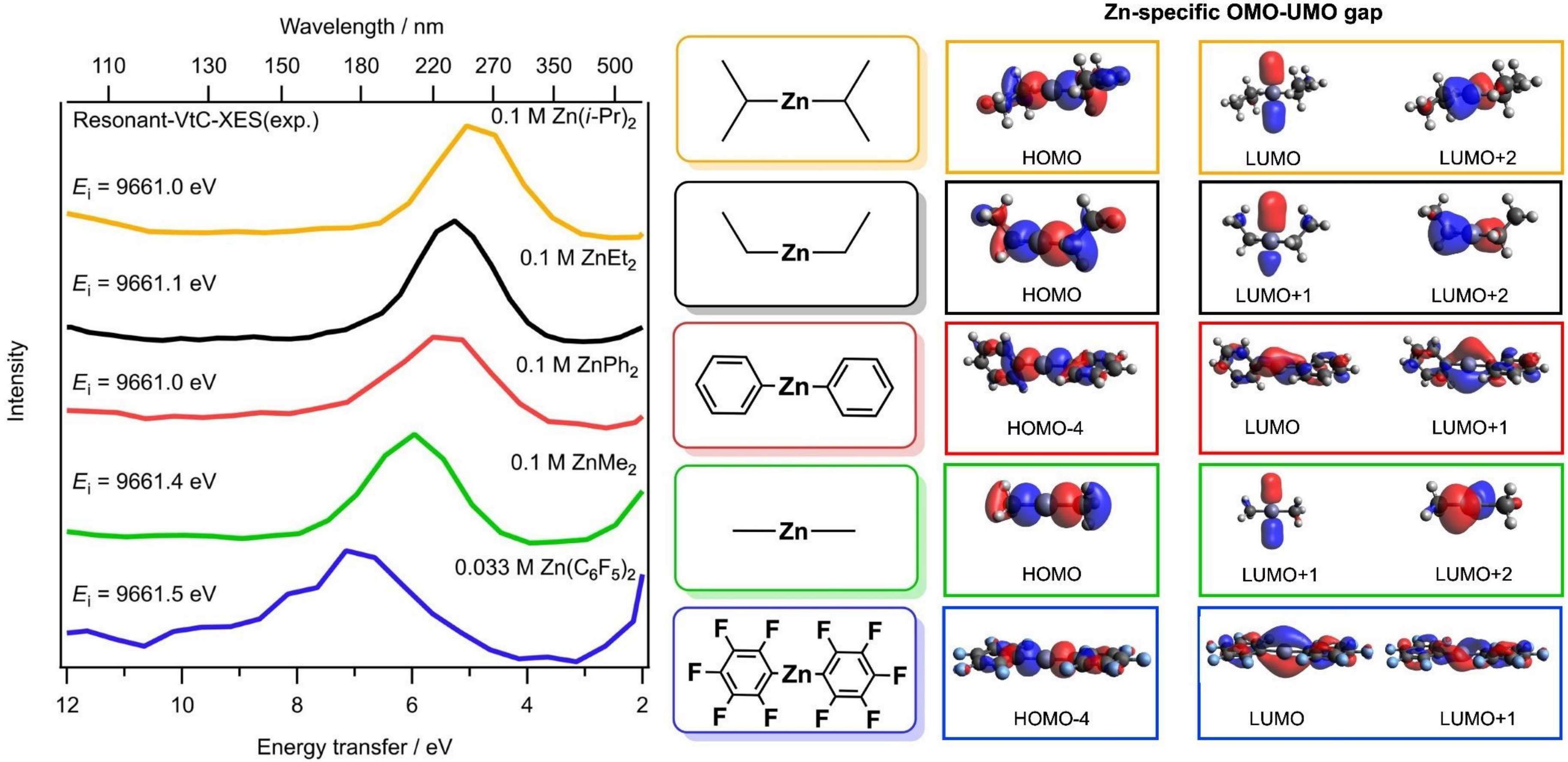

Supplementary Figure. 8. Left: Experimental R-VtC-XE spectra for ZnR_2 (R = Me, Et, *i*-Pr, Ph, C_6F_5, concentration 0.1 M for all apart from C_6F_5 which was 0.033 M). Right: Visual representations of key Zn p-based OMOs and UMOs; for ZnR_2 the UMO shown here is degenerate (or near-degenerate).

$ZnMe_2$ PCM toluene

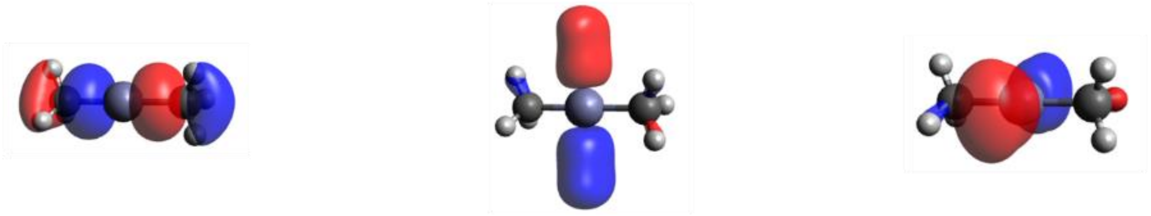

HOMO	LUMO+1	LUMO+2
Zn 18.4% of which 18.4% is Zn4p	Zn 86.1% of which 86.1% is Zn4p	Zn 86.1% of which 86.1% is Zn4p
C: 57.6%	C: 6.2%	C: 6.2%
H: 17.9%	H: 3.4%	H: 3.4%
Transition energy: 9665.25 eV	Transition energy: 9670.06 eV	Transition energy: 9670.06 eV
Orbital energy: 9.37 eV	Orbital energy: -1.75 eV	Orbital energy: -1.75 eV

Supplementary Figure 9. Calculated molecular orbitals of $ZnMe_2$. The percentage of Zn 4p character has been listed for all key orbitals involved in both XAS and XES alongside the calculated transition energy (from KS-DFT XES (OMOs) and TDDFT (UMOs)) and orbital energy.

ZnEt$_2$ PCM toluene

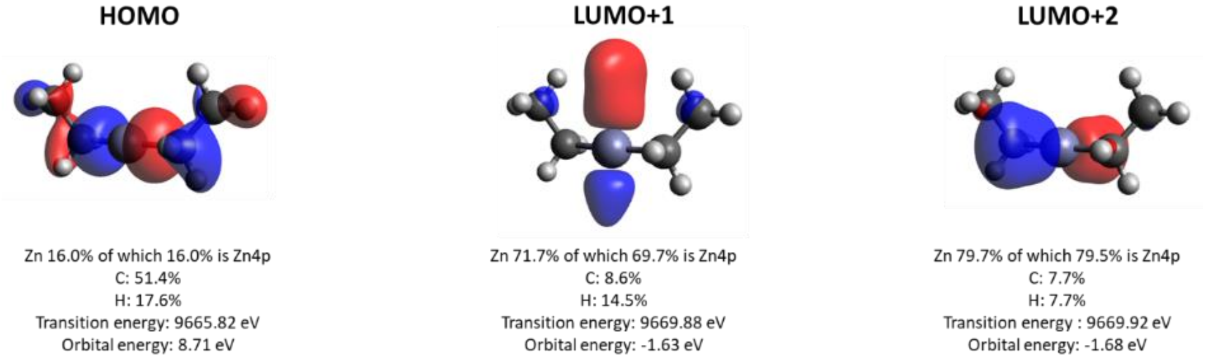

Supplementary Figure 10. Calculated molecular orbitals of ZnEt$_2$. The percentage of Zn 4p character has been listed for all key orbitals involved in both XAS and XES alongside the calculated transition energy (from KS-DFT XES (OMOs) and TDDFT (UMOs)) and orbital energy.

Zn(*n*-Pr)$_2$ PCM toluene

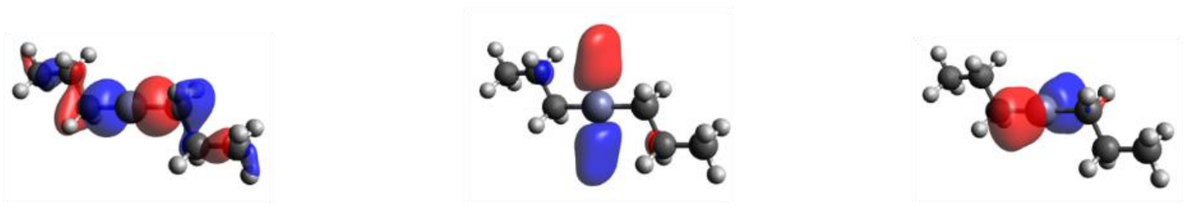

HOMO

Zn 15.9% of which 15.9% is Zn4p
C: 55.7%
H: 13.9%
Transition energy: 9665.82 eV
Orbital energy: 8.71 eV

LUMO+1

Zn 75.8% of which 75.8% is Zn4p
C: 10.0%
H: 8.9%
Transition energy: 9669.86 eV
Orbital energy: -1.61 eV

LUMO+2

Zn 80.9% of which 80.8% is Zn4p
C: 8.2%
H: 6.6%
Transition energy: 9669.95 eV
Orbital energy: -1.69 eV

Supplementary Figure 11. Calculated molecular orbitals of Zn(*n*-Pr)$_2$. The percentage of Zn 4p character has been listed for all key orbitals involved in both XAS and XES alongside the calculated transition energy (from KS-DFT XES (OMOs) and TDDFT (UMOs)) and orbital energy.

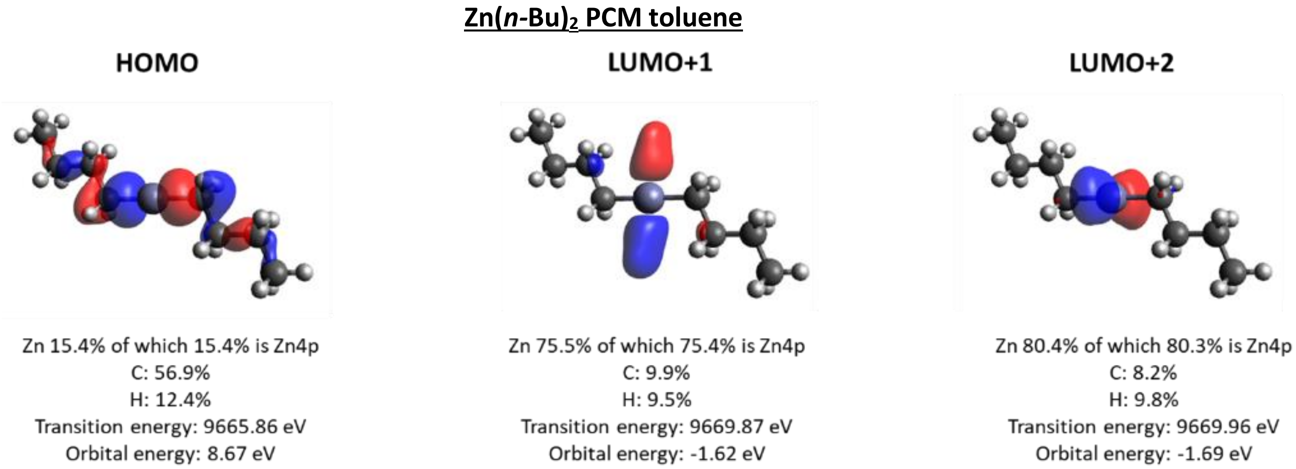

Supplementary Figure 12. Calculated molecular orbitals of Zn(*n*-Bu)$_2$. The percentage of Zn 4p character has been listed for all key orbitals involved in both XAS and XES alongside the calculated transition energy (from KS-DFT XES (OMOs) and TDDFT (UMOs)) and orbital energy.

Zn(*i*-Pr)$_2$ PCM toluene

HOMO LUMO LUMO+2

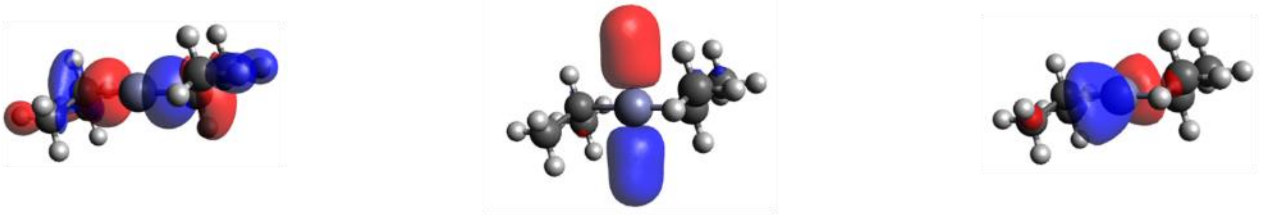

HOMO: Zn 14.8% of which 14.8% is Zn4p
C: 48.5%
H: 15.4%
Transition energy: 9666.21 eV
Orbital energy: 8.29 eV

LUMO: Zn 75.2% of which 75.2% is Zn4p
C: 8.8%
H: 10.9%
Transition energy: 9669.81 eV
Orbital energy: -1.61 eV

LUMO+2: Zn 68.1% of which 68.1% is Zn4p
C: 11.4%
H: 15.9%
Transition energy: 9669.88 eV
Orbital energy: 1.71 eV

Supplementary Figure 13. Calculated molecular orbitals of Zn(*i*-Pr)$_2$. The percentage of Zn 4p character has been listed for all key orbitals involved in both XAS and XES alongside the calculated transition energy (from KS-DFT XES (OMOs) and TDDFT (UMOs)) and orbital energy.

Zn(t-Bu)$_2$ PCM toluene

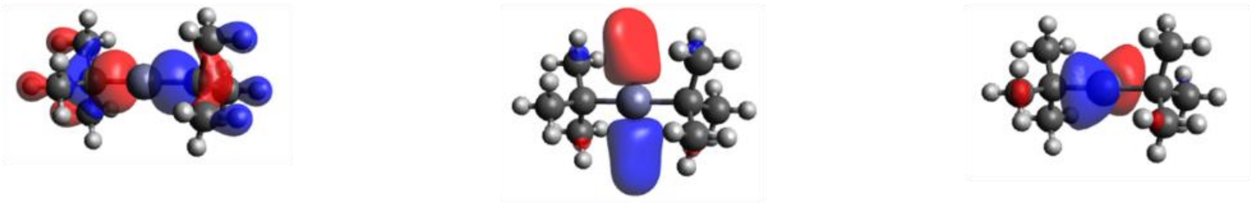

HOMO	LUMO+1	LUMO+2
Zn 14.1% of which 14.1% is Zn4p	Zn 65.9% of which 65.7% is Zn4p	Zn 66.0% of which 65.9% is Zn4p
C: 48.0%	C: 11.8%	C:12.0 %
H: 13.1%	H: 16.3%	H: 16.4%
Transition energy: 9666.48 eV	Transition energy: 9669.89 eV	Transition energy: 9669.90 eV
Orbital energy: 8.05 eV	Orbital energy: -1.75 eV	Orbital energy: -1.75 eV

Supplementary Figure 14. Calculated molecular orbitals of Zn(t-Bu)$_2$. The percentage of Zn 4p character has been listed for all key orbitals involved in both XAS and XES alongside the calculated transition energy (from KS-DFT XES (OMOs) and TDDFT (UMOs)) and orbital energy.

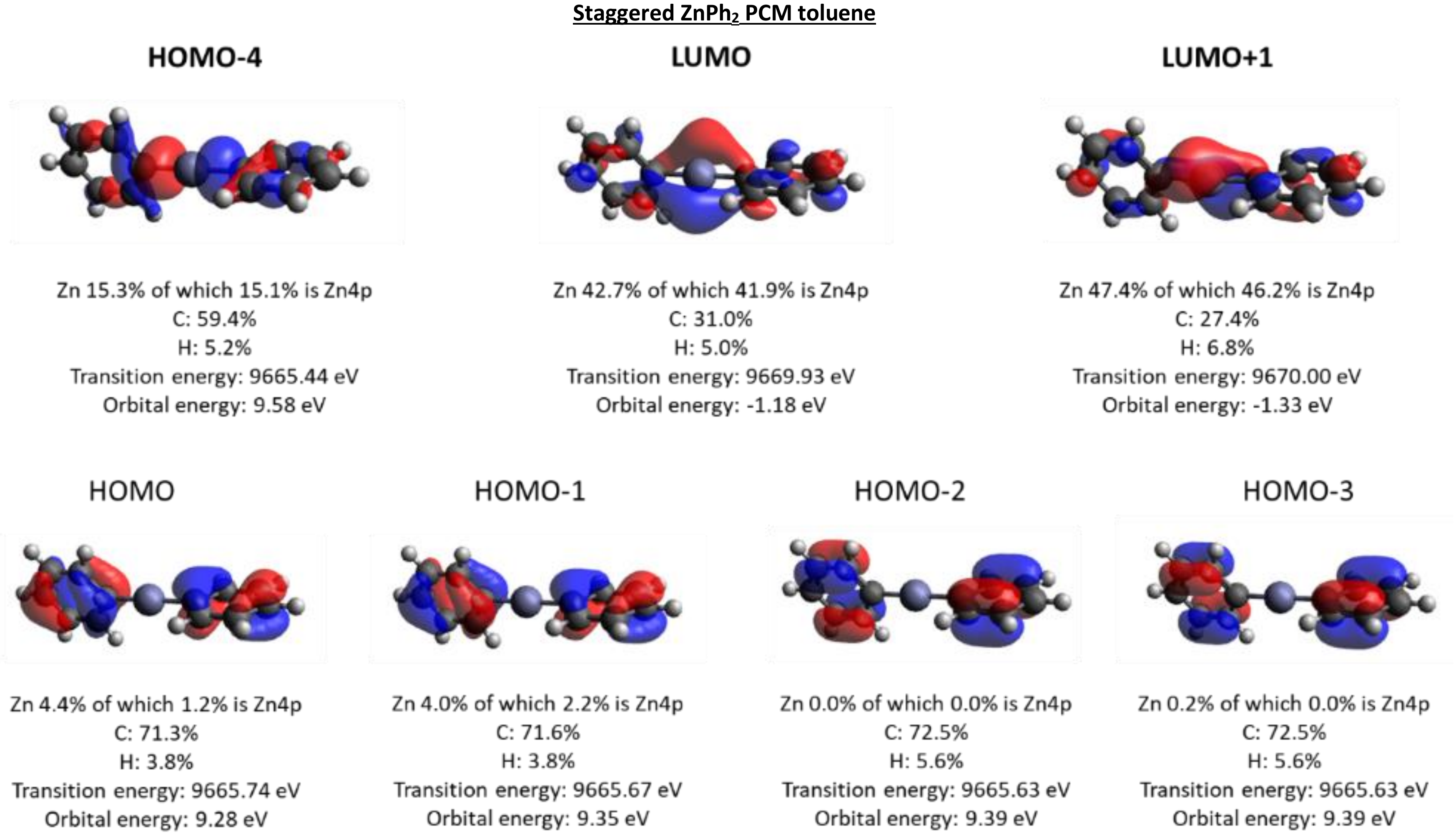

Supplementary Figure 15. Calculated molecular orbitals of staggered $ZnPh_2$. The percentage of Zn 4p character has been listed for all key orbitals involved in both XAS and XES alongside the calculated transition energy (from KS-DFT XES (OMOs) and TDDFT (UMOs)) and orbital energy.

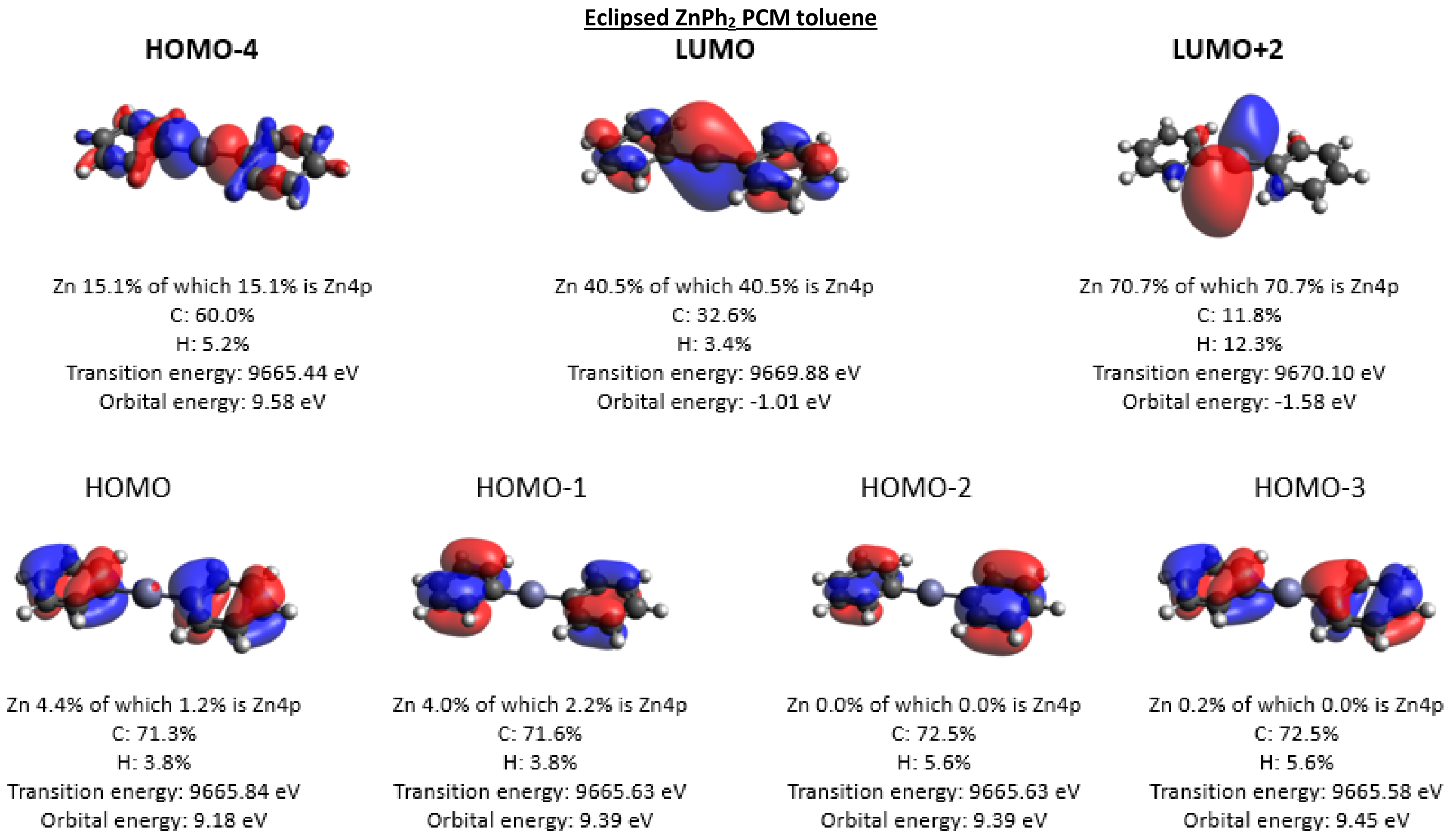

Supplementary Figure 16. Calculated molecular orbitals of eclipsed $ZnPh_2$. The percentage of Zn 4p character has been listed for all key orbitals involved in both XAS and XES alongside the calculated transition energy (from KS-DFT XES (OMOs) and TDDFT (UMOs)) and orbital energy.

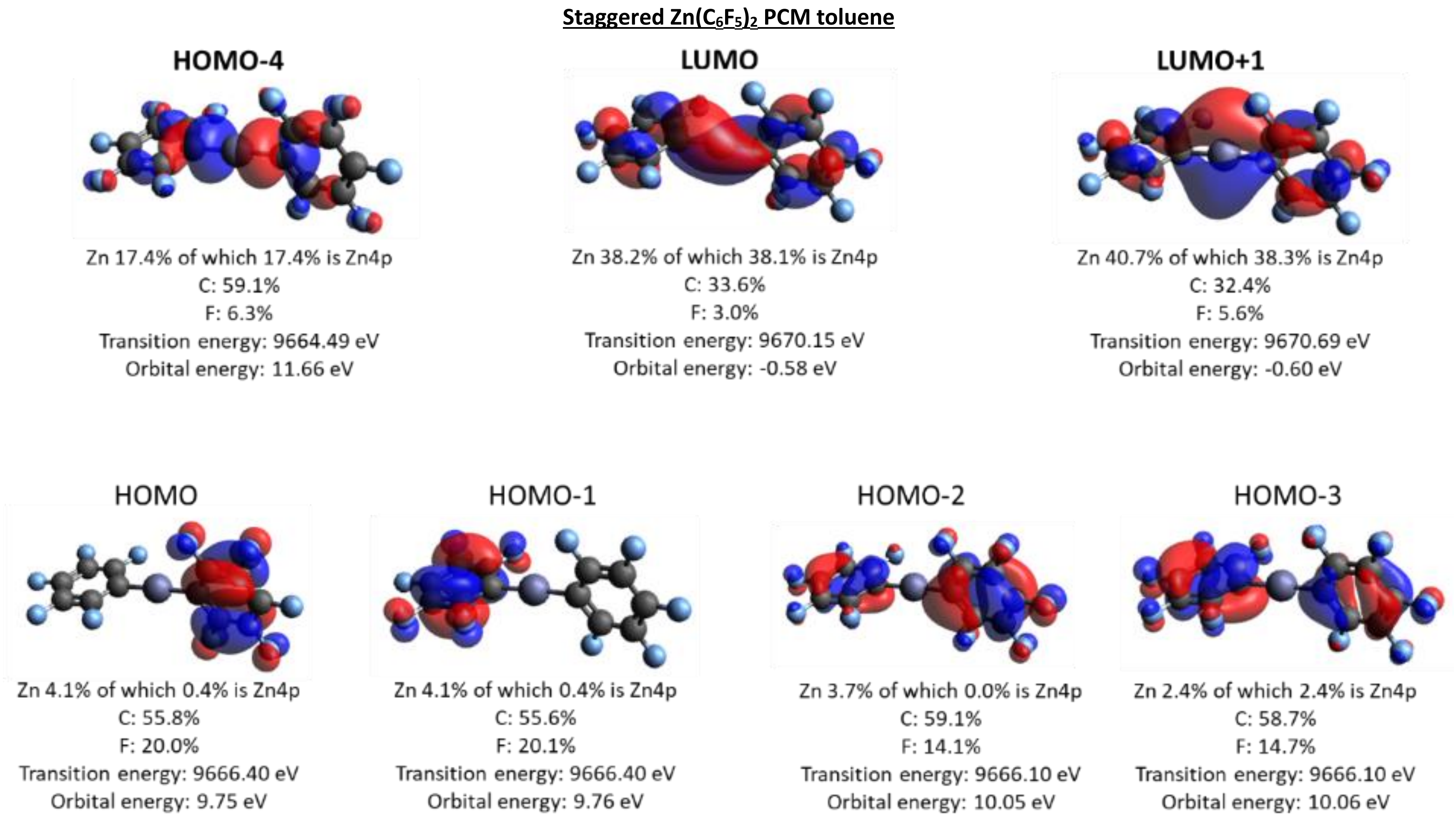

Supplementary Figure 17. Calculated molecular orbitals of staggered $Zn(C_6F_5)_2$. The percentage of Zn 4p character has been listed for all key orbitals involved in both XAS and XES alongside the calculated transition energy (from KS-DFT XES (OMOs) and TDDFT (UMOs)) and orbital energy.

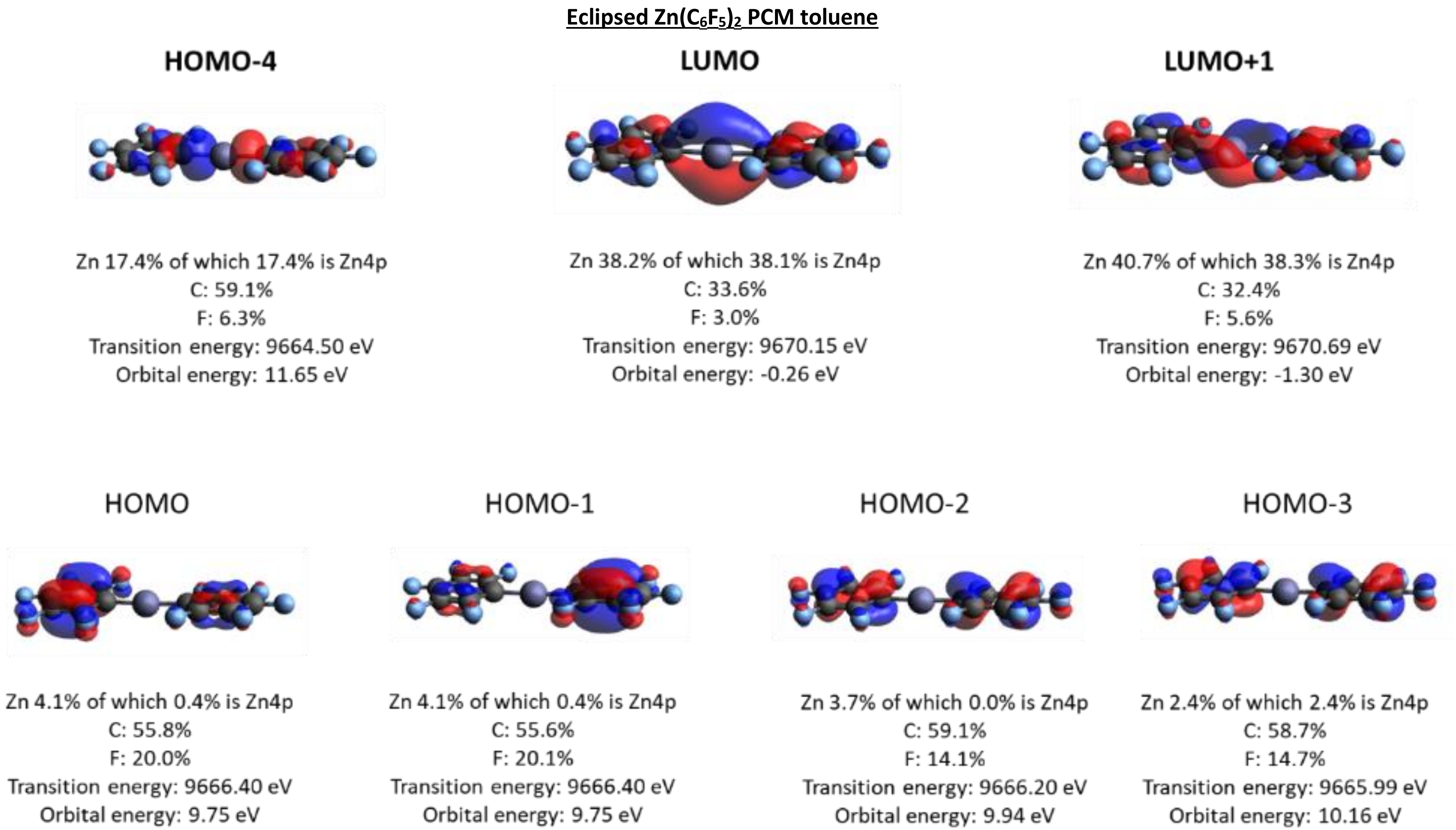

Supplementary Figure 18. Calculated molecular orbitals of eclipsed $Zn(C_6F_5)_2$. The percentage of Zn 4p character has been listed for all key orbitals involved in both XAS and XES alongside the calculated transition energy (from KS-DFT XES (OMOs) and TDDFT (UMOs)) and orbital energy.

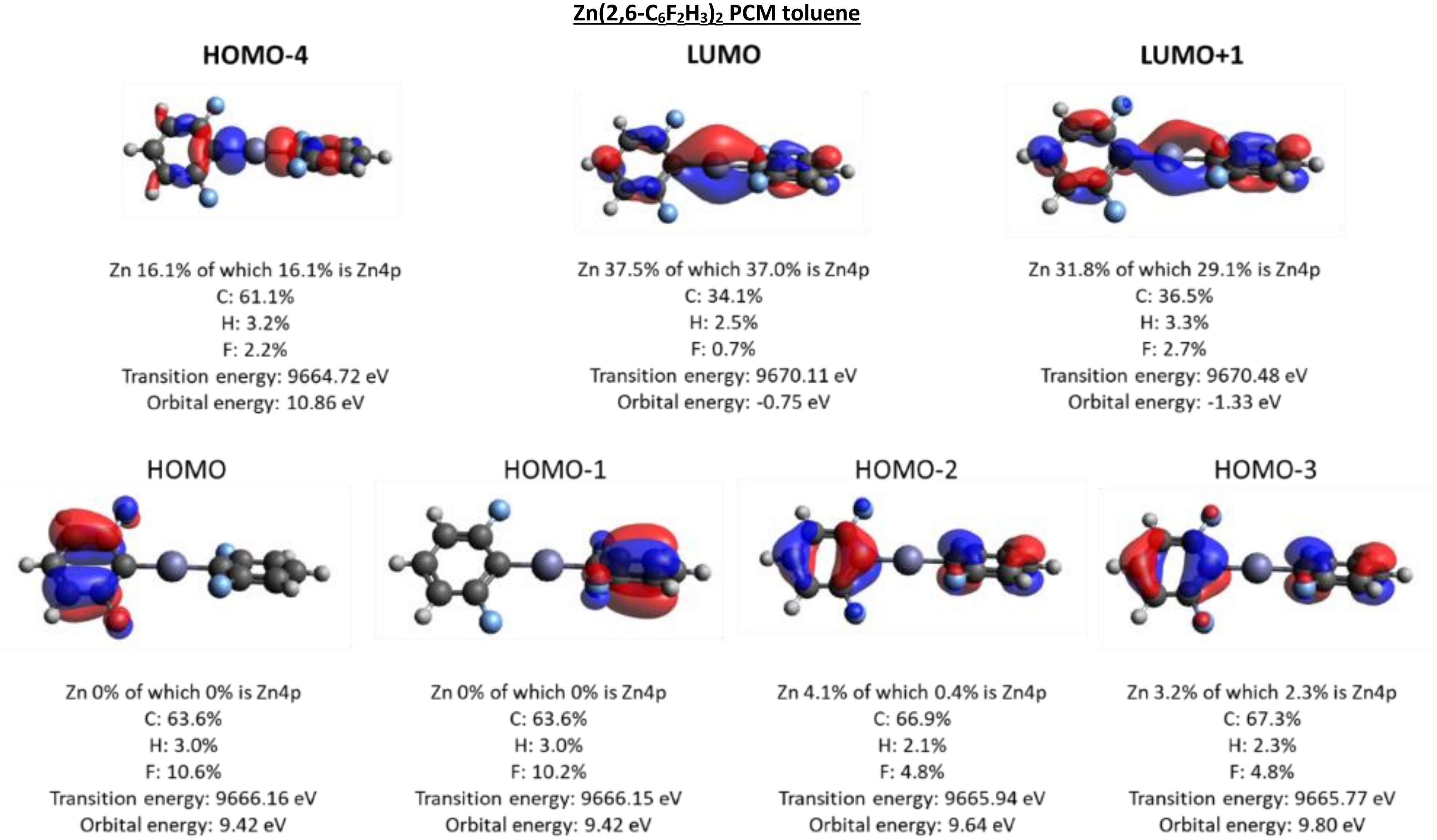

Supplementary Figure 19. Calculated molecular orbitals of eclipsed $Zn(2,6\text{-}C_6F_2H_3)_2$. The percentage of Zn 4p character has been listed for all key orbitals involved in both XAS and XES alongside the calculated transition energy (from KS-DFT XES (OMOs) and TDDFT (UMOs)) and orbital energy.

Zn(3,5-$C_6F_2H_3$)$_2$ PCM toluene

HOMO-4

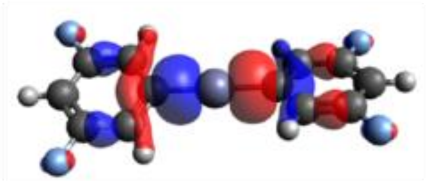

Zn 15.7% of which 15.7% is Zn4p
C: 58.3%
H: 2.8%
F: 3.2%
Transition energy: 9665.25 eV
Orbital energy: 10.14 eV

LUMO

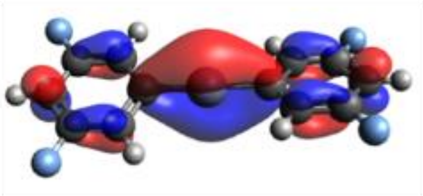

Zn 36.1% of which 36.0% is Zn4p
C: 34.3%
H: 3.2%
F: 0.4%
Transition energy: 9669.89 eV
Orbital energy: -0.61 eV

LUMO+1

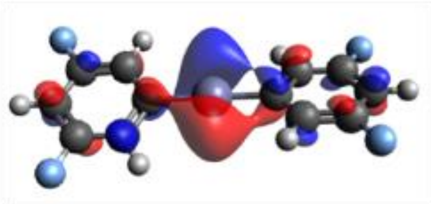

Zn 53.8% of which 52.2% is Zn4p
C: 22.9%
H: 8.6%
F: 0.4%
Transition energy: 9670.24 eV
Orbital energy: -1.32 eV

HOMO

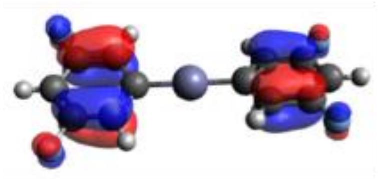

Zn 0% of which 0% is Zn4p
C: 63.5%
H: 3.0%
F: 10.5%
Transition energy: 9666.01 eV
Orbital energy: 9.47 eV

HOMO-1

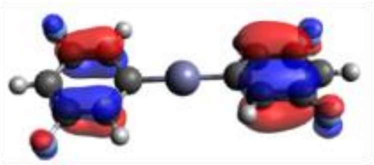

Zn 0.3% of which 0% is Zn4p
C: 63.4%
H: 2.9%
F: 10.6%
Transition energy: 9666.01 eV
Orbital energy: 9.47 eV

HOMO-2

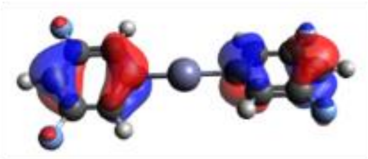

Zn 4.1% of which 0.1% is Zn4p
C: 67.3%
H: 2.9%
F: 3.6%
Transition energy: 9665.80 eV
Orbital energy: 9.69 eV

HOMO-3

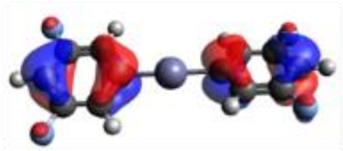

Zn 2.9% of which 2.7% is Zn4p
C: 67.5%
H: 2.7%
F: 4.4%
Transition energy: 9665.59 eV
Orbital energy: 9.89 eV

Supplementary Figure 20. Calculated molecular orbitals of eclipsed Zn(3,5-$C_6F_2H_3$)$_2$. The percentage of Zn 4p character has been listed for all key orbitals involved in both XAS and XES alongside the calculated transition energy (from KS-DFT XES (OMOs) and TDDFT (UMOs)) and orbital energy.

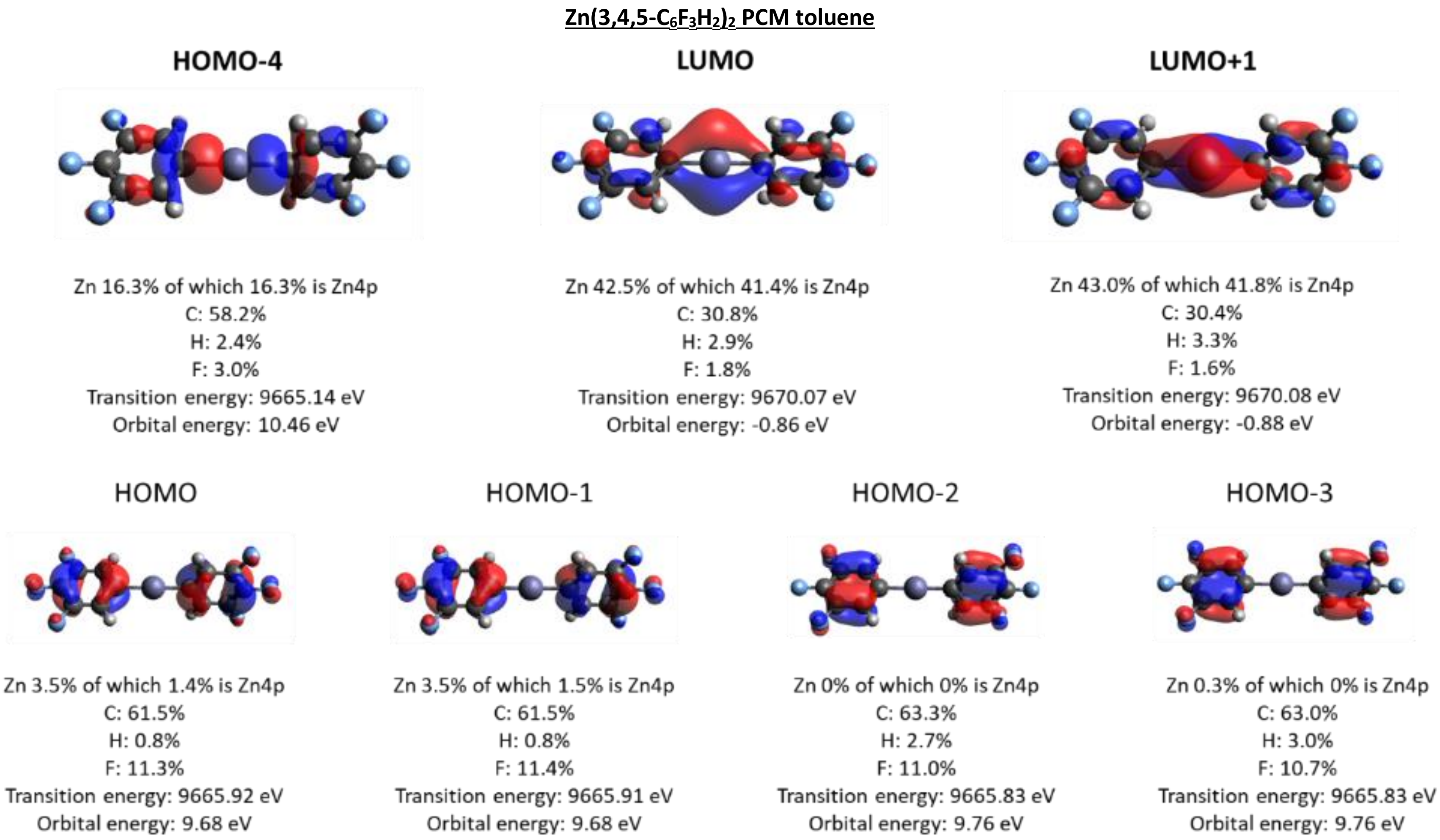

Supplementary Figure 21. Calculated molecular orbitals of eclipsed $Zn(3,4,5\text{-}C_6F_3H_2)_2$. The percentage of Zn 4p character has been listed for all key orbitals involved in both XAS and XES alongside the calculated transition energy (from KS-DFT XES (OMOs) and TDDFT (UMOs)) and orbital energy.

Zn(2,3,5,6-$C_6F_4H_1$)$_2$ PCM toluene

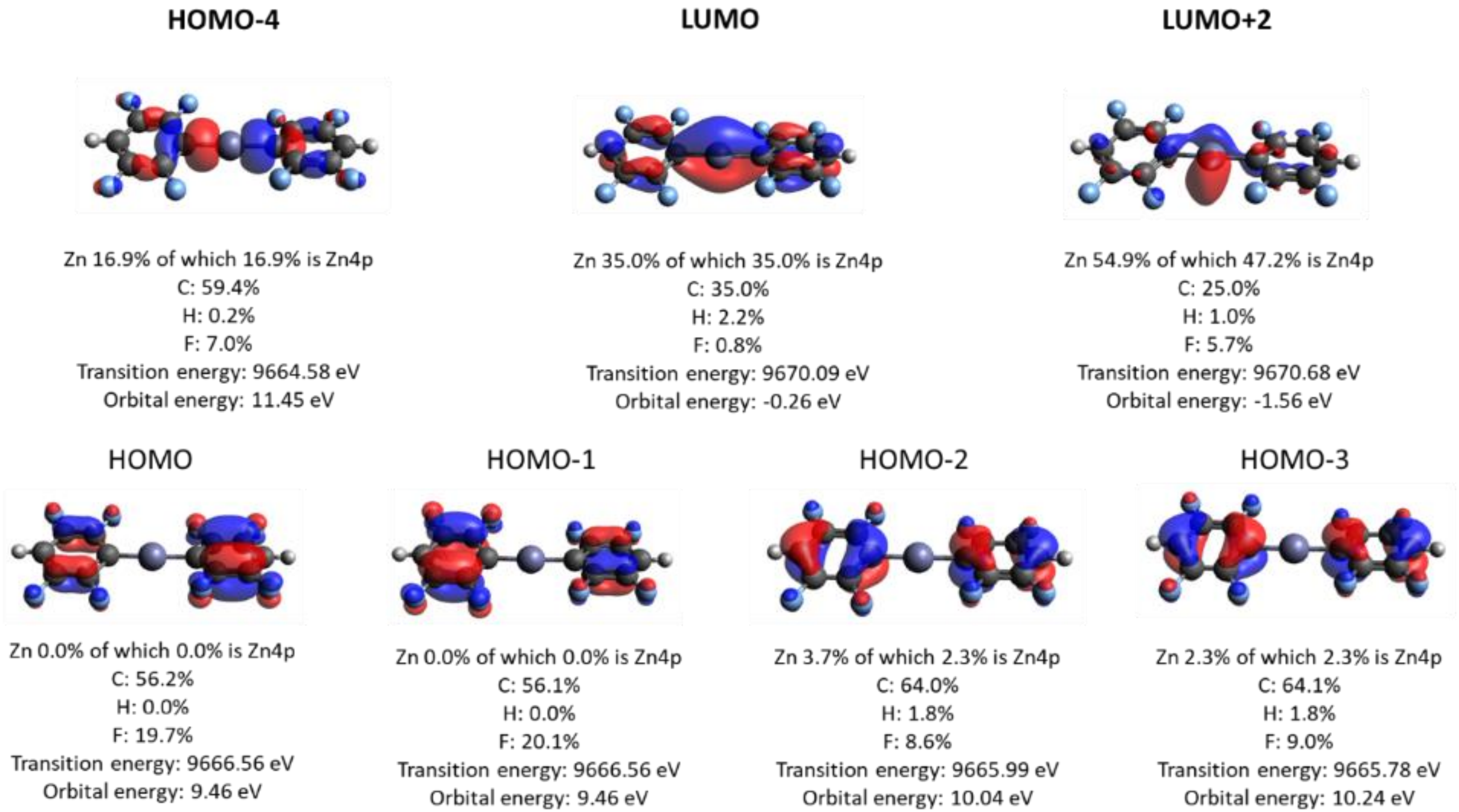

Supplementary Figure 22. Calculated molecular orbitals of eclipsed Zn(2,3,5,6-$C_6F_4H_1$)$_2$. The percentage of Zn 4p character has been listed for all key orbitals involved in both XAS and XES alongside the calculated transition energy (from KS-DFT XES (OMOs) and TDDFT (UMOs)) and orbital energy.

Zn(2,4,6-$C_6F_3H_2$)$_2$ PCM toluene

HOMO-4

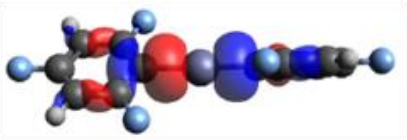

Zn 17.1% of which 17.1% is Zn4p
C: 62.1%
H: 1.6%
F: 2.0%
Transition energy: 9664.57 eV
Orbital energy: 11.14 eV

LUMO

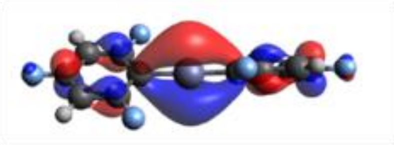

Zn 41.4% of which 41.0% is Zn4p
C: 32.4%
H: 0.4%
F: 2.3%
Transition energy: 9670.17 eV
Orbital energy: -0.74 eV

LUMO+1

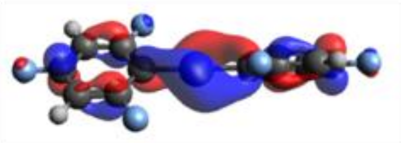

Zn 40.9% of which 38.7% is Zn4p
C: 32.1%
H: 0.7%
F: 4.7%
Transition energy: 9670.51 eV
Orbital energy: -1.35 eV

HOMO

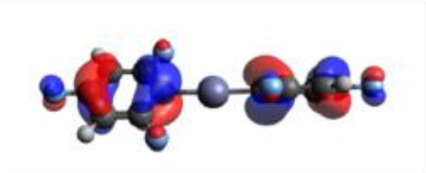

Zn 4.1% of which 0.4% is Zn4p
C: 62.6%
H: 0.8%
F: 10.3%
Transition energy: 9666.11 eV
Orbital energy: 9.60 eV

HOMO-1

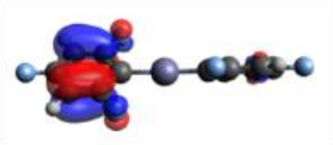

Zn 0.0% of which 0.0% is Zn4p
C: 63.1%
H: 3.0%
F: 10.4%
Transition energy: 9665.97 eV
Orbital energy: 9.74 eV

HOMO-2

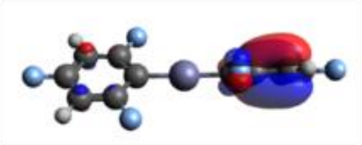

Zn 0.0% of which 0.0% is Zn4p
C: 63.2%
H: 3.0%
F: 10.4%
Transition energy: 9665.97 eV
Orbital energy: 9.75 eV

HOMO-3

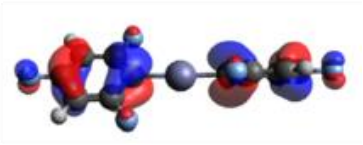

Zn 3.1% of which 2.4% is Zn4p
C: 62.4%
H: 0.9%
F: 10.5%
Transition energy: 9665.94 eV
Orbital energy: 9.77 eV

Supplementary Figure 23. Calculated molecular orbitals of eclipsed Zn(2,4,6-$C_6F_3H_2$)$_2$. The percentage of Zn 4p character has been listed for all key orbitals involved in both XAS and XES alongside the calculated transition energy (from KS-DFT XES (OMOs) and TDDFT (UMOs)) and orbital energy.

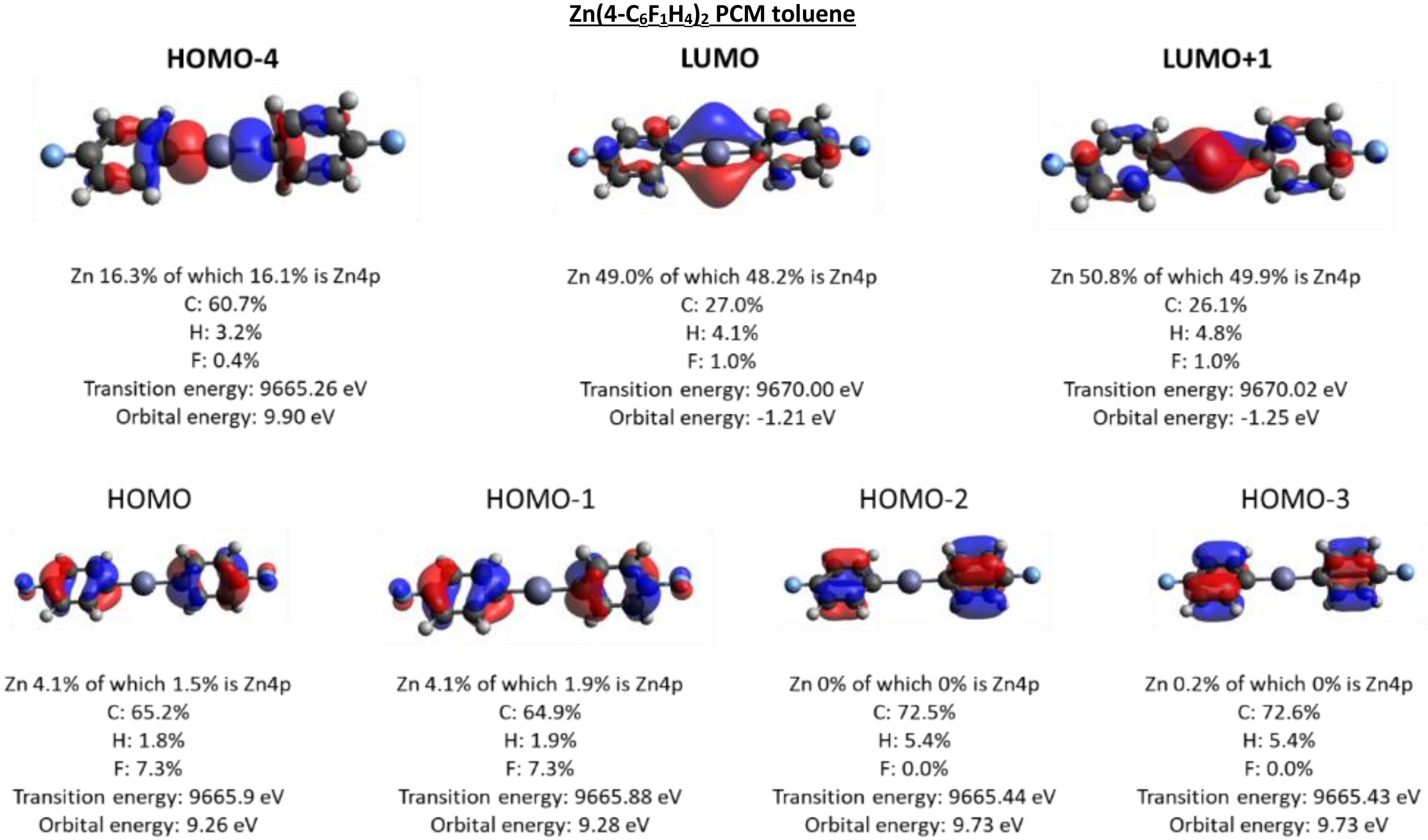

Supplementary Figure 24. Calculated molecular orbitals of eclipsed Zn(4-$C_6F_1H_4$)$_2$. The percentage of Zn 4p character has been listed for all key orbitals involved in both XAS and XES alongside the calculated transition energy (from KS-DFT XES (OMOs) and TDDFT (UMOs)) and orbital energy.

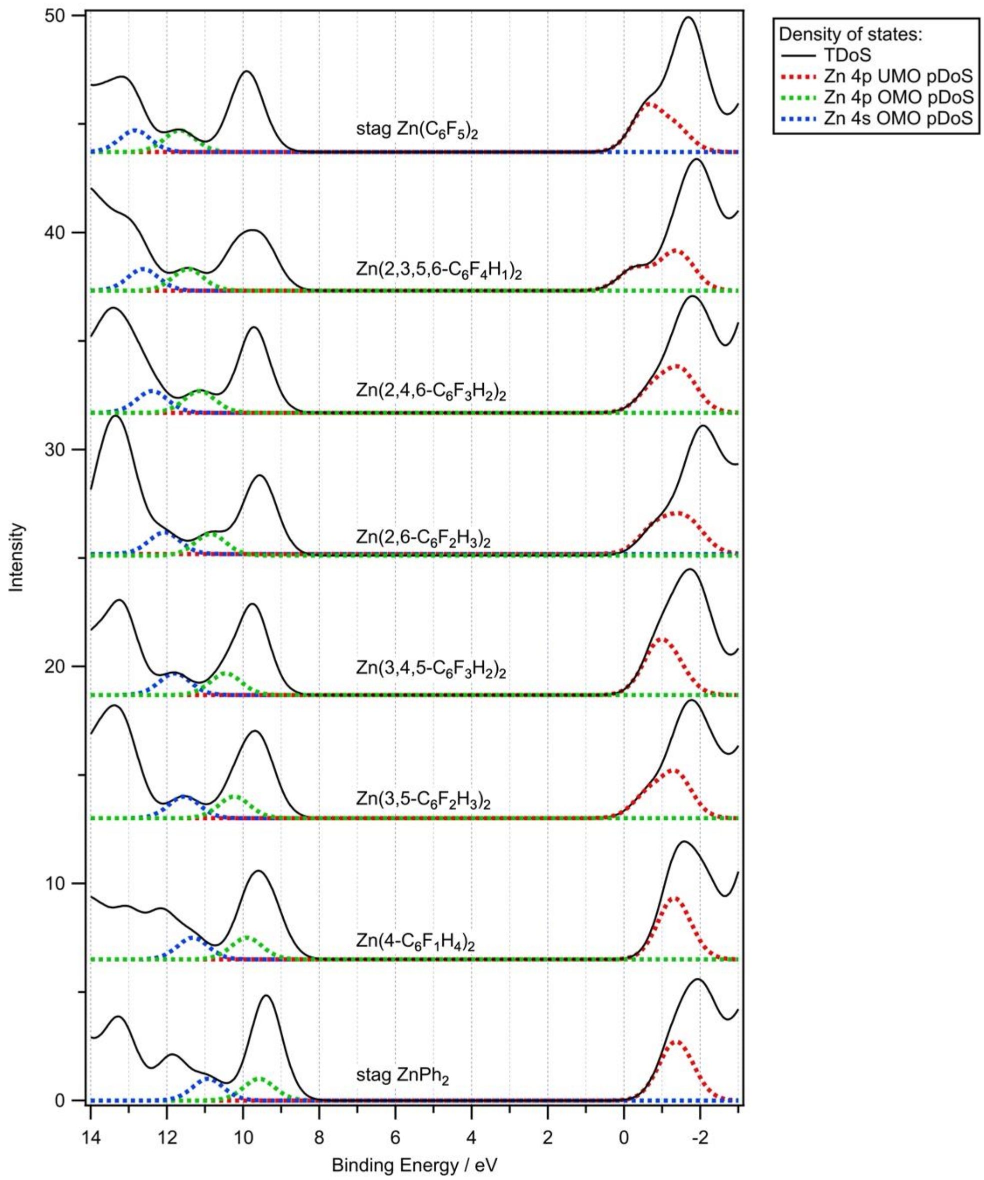

Supplementary Figure 25. Calculated total density-of-states (tDoS) and Zn 4p/Zn 4s (dotted line) partial density-of-states (pDoS) of ZnR_2 complexes (where R = aryl) showing the energy gap of the valence occupied and unoccupied molecular orbitals.

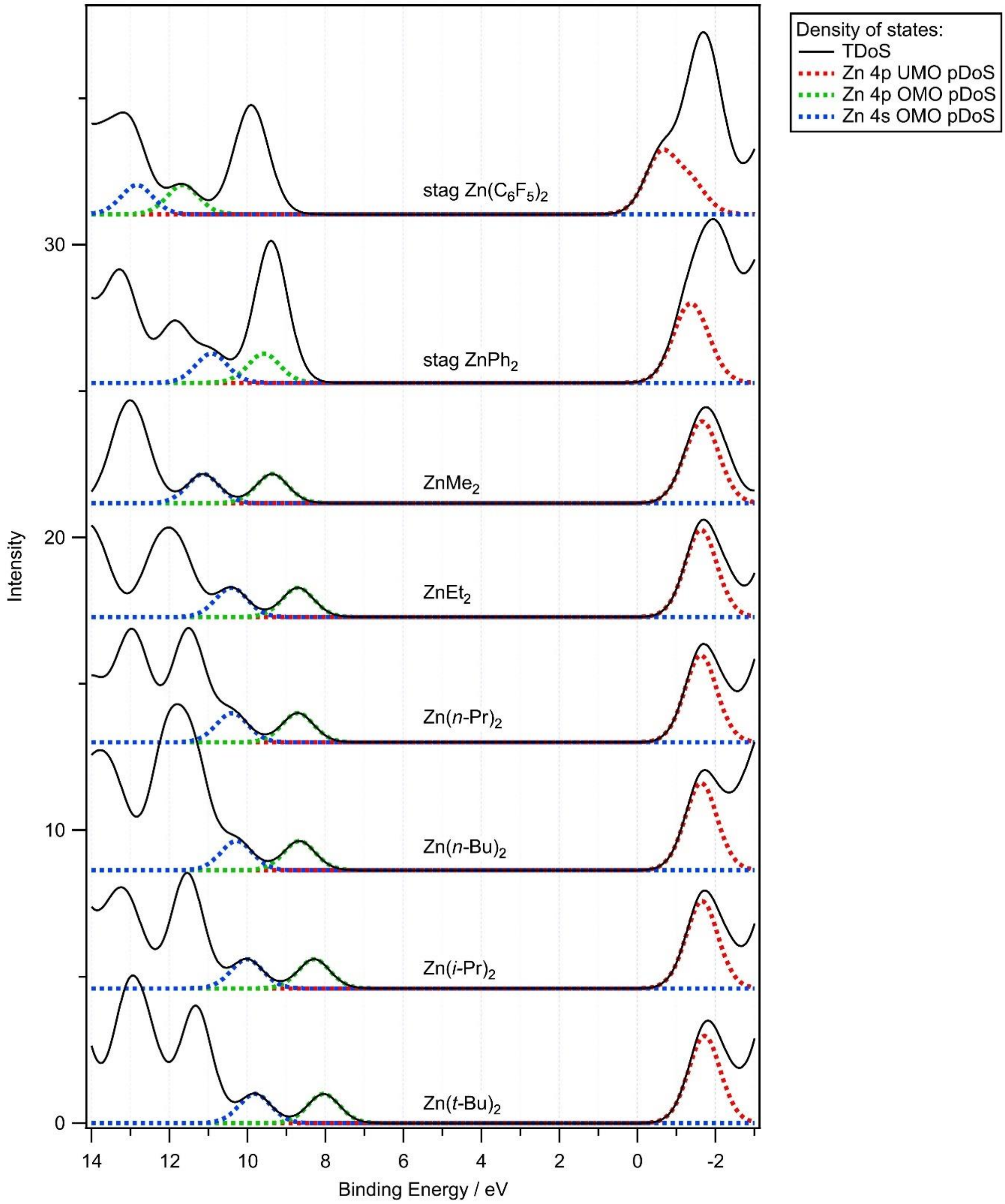

Supplementary Figure 26. Calculated total density-of-states (tDoS) and Zn 4p/Zn 4s (dotted line) partial density-of-states (pDoS) of ZnR_2 (including staggered $ZnPh_2$ and $Zn(C_6F_5)_2$) complexes showing the energy gap and identity of the valence occupied and unoccupied molecular orbitals.

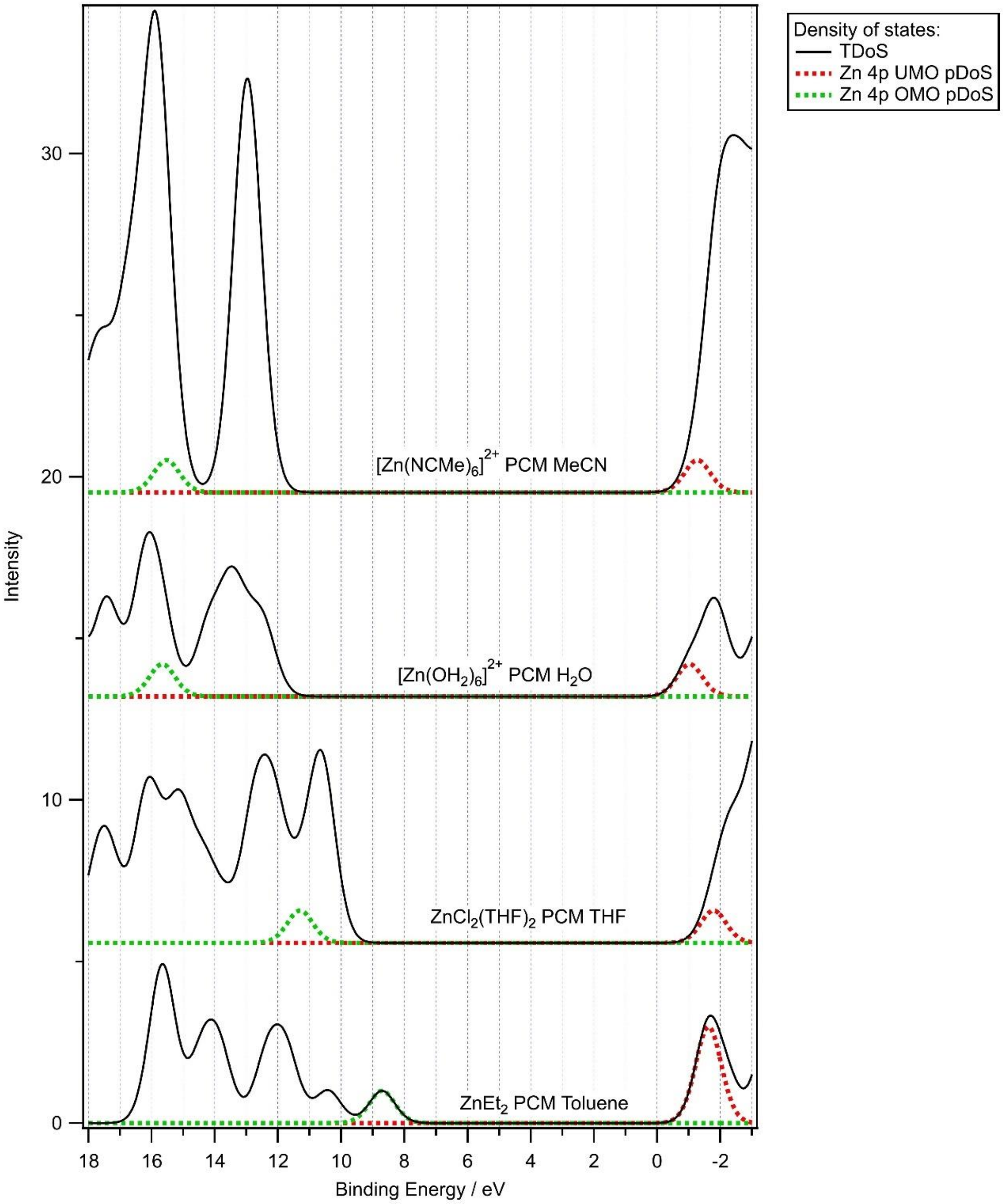

Supplementary Figure 27. Calculated total density-of-states (tDoS) and Zn 4p (dotted line) partial density-of-states (pDoS) of organozinc complexes showing the energy gap and identity of the valence occupied and unoccupied molecular orbitals.

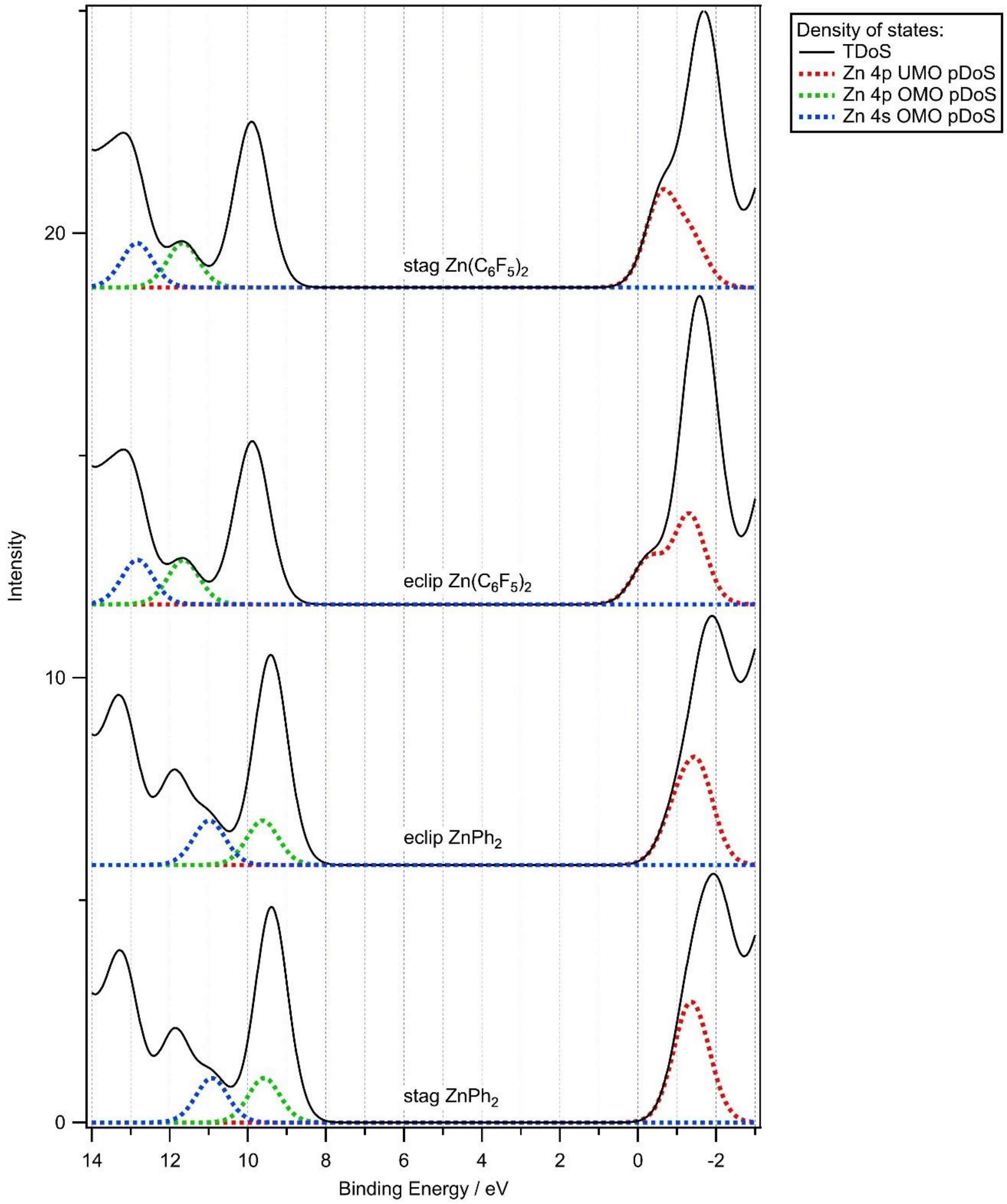

Supplementary Figure 28. Calculated total density-of-states (tDoS) and Zn 4p/Zn 4s (dotted line) partial density-of-states (pDoS) of the staggered and eclipsed $ZnPh_2$ and $Zn(C_6F_5)_2$ complexes showing the energy gap and identity of the valence occupied and unoccupied molecular orbitals.

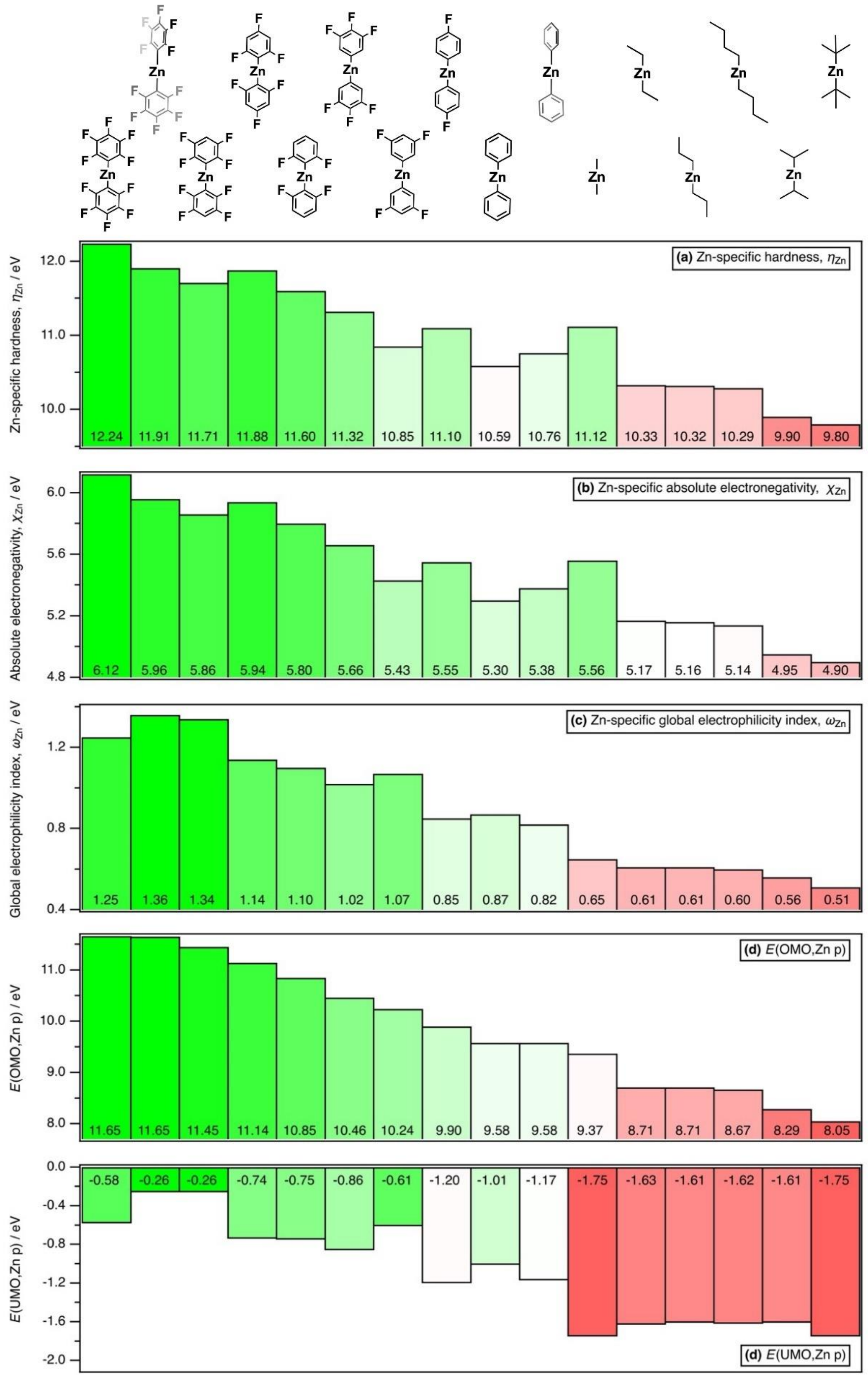

Supplementary Figure 29. Electronic structure properties for ZnR_2: (a) zinc-specific hardness (μ_{Zn}), (b) zinc-specific absolute electronegativity (χ_{Zn}), (c) global electrophilicity index (ω_{Zn}), (d) *E*(OMO,Zn p), (e) *E*(UMO,Zn p).

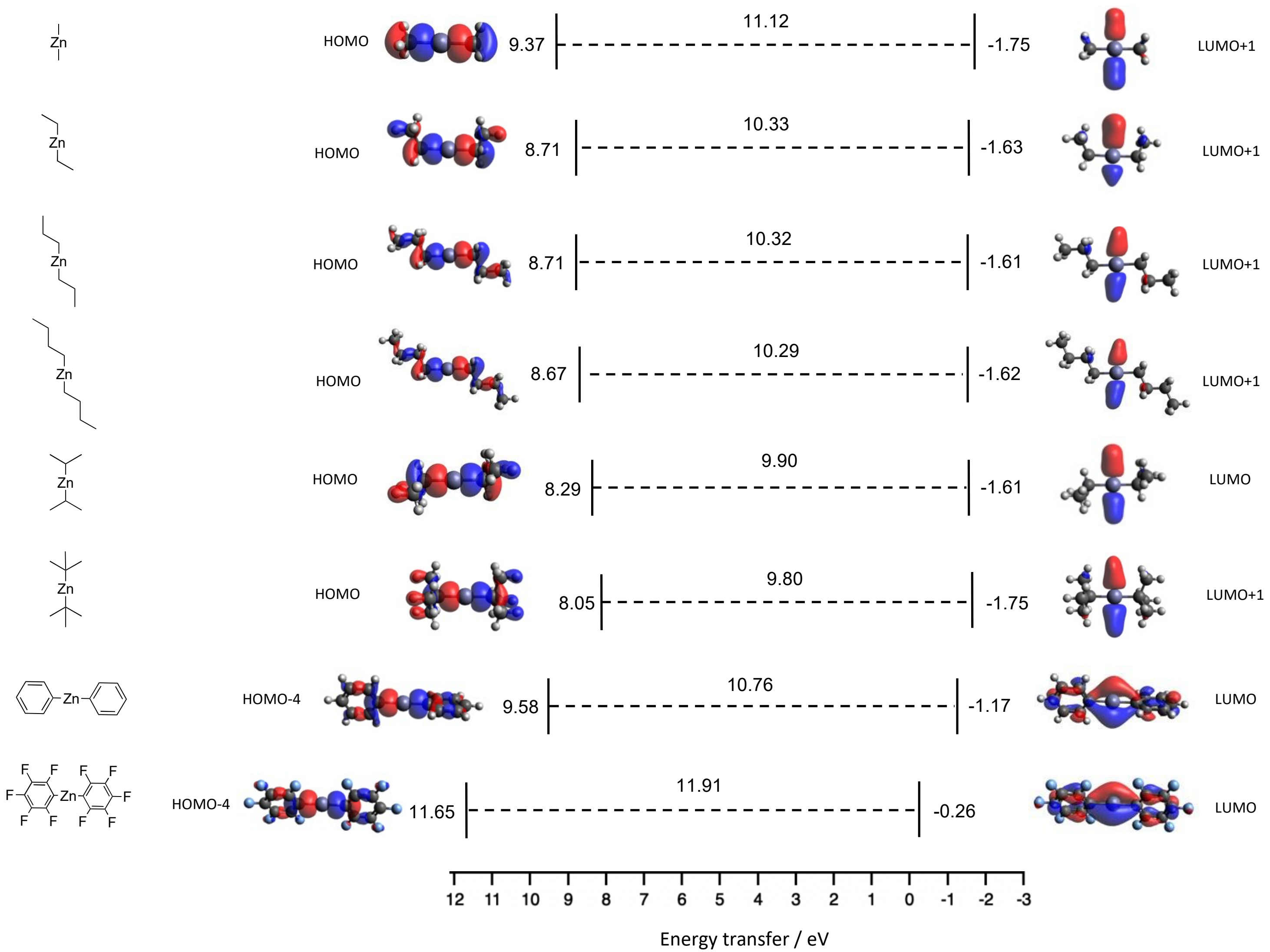

Supplementary Figure 30. Calculated Zn-specific occupied to unoccupied molecular orbital gap, *i.e.* Zn-specific hardness, η_{Zn}, for ZnR_2 compounds (R = Me, Et, *n*-Pr, *n*-Bu, *i*-Pr, *t*-Bu, Ph, C_6F_5).

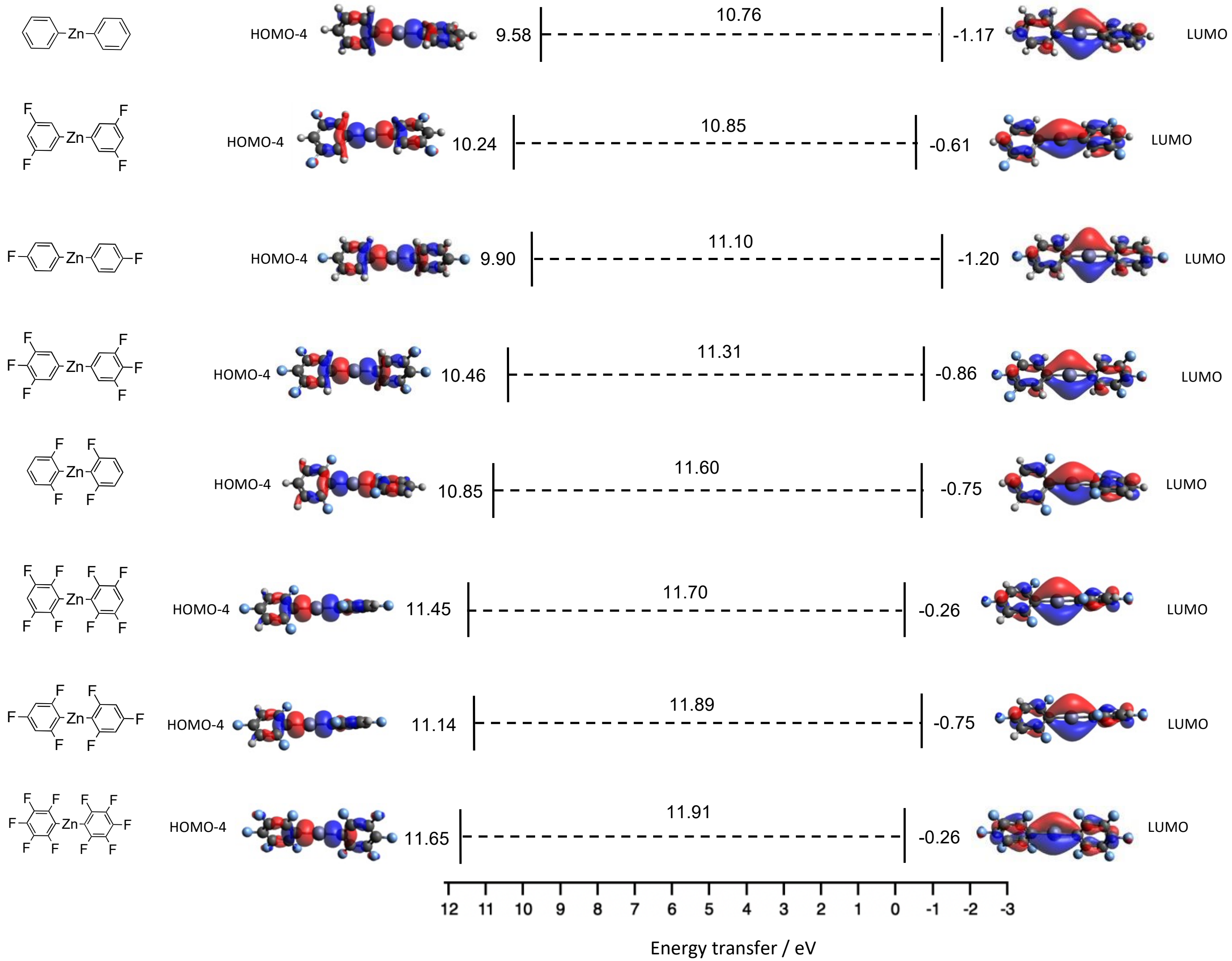

Supplementary Figure 31. Calculated Zn-specific occupied to unoccupied molecular orbital gap, *i.e.* Zn-specific hardness, η_{Zn}, for ZnR_2 compounds (R = Ph, 3,5-$C_6F_2H_3$, 4-$C_6F_1H_4$, 3,4,5-$C_6F_3H_2$, 2,6-$C_6F_2H_3$, 2,3,5,6-$C_6F_4H_1$, 2,4,6-$C_6F_3H_2$, C_6F_5).

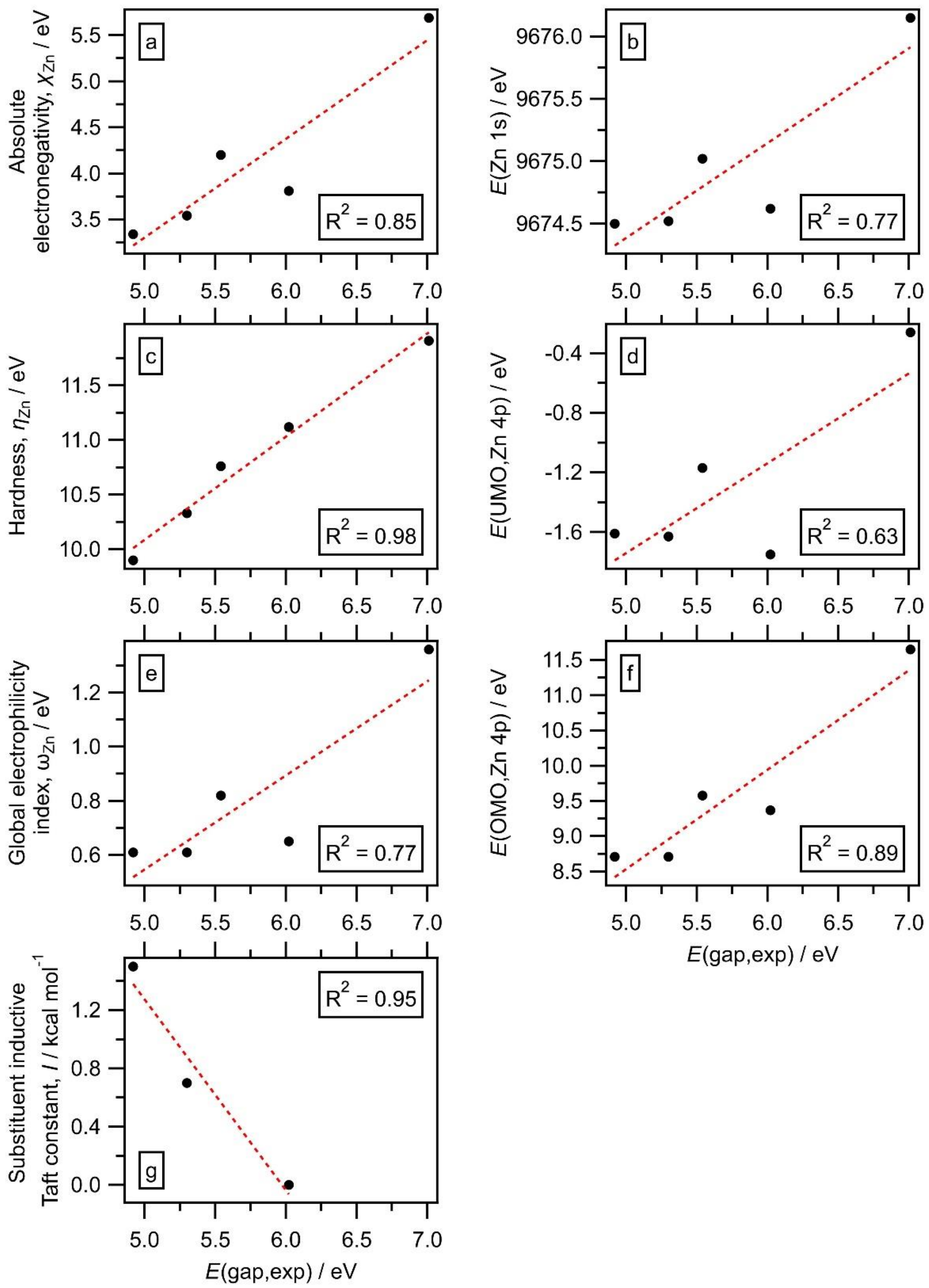

Supplementary Figure 32. Linear correlation fits for calculated descriptors against experimental R-VtC-XES gap size, *E*(gap,exp), for ZnR_2 complexes: (a) Zn-specific hardness (μ_{Zn}) versus *E*(gap,exp); (b) Zn-specific absolute electronegativity (χ_{Zn}) versus *E*(gap,exp), (c) zinc-specific global electrophilicity index (ω_{Zn}) versus *E*(gap,exp), (d) Taft inductive substituent constant (I_R) versus *E*(gap,exp), (e) *E*(Zn 1s) versus *E*(gap,exp), (f) *E*(UMO,Zn p) versus *E*(gap,exp), and (g) *E*(OMO,Zn p) versus *E*(gap,exp). Data for I_R taken from reference [1].

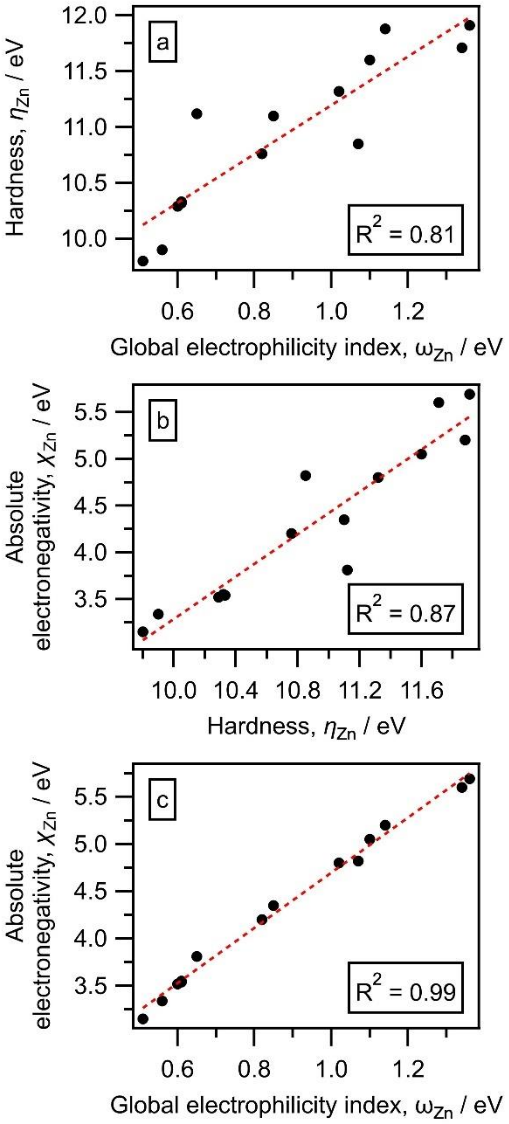

Supplementary Figure 33. Linear correlation fits for calculated descriptors for ZnR_2 complexes: (a) Zn-specific hardness (μ_{Zn}) versus zinc-specific global electrophilicity index (ω_{Zn}); (b) Zn-specific absolute electronegativity (χ_{Zn}) versus Zn-specific hardness (μ_{Zn}); (c) Zn-specific absolute electronegativity (χ_{Zn}) versus zinc-specific global electrophilicity index (ω_{Zn}).

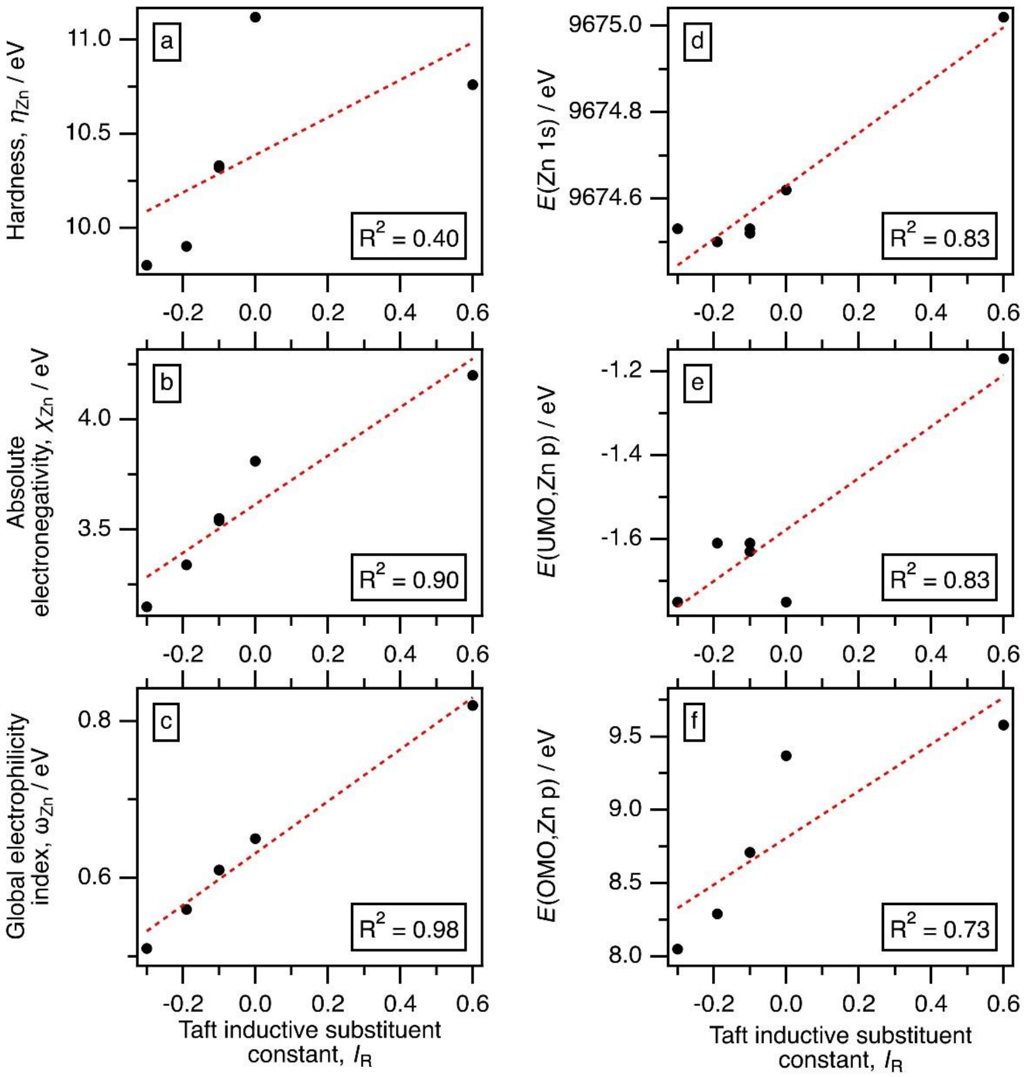

Supplementary Figure 34. Linear correlation fits for calculated descriptors against Taft inductive substituent constant (I_R) for ZnR_2 complexes: (a) Zn-specific hardness (μ_{Zn}) versus I_R; (b) Zn-specific absolute electronegativity (χ_{Zn}) versus I_R; (c) zinc-specific global electrophilicity index (ω_{Zn}) versus I_R; (d) E(Zn 1s) versus I_R; (e) E(UMO,Zn p) versus I_R; (f) E(OMO,Zn p) versus I_R. Data for I_R taken from reference[1].

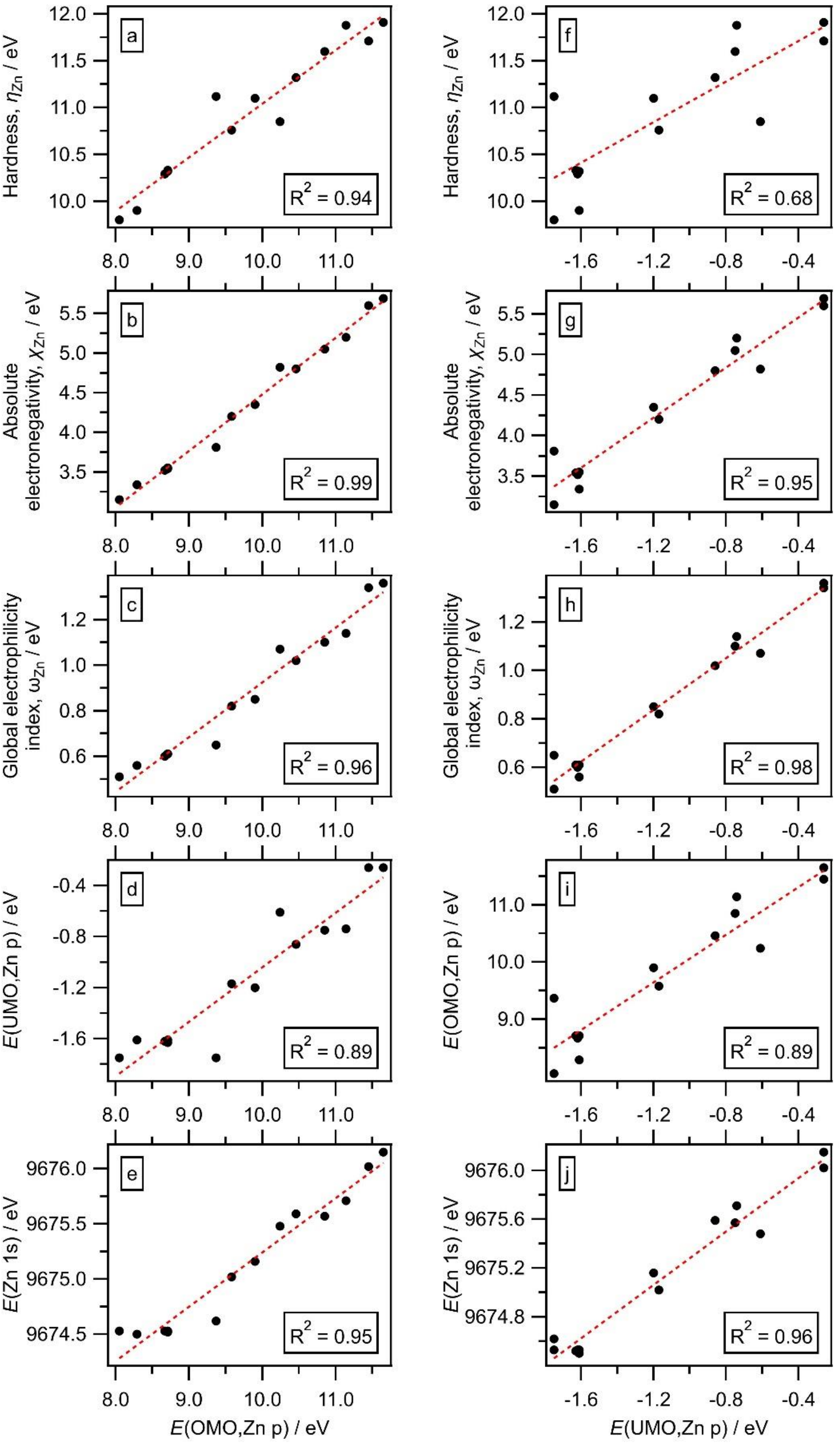

Supplementary Figure 35. Linear correlation fits for calculated descriptors against E(OMO,Zn p) (a-e) and E(UMO,Zn p) (f-j) for ZnR_2 complexes: (a) Zn-specific hardness (μ_{Zn}) versus E(OMO,Zn p) (b) Zn-specific absolute electronegativity (χ_{Zn}) versus E(OMO,Zn p); (c) zinc-specific global electrophilicity index (ω_{Zn}) versus E(OMO,Zn p); (f) E(UMO,Zn p) versus E(OMO,Zn p); (e) E(Zn 1s) versus E(OMO,Zn p); (f) Zn-specific hardness (μ_{Zn}) versus E(UMO,Zn p) (g) Zn-specific absolute electronegativity (χ_{Zn}) versus E(UMO,Zn p); (h) zinc-specific global electrophilicity index (ω_{Zn}) versus E(UMO,Zn p); (i) E(OMO,Zn p) versus E(UMO,Zn p); (j) E(Zn 1s) versus E(UMO,Zn p).

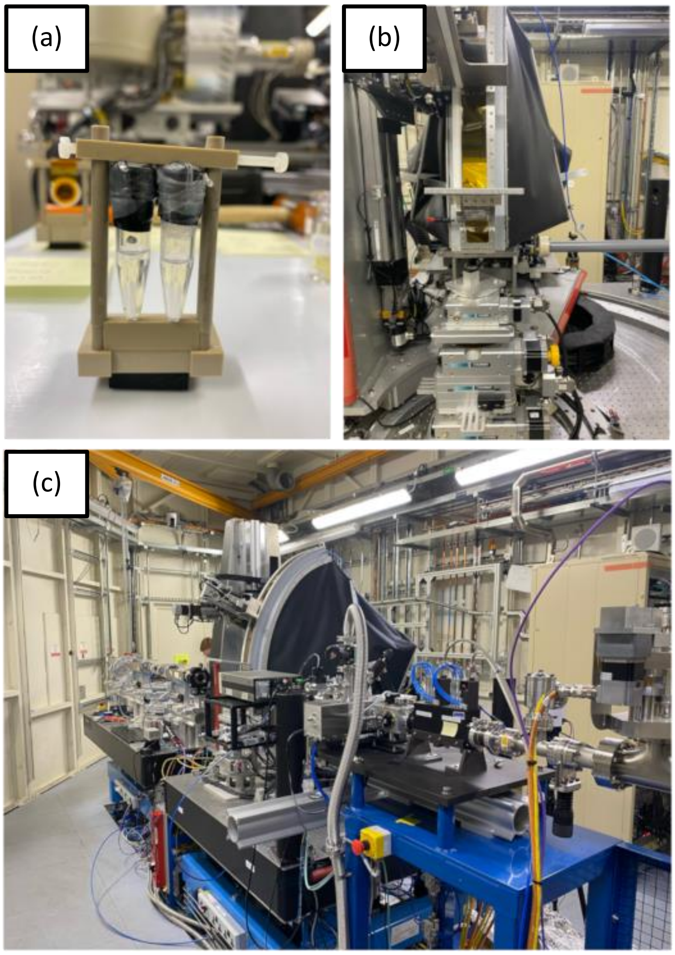

Supplementary Figure 36. Photographs of the experimental setup at I20-Scanning Diamond Lightsource.[3] (a) Small plastic vice containing two centrifuge vials used during the experiment, (b) Sample holder in line with the incident X-ray beam, samples are placed on the small rectangle platform and aligned with the incident X-rays, (c) full setup of the I20-Scanning spectrometer.[4]

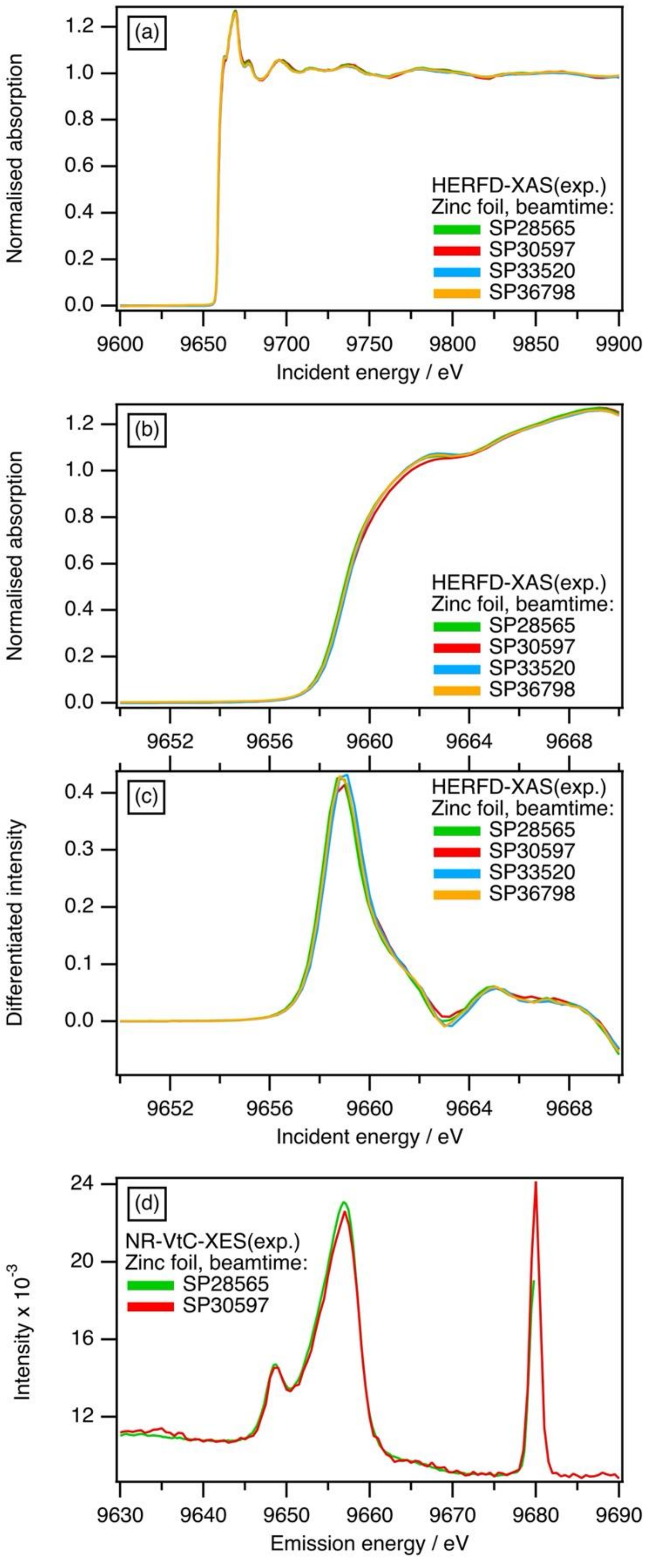

Supplementary Figure 37. Zinc foil reference data from each I20 beamtime (separate beamtimes denoted by naming scheme SPxxxxx): (a) normalised Zn 1s edge HERFD-XA spectra (b) normalised Zn 1s edge HERFD-XA spectra zoomed into the XANES region, (c) derivative plot of Zn 1s XANES region showing the first inflection and (d) NR-VtC-XE spectra of Zn foil (E_i = 9680.0 eV). The energy of the monochromator was calibrated to E_i = 9659.0 eV at the beginning of each beamtime to ensure cross-beamtime data comparability.

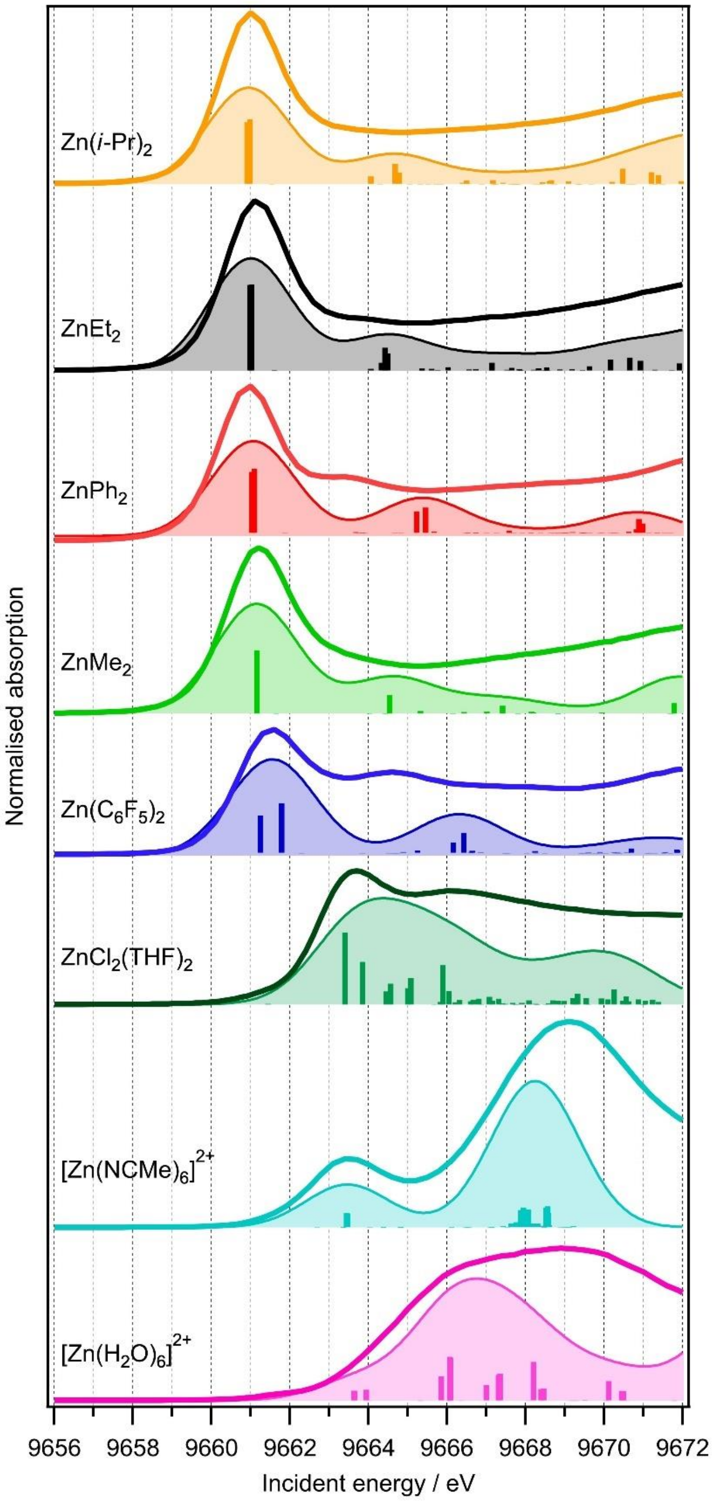

Supplementary Figure 38. Zn 1s HERFD-XA (y intensities normalised) and calculated TDDFT Zn 1s XA spectra of organozinc compounds ZnR_2 (R = Me, Et, *i*-Pr, Ph, C_6F_5, concentration 0.1 M for all apart from C_6F_5 which was 0.033 M), $ZnCl_2(THF)_2$ (0.1 M $ZnCl_2$ in THF), $[Zn(NCMe)_6]^{2+}$ (0.1 M $Zn(OTf)_2$ in MeCN) and $[Zn(OH_2)_6]^{2+}$ (0.1 M $ZnCl_2$ in H_2O). To achieve the best agreement with the experimental data, a Gaussian broadening with a full width at half maximum (FWHM) of 2.5 eV and an energy shift of -8.90 eV were applied.

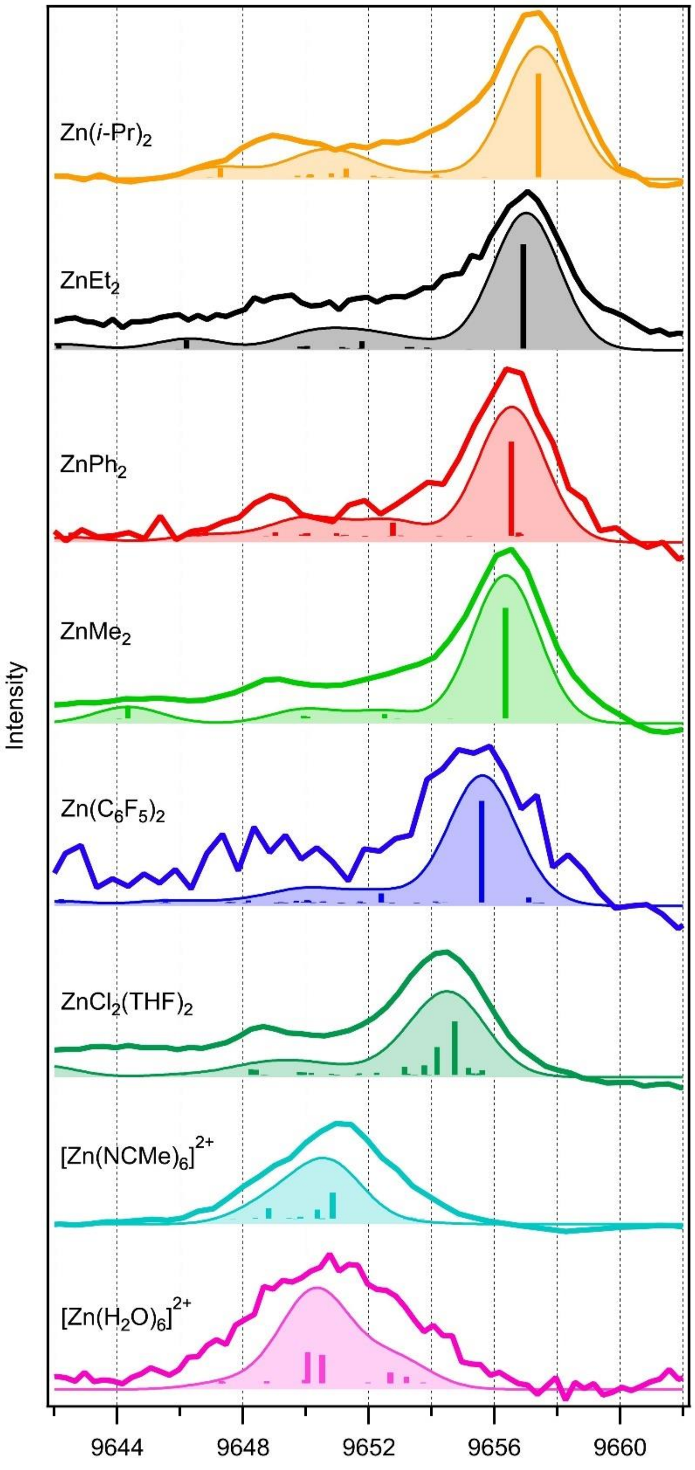

Supplementary Figure 39. Zn 1s NR-VtC-XE and calculated KS-DFT Zn 1s NR-VtC-XE spectra of organozinc compounds ZnR_2 (R = Me, Et, *i*-Pr, Ph, C_6F_5, concentration 0.1 M for all apart from C_6F_5 which was 0.033 M), $ZnCl_2(THF)_2$ (0.1 M $ZnCl_2$ in THF), $[Zn(NCMe)_6]^{2+}$ (0.1 M $Zn(OTf)_2$ in MeCN) and $[Zn(OH_2)_6]^{2+}$ (0.1 M $ZnCl_2$ in H_2O). To achieve the best agreement with the experimental data, a Gaussian broadening with a full width at half maximum (FWHM) of 2.5 eV and an energy shift of -8.90 eV were applied.

Supplementary Notes

Cartesian coordinates for all calculated structures (TDDFT, XES, RASPT2)

$[Zn(OH_2)_6]^{2+}$ CPCM water

Centre number	Atom type	Coordinates (Angstroms)		
		X	Y	Z
1	Zn	-1.84109	0.12925	0.49999
2	O	-0.74904	-1.41264	-0.43321
3	H	-0.92649	-2.31444	-0.14190
4	O	-1.16356	-0.41012	2.42331
5	H	-1.17409	0.30111	3.07425
6	O	-2.51861	0.66861	-1.42334
7	H	-2.50809	-0.04262	-2.07428
8	O	-2.93314	1.67115	1.43317
9	H	-3.89135	1.56545	1.41596
10	O	-3.50294	-1.12761	0.81875
11	H	-3.37618	-1.82654	1.47070
12	O	-0.17923	1.38611	0.18124
13	H	0.19437	1.80998	0.96249
14	H	0.20917	-1.30694	-0.41600
15	H	-0.29429	-0.82222	2.48811
16	H	-3.87655	-1.55146	0.03749
17	H	-2.75569	2.57295	1.14186
18	H	-3.38787	1.08072	-1.48815
19	H	-0.30598	2.08504	-0.47072

$[Zn(NCMe)_6]^{2+}$ CPCM MeCN

Centre number	Atom type	Coordinates (Angstroms)		
		X	Y	Z
1	Zn	-3.44958	0.03696	0.18915
2	N	-3.63638	1.80356	1.39077
3	N	-4.91154	0.82959	-1.16768
4	N	-1.89009	0.97978	-0.94175
5	N	-1.98801	-0.74976	1.54614
6	N	-3.27345	-1.72030	-1.02389
7	N	-5.00658	-0.90246	1.32444
8	C	-5.82903	-1.40026	1.94492
9	C	-5.70671	1.25854	-1.86979
10	C	-3.75854	2.76669	1.99631
11	C	-1.06227	1.48412	-1.54967
12	C	-1.19632	-1.16984	2.25746
13	C	-3.18222	-2.65745	-1.67395
14	C	-3.06678	-3.84803	-2.49951
15	H	-2.84360	-4.70417	-1.86266
16	H	-2.26273	-3.70513	-3.22164
17	H	-4.00856	-4.01260	-3.02311
18	C	-0.18984	-1.70241	3.16071
19	H	0.35743	-2.49822	2.65527
20	H	-0.68040	-2.09862	4.04980
21	H	0.49621	-0.90344	3.44203
22	C	-0.01020	2.12377	-2.32183
23	H	0.95882	1.79929	-1.94247
24	H	-0.10203	3.20548	-2.22378
25	H	-0.11160	1.83791	-3.36884
26	C	-3.91716	3.99200	2.76179
27	H	-3.10913	4.67864	2.50929
28	H	-3.88292	3.75682	3.82560
29	H	-4.87755	4.44451	2.51414
30	C	-6.71724	1.80259	-2.76161
31	H	-7.21852	2.63477	-2.26719
32	H	-7.44161	1.02329	-2.99859
33	H	-6.23731	2.15144	-3.67600
34	C	-6.87331	-2.03054	2.73519
35	H	-6.42846	-2.47316	3.62652
36	H	-7.35522	-2.80559	2.13923
37	H	-7.60661	-1.27727	3.02340

$ZnCl_2(THF)_2$ CPCM THF

Centre number	Atom type	Coordinates (Angstroms)		
		X	Y	Z
1	Zn	-1.12403	5.59037	0.46907
2	Cl	-0.74483	3.99365	-1.02534
3	Cl	-1.72512	5.46728	2.60734
4	O	-2.28059	7.05282	-0.36733
5	O	0.57913	6.76432	0.40646
6	C	-3.36467	7.70214	0.34548
7	C	-4.04477	8.56876	-0.70108
8	H	-2.92893	8.25736	1.17476
9	H	-4.02950	6.92908	0.73522
10	C	-3.85466	7.74403	-1.97596
11	H	-3.53367	9.52997	-0.78898
12	H	-5.09131	8.74950	-0.45801
13	C	0.57948	8.03760	1.10630
14	C	1.87591	6.12123	0.52917
15	C	2.80420	7.21229	1.03439
16	H	2.13809	5.72183	-0.44898
17	H	1.78466	5.30196	1.24647
18	C	1.86891	8.04698	1.91157
19	H	-0.32163	8.08762	1.71664
20	H	0.55836	8.82788	0.35326
21	H	1.71505	7.55712	2.87553
22	H	2.23126	9.05953	2.08629
23	H	3.18031	7.80859	0.20025
24	H	3.65201	6.80024	1.58071
25	C	-2.44479	7.20294	-1.80176
26	H	-2.27753	6.23033	-2.26128
27	H	-1.68767	7.90870	-2.15022
28	H	-3.95332	8.33220	-2.88767
29	H	-4.57387	6.92283	-2.00852

$[ZnCl_4]^{2-}$ CPCM Ionic liquid (dielectric constant = 11.40)

Centre number	Atom type	Coordinates (Angstroms)		
		X	Y	Z
1	Zn	0.02652	1.69032	0.1098
2	Cl	-0.84475	3.75417	-0.38794
3	Cl	-1.03949	0.10766	-1.17472
4	Cl	-0.31217	1.24883	2.33679
5	Cl	2.2746	1.65581	-0.35624

$ZnMe_2$ CPCM toluene

Centre number	Atom type	Coordinates (Angstroms)		
		X	Y	Z
1	Zn	-0.16678	1.33925	0.00119
2	C	-2.10159	1.37855	0.00023
3	H	-2.49282	1.54702	-1.00721
4	H	-2.48203	2.17816	0.64224
5	H	-2.51732	0.43456	0.36400
6	C	1.76798	1.29956	0.00002
7	H	2.14240	0.28554	-0.16713
8	H	2.17188	1.64925	0.95442
9	H	2.17675	1.93875	-0.78776

$ZnEt_2$ CPCM toluene

Centre number	Atom type	Coordinates (Angstroms)		
		X	Y	Z
1	Zn	-0.18088	1.89935	-0.29275
2	C	-2.11277	1.71664	-0.17847
3	C	1.75056	2.10353	-0.37415
4	C	2.27925	3.35762	0.34022
5	H	2.06084	2.12200	-1.42532
6	H	2.22118	1.21111	0.05415
7	H	1.85828	4.27220	-0.08721
8	H	3.36957	3.44420	0.27544
9	H	2.01953	3.35545	1.40274
10	C	-2.63972	1.52258	1.25249
11	H	-2.42844	0.87334	-0.80340
12	H	-2.57994	2.60234	-0.62422
13	H	-2.22380	0.62358	1.71643
14	H	-3.73064	1.42603	1.28438
15	H	-2.37271	2.36449	1.89771

$Zn(\textit{i}\text{-}Pr)_2$ CPCM toluene

Centre number	Atom type	Coordinates (Angstroms)		
		X	Y	Z
1	Zn	0.28391	0.81220	-1.31546
2	C	-1.09184	0.98784	0.05924
3	C	-1.94389	2.24741	-0.14187
4	H	-2.70479	2.34655	0.64402
5	H	-2.47674	2.22077	-1.09818
6	H	-1.34083	3.15986	-0.13165
7	C	-1.98824	-0.25464	0.13210
8	H	-0.57559	1.08435	1.02290
9	H	-2.52170	-0.41813	-0.81020
10	H	-2.75050	-0.15410	0.91650
11	H	-1.41771	-1.16359	0.34367
12	C	1.65837	0.63769	-2.69162
13	C	2.54367	1.88738	-2.77651
14	H	3.30369	1.78796	-3.56323
15	H	3.07943	2.06175	-1.83747
16	H	1.96461	2.79000	-2.99191
17	C	2.52194	-0.61279	-2.48312
18	H	1.14073	0.52931	-3.65329
19	H	3.05631	-0.57456	-1.52803
20	H	3.28226	-0.71081	-3.26973
21	H	1.92700	-1.53059	-2.48563

Zn(*n*-Pr)$_2$ CPCM toluene

Centre number	Atom type	Coordinates (Angstroms)		
		X	Y	Z
1	Zn	-0.51314	1.03177	0.02773
2	C	-2.45628	1.00723	0.04511
3	C	1.43001	1.05589	0.02582
4	C	2.04652	2.46161	-0.01733
5	H	1.80011	0.47454	-0.82813
6	H	1.79535	0.53039	0.91737
7	C	3.57550	2.44691	-0.01188
8	H	1.69368	3.05086	0.83648
9	H	1.69920	2.99484	-0.90936
10	H	3.95280	1.95367	0.88871
11	H	3.99398	3.45713	-0.04412
12	H	3.95852	1.89498	-0.87523
13	C	-3.07277	-0.39568	-0.04075
14	H	-2.83697	1.61860	-0.78278
15	H	-2.81072	1.50004	0.95952
16	C	-4.60130	-0.38192	-0.01385
17	H	-2.70834	-1.01537	0.78629
18	H	-2.73749	-0.89581	-0.95629
19	H	-4.96616	0.07679	0.90983
20	H	-5.02030	-1.39040	-0.07850
21	H	-4.99599	0.20209	-0.85044

Zn(*n*-Bu)$_2$ CPCM toluene

Centre number	Atom type	Coordinates (Angstroms)		
		X	Y	Z
1	Zn	-0.50346	1.05215	0.02787
2	C	-2.44534	1.09010	0.04075
3	C	1.43847	1.01042	0.02920
4	C	2.10415	2.39288	0.00400
5	H	1.78750	0.42672	-0.83192
6	H	1.78330	0.46105	0.91439
7	C	3.63202	2.33653	0.01171
8	H	1.77336	2.98713	0.86462
9	H	1.78082	2.95138	-0.88301
10	C	-3.10617	-0.29442	0.00024
11	H	-2.80251	1.68479	-0.80938
12	H	-2.78626	1.62515	0.93619
13	C	-4.63408	-0.24417	0.02034
14	H	-2.76676	-0.90005	0.84950
15	H	-2.78760	-0.83855	-0.89737
16	C	-5.27730	-1.62804	-0.01959
17	H	-4.98067	0.35066	-0.83220
18	H	-4.95934	0.29010	0.92012
19	H	-4.98494	-2.16773	-0.92506
20	H	-6.36824	-1.56722	-0.00427
21	H	-4.96359	-2.22884	0.83902
22	C	4.28040	3.71836	-0.01237
23	H	3.96984	1.75328	-0.85229
24	H	3.96208	1.78751	0.90079
25	H	3.98309	4.27292	-0.90719
26	H	5.37118	3.65303	-0.00666
27	H	3.97590	4.30726	0.85772

Zn(*t*-Bu)$_2$ CPCM toluene

Centre number	Atom type	Coordinates (Angstroms)		
		X	Y	Z
1	C	-5.28643	1.91540	0.08240
2	C	-6.00733	3.03870	0.83459
3	H	-5.88146	4.00712	0.34046
4	H	-5.64497	3.14102	1.86232
5	H	-7.08813	2.83645	0.88783
6	C	-5.85998	1.82167	-1.33520
7	H	-6.94133	1.61892	-1.30124
8	H	-5.39642	1.01509	-1.91162
9	H	-5.72082	2.75164	-1.89495
10	C	-5.54332	0.59058	0.80826
11	H	-6.62195	0.37525	0.84786
12	H	-5.17836	0.61204	1.83969
13	H	-5.06203	-0.25301	0.30390
14	C	-1.43308	2.62098	-0.06589
15	C	-0.94175	3.16398	1.27981
16	H	-1.43663	4.10257	1.54761
17	H	0.13998	3.36387	1.24312
18	H	-1.11349	2.45381	2.09454
19	C	-1.12932	3.65366	-1.15633
20	H	-1.63847	4.60471	-0.97177
21	H	-1.43111	3.30266	-2.14815
22	H	-0.04945	3.86325	-1.19916
23	C	-0.67556	1.32777	-0.38448
24	H	0.40709	1.51759	-0.43925
25	H	-0.97937	0.90192	-1.34560
26	H	-0.83291	0.56260	0.38178
27	Zn	-3.36132	2.27345	0.00801

$[ZnPh_2]_2$ CPCM Toluene. XYZ coordinates taken from crystal structure.[2]

Centre number	Atom type	Coordinates (Angstroms)		
		X	Y	Z
1	Zn	0.47169	2.27883	2.75604
2	Zn	0.72579	4.81753	1.91871
3	C	0.87891	6.27955	0.63640
4	C	1.05016	7.56512	1.14973
5	C	1.11659	8.68036	0.33321
6	C	1.03511	8.53525	-1.03966
7	C	0.91900	7.28098	-1.57600
8	C	0.84008	6.17144	-0.75348
9	C	0.27435	4.45292	3.84944
10	C	1.30724	4.33517	4.77803
11	C	1.06055	4.38896	6.13789
12	C	-0.20848	4.56500	6.61419
13	C	-1.25027	4.68337	5.72863
14	C	-1.01446	4.63481	4.36276
15	C	-0.04035	0.78759	3.88746
16	C	0.06928	-0.48407	3.37414
17	C	-0.11450	-1.61328	4.15763
18	C	-0.42791	-1.47708	5.49548
19	C	-0.56318	-0.22940	6.03482
20	C	-0.37047	0.89720	5.25433
21	C	1.65430	2.76236	1.20977
22	C	1.34663	2.47888	-0.12208
23	C	2.30758	2.49475	-1.10670
24	C	3.59193	2.79628	-0.80951
25	C	3.95011	3.07837	0.49732
26	C	2.98339	3.06148	1.47093
27	H	1.12635	7.68703	2.11834
28	H	1.21858	9.57385	0.72446
29	H	1.06162	9.32199	-1.62303
30	H	0.88888	7.16674	-2.54961
31	H	0.75533	5.28298	-1.15773
32	H	2.22864	4.22769	4.46183
33	H	1.80429	4.28613	6.76829
34	H	-0.37130	4.60906	7.57980
35	H	-2.16408	4.80163	6.05984
36	H	-1.76560	4.73622	3.74237
37	H	0.28574	-0.59989	2.42554
38	H	-0.02642	-2.50668	3.76438
39	H	-0.55730	-2.27217	6.05383
40	H	-0.78992	-0.12992	6.98242
41	H	-0.46840	1.78520	5.65758
42	H	0.42193	2.26180	-0.36223
43	H	2.06053	2.28649	-2.03129

44	H	4.26922	2.81165	-1.51896
45	H	4.88087	3.29018	0.72146
46	H	3.24707	3.27091	2.39151

$ZnPh_2$ CPCM toluene (eclipsed)

Centre number	Atom type	Coordinates (Angstroms)		
		X	Y	Z
1	Zn	1.57090	4.00064	0.00134
2	C	-2.36654	3.10431	-1.22375
3	C	-3.02311	2.80367	-0.03633
4	C	-2.32576	2.85940	1.16463
5	C	-0.98062	3.21483	1.17221
6	C	-1.02151	3.45948	-1.20415
7	C	-0.29204	3.52276	-0.00906
8	H	-0.53655	3.69037	-2.14828
9	H	-2.90328	3.06218	-2.16577
10	H	-4.07206	2.52827	-0.04670
11	H	-2.83042	2.62516	2.09623
12	H	-0.46270	3.25015	2.12661
13	C	6.16457	5.20070	0.03481
14	C	5.46621	5.14317	-1.16536
15	C	5.50938	4.90073	1.22313
16	C	4.16450	4.54419	1.20522
17	C	4.12128	4.78637	-1.17134
18	C	3.43412	4.47872	0.01085
19	H	5.97004	5.37665	-2.09790
20	H	3.60247	4.74972	-2.12525
21	H	7.21320	5.47724	0.04394
22	H	3.68037	4.31385	2.15035
23	H	6.04692	4.94465	2.16458

$ZnPh_2$ CPCM toluene (staggered)

Centre number	Atom type	Coordinates (Angstroms)		
		X	Y	Z
1	Zn	1.57176	3.99869	-0.00855
2	C	-2.58027	3.69713	-0.71669
3	C	-2.95498	2.56965	0.00447
4	C	-1.99516	1.86465	0.72094
5	C	-0.67017	2.28953	0.71315
6	C	-1.25227	4.11257	-0.71785
7	C	-0.26282	3.42232	-0.00464
8	H	-0.98955	4.99706	-1.29141
9	H	-3.32339	4.25298	-1.27887
10	H	-3.98892	2.24215	0.00790
11	H	-2.27925	0.98323	1.28632
12	H	0.05743	1.71845	1.28306
13	C	6.09585	5.43615	0.00386
14	C	5.72329	4.30800	-0.71737
15	C	5.13476	6.13905	0.72067
16	C	3.81064	5.71144	0.71316
17	C	4.39615	3.88983	-0.71826
18	C	3.40545	4.57793	-0.00471
19	H	6.46742	3.75378	-1.27982
20	H	4.13513	3.00488	-1.29190
21	H	7.12911	5.76580	0.00708
22	H	3.08200	6.28094	1.28333
23	H	5.41716	7.02098	1.28611

$Zn(C_6F_5)_2$ CPCM toluene (eclipsed)

Centre number	Atom type	Coordinates (Angstroms)		
		X	Y	Z
1	Zn	1.58682	3.99292	-0.11693
2	C	-2.63162	3.92286	-0.04373
3	C	-2.92925	2.56899	-0.00597
4	C	-1.90494	1.63430	-0.00893
5	C	-0.59365	2.07923	-0.04964
6	C	-1.30341	4.31569	-0.08307
7	C	-0.25199	3.41801	-0.08640
8	C	6.10111	5.41751	0.06077
9	C	5.78571	4.08101	0.25344
10	C	5.09405	6.33762	-0.18943
11	C	3.78170	5.89633	-0.24230
12	C	4.45769	3.69145	0.19110
13	C	3.42303	4.57435	-0.05498
14	F	-1.04984	5.63961	-0.11828
15	F	-3.62240	4.81924	-0.04122
16	F	-4.19827	2.16681	0.03310
17	F	-2.19588	0.33093	0.02778
18	F	0.37898	1.14498	-0.05113
19	F	4.18565	2.38469	0.38332
20	F	6.75810	3.19722	0.49507
21	F	2.82795	6.81742	-0.48855
22	F	5.40226	7.62462	-0.37324
23	F	7.37028	5.81727	0.11656

$Zn(C_6F_5)_2$ CPCM toluene (staggered)

Centre number	Atom type	Coordinates (Angstroms)		
		X	Y	Z
1	Zn	1.61668	3.89509	0.13359
2	C	-2.41234	3.55699	-0.96850
3	C	-2.93020	2.60897	-0.09872
4	C	-2.11449	2.03118	0.86273
5	C	-0.78571	2.41798	0.93542
6	C	-1.07863	3.91061	-0.85125
7	C	-0.23104	3.36314	0.09347
8	C	6.06915	5.48100	-0.09744
9	C	5.69960	4.34865	-0.80798
10	C	5.14277	6.12834	0.70557
11	C	3.85279	5.62777	0.77326
12	C	4.39750	3.88747	-0.70351
13	C	3.43901	4.50979	0.07390
14	F	-0.59980	4.83234	-1.71368
15	F	-3.19822	4.10675	-1.89815
16	F	-4.21067	2.25403	-0.18722
17	F	-2.61815	1.11750	1.69756
18	F	-0.02328	1.83528	1.88233
19	F	4.06819	2.78709	-1.41132
20	F	6.59707	3.72913	-1.57922
21	F	2.97565	6.28199	1.56140
22	F	5.50243	7.21590	1.39336
23	F	7.31376	5.94606	-0.18822

$Zn(2,6\text{-}C_6F_2H_3)$ CPCM toluene

Centre number	Atom type	Coordinates (Angstroms)		
		X	Y	Z
1	Zn	1.61947	3.94706	-0.05298
2	C	-2.59380	3.92984	0.32837
3	C	-2.94377	2.64367	-0.06449
4	C	-1.96967	1.73151	-0.45203
5	C	-0.65048	2.14865	-0.43278
6	C	-1.25128	4.26461	0.31817
7	C	-0.22621	3.41160	-0.05530
8	C	6.13291	5.40779	0.03212
9	C	5.79396	4.19746	0.62508
10	C	5.16486	6.18848	-0.58834
11	C	3.86353	5.71879	-0.59306
12	C	4.46853	3.80283	0.57596
13	C	3.45115	4.52599	-0.02307
14	F	-0.91473	5.52517	0.70347
15	F	0.29801	1.25068	-0.81294
16	F	4.14263	2.61802	1.15945
17	F	2.91943	6.48608	-1.20207
18	H	-2.21987	0.72497	-0.76177
19	H	-3.98642	2.34881	-0.06838
20	H	-3.33573	4.65611	0.63443
21	H	5.40609	7.13466	-1.05560
22	H	7.16188	5.74615	0.05370
23	H	6.53178	3.57345	1.11298

$Zn(3,5\text{-}C_6F_2H_4)_2$ CPCM toluene

Centre number	Atom type	Coordinates (Angstroms)		
		X	Y	Z
1	Zn	1.58228	3.97315	-0.01573
2	C	-2.61019	3.91296	0.06110
3	C	-2.96940	2.58372	-0.06387
4	C	-1.92983	1.67872	-0.17437
5	C	-0.59824	2.05399	-0.16357
6	C	-1.29456	4.34026	0.07891
7	C	-0.25814	3.40601	-0.03497
8	C	6.11616	5.41932	0.01478
9	C	5.76123	4.12872	0.36104
10	C	5.07980	6.26298	-0.33927
11	C	3.75504	5.86466	-0.35496
12	C	4.45237	3.68040	0.36226
13	C	3.41891	4.55258	-0.00025
14	H	-1.10242	5.40225	0.18179
15	F	-3.59651	4.83059	0.17003
16	F	-2.23910	0.36867	-0.29857
17	H	0.15347	1.27842	-0.25659
18	H	4.26275	2.65209	0.64844
19	F	6.74669	3.27333	0.71315
20	H	3.00490	6.59105	-0.64647
21	F	5.38527	7.53339	-0.68492
22	H	-4.00453	2.26963	-0.07490
23	H	7.14622	5.74969	0.02058

$Zn(4\text{-}C_6F_3H_2)_2$ CPCM toluene

Centre number	Atom type	Coordinates (Angstroms)		
		X	Y	Z
1	Zn	1.60840	3.98171	-0.06183
2	C	-2.54269	3.75025	0.67242
3	C	-2.90618	2.63147	-0.05193
4	C	-1.99326	1.89467	-0.78147
5	C	-0.66403	2.30581	-0.77710
6	C	-1.20611	4.13670	0.65746
7	C	-0.23255	3.43136	-0.06250
8	C	6.10703	5.38105	0.02881
9	C	5.69318	4.40789	0.91775
10	C	5.24930	5.95742	-0.88808
11	C	3.92445	5.53256	-0.90551
12	C	4.36238	4.00393	0.87658
13	C	3.44368	4.54988	-0.02989
14	H	-0.92853	5.01675	1.22961
15	H	0.04728	1.72136	-1.35280
16	H	4.04479	3.23893	1.57870
17	H	3.25665	5.99036	-1.62906
18	H	-2.32138	1.02340	-1.33569
19	F	-4.20342	2.24298	-0.04647
20	H	-3.29123	4.29925	1.23116
21	H	5.61566	6.71801	-1.56722
22	F	7.39949	5.78438	0.05735
23	H	6.39935	3.98225	1.62065

$Zn(2,3,5,6\text{-}C_6F_4H_1)_2$ CPCM toluene

Centre number	Atom type	Coordinates (Angstroms)		
		X	Y	Z
1	Zn	1.56168	4.03712	-0.00304
2	C	-2.65526	3.90037	0.00215
3	C	-2.95346	2.55250	-0.06840
4	C	-1.90351	1.65462	-0.11657
5	C	-0.59335	2.10523	-0.09352
6	C	-1.33716	4.32899	0.02332
7	C	-0.27253	3.44840	-0.02398
8	C	6.12207	5.38175	0.00073
9	C	5.76156	4.08137	0.29949
10	C	5.11761	6.28521	-0.29197
11	C	3.78988	5.88796	-0.28397
12	C	4.42741	3.70595	0.30242
13	C	3.40713	4.59237	0.01109
14	F	-1.10087	5.65769	0.09198
15	F	-3.64938	4.80180	0.04988
16	F	-2.15380	0.33721	-0.18675
17	F	0.39767	1.18759	-0.14142
18	F	4.12775	2.42270	0.60148
19	F	6.70944	3.17505	0.58792
20	F	2.84761	6.81091	-0.57821
21	F	5.43055	7.55704	-0.58771
22	H	-3.97974	2.20991	-0.08648
23	H	7.16086	5.68482	-0.00479

Zn(3,4,5-$C_6F_3H_2$)$_2$ CPCM toluene

Centre number	Atom type	Coordinates (Angstroms)		
		X	Y	Z
1	Zn	1.60569	3.99010	-0.04415
2	C	-2.51958	3.71975	0.68205
3	C	-2.92847	2.62329	-0.05949
4	C	-1.97812	1.93608	-0.79708
5	C	-0.65210	2.32690	-0.79934
6	C	-1.19809	4.12599	0.69246
7	C	-0.23511	3.43413	-0.05133
8	C	6.13173	5.38183	0.01967
9	C	5.66697	4.43532	0.91825
10	C	5.23971	5.91165	-0.89855
11	C	3.91619	5.51330	-0.92475
12	C	4.34717	4.02360	0.90722
13	C	3.44299	4.55723	-0.01859
14	H	-0.94665	4.99202	1.29473
15	H	0.03917	1.74508	-1.39858
16	H	4.05001	3.27928	1.63753
17	H	3.27195	5.96824	-1.66876
18	F	-2.37961	0.87364	-1.51555
19	F	-4.20879	2.23766	-0.06331
20	F	-3.44497	4.38357	1.39545
21	F	5.69595	6.82722	-1.77003
22	F	7.41001	5.77391	0.03730
23	F	6.53621	3.92259	1.80579

$Zn(2.4.6-C_6F_3H_2)_2$ CPCM toluene

Centre number	Atom type	Coordinates (Angstroms)		
		X	Y	Z
1	Zn	1.61597	3.95818	-0.05908
2	C	-2.60137	3.94109	0.30794
3	C	-2.91426	2.64520	-0.06071
4	C	-1.95987	1.71362	-0.42747
5	C	-0.64483	2.14504	-0.40988
6	C	-1.25790	4.27384	0.29284
7	C	-0.22706	3.41797	-0.05834
8	C	6.10777	5.39184	0.03216
9	C	5.79502	4.17279	0.60624
10	C	5.16559	6.20641	-0.56970
11	C	3.86369	5.73668	-0.57290
12	C	4.46493	3.79345	0.55160
13	C	3.44738	4.53392	-0.02644
14	F	-0.93405	5.54066	0.65183
15	F	0.30270	1.24298	-0.76611
16	F	4.14151	2.60119	1.11072
17	F	2.92733	6.52307	-1.15910
18	H	-2.22977	0.70583	-0.71178
19	F	-4.20730	2.27304	-0.06220
20	H	-3.36575	4.65150	0.59140
21	H	5.43445	7.15615	-1.01136
22	F	7.38822	5.80445	0.06043
23	H	6.54987	3.55346	1.07086

XYZ coordinates for the relaxed scan of the $ZnEt_2$ C-Zn-C bend angle.

$ZnEt_2$ CPCM Toluene (C-Zn-C bend = 180°)

Centre number	Atom type	Coordinates (Angstroms)		
		X	Y	Z
1	Zn	-0.18224	1.90834	-0.28164
2	C	-2.12781	1.69818	-0.17605
3	C	1.76334	2.11850	-0.38723
4	C	2.30542	3.35481	0.34845
5	H	2.05595	2.16156	-1.44275
6	H	2.24056	1.21587	0.01120
7	H	1.88194	4.27988	-0.05305
8	H	3.39519	3.44016	0.27220
9	H	2.05920	3.33021	1.41386
10	C	-2.66425	1.53023	1.25511
11	H	-2.42321	0.83446	-0.78272
12	H	-2.60635	2.56453	-0.64681
13	H	-2.23885	0.64878	1.74352
14	H	-3.75393	1.41813	1.28159
15	H	-2.41463	2.39082	1.88237

$ZnEt_2$ CPCM Toluene (C-Zn-C bend = 174°)

Centre number	Atom type	Coordinates (Angstroms)		
		X	Y	Z
1	Zn	-0.21642	1.96981	-0.40511
2	C	-2.14680	1.69065	-0.20840
3	C	1.73680	2.13185	-0.41637
4	C	2.27392	3.32937	0.38440
5	H	2.09860	2.18821	-1.44915
6	H	2.15298	1.20235	-0.01048
7	H	1.90894	4.27889	-0.01729
8	H	3.36867	3.37708	0.37846
9	H	1.96036	3.28714	1.43155
10	C	-2.59438	1.56131	1.25730
11	H	-2.42910	0.78615	-0.75928
12	H	-2.69497	2.50930	-0.68784
13	H	-2.09384	0.72848	1.75978
14	H	-3.67288	1.39086	1.34951
15	H	-2.36156	2.46303	1.83095

$ZnEt_2$ CPCM Toluene (C-Zn-C bend = 168°)

Centre number	Atom type	Coordinates (Angstroms)		
		X	Y	Z
1	Zn	-0.16951	1.78657	-0.43903
2	C	-2.11573	1.76240	-0.19795
3	C	1.77287	2.04859	-0.37037
4	C	2.15643	3.36757	0.32226
5	H	2.20148	2.02363	-1.37818
6	H	2.23063	1.20840	0.16406
7	H	1.74804	4.23443	-0.20521
8	H	3.24184	3.50686	0.37828
9	H	1.77416	3.41221	1.34634
10	C	-2.53349	1.50119	1.25887
11	H	-2.58083	1.01760	-0.85281
12	H	-2.51309	2.73008	-0.52536
13	H	-2.19146	0.52215	1.60668
14	H	-3.62107	1.52808	1.39010
15	H	-2.10996	2.24469	1.94034

$ZnEt_2$ CPCM Toluene (C-Zn-C bend = 162°)

Centre number	Atom type	Coordinates (Angstroms)		
		X	Y	Z
1	Zn	-0.16438	1.69145	-0.59783
2	C	-2.09762	1.67172	-0.25444
3	C	1.76028	2.05864	-0.47028
4	C	2.02471	3.26185	0.45129
5	H	2.19526	2.24905	-1.45713
6	H	2.28201	1.17683	-0.08234
7	H	1.54801	4.17098	0.07338
8	H	3.09371	3.47834	0.55542
9	H	1.63177	3.09320	1.45826
10	C	-2.40534	1.66720	1.25281
11	H	-2.58716	0.81677	-0.73238
12	H	-2.54059	2.56133	-0.71655
13	H	-2.01508	0.76952	1.74078
14	H	-3.48125	1.70267	1.45691
15	H	-1.95402	2.52490	1.76014

$ZnEt_2$ CPCM Toluene (C-Zn-C bend = 157°)

Centre number	Atom type	Coordinates (Angstroms)		
		X	Y	Z
1	Zn	-0.16410	1.57621	-0.74696
2	C	-2.06518	1.45840	-0.26354
3	C	1.70277	2.17857	-0.62154
4	C	1.91756	2.95694	0.68854
5	H	1.94880	2.82066	-1.47434
6	H	2.40551	1.34033	-0.67050
7	H	1.24454	3.81613	0.76437
8	H	2.94004	3.34010	0.77951
9	H	1.73027	2.33000	1.56508
10	C	-2.27635	2.04131	1.14550
11	H	-2.43804	0.42967	-0.29798
12	H	-2.66924	2.01738	-0.98650
13	H	-1.72425	1.47690	1.90249
14	H	-3.33074	2.03434	1.44342
15	H	-1.93129	3.07752	1.21049

$ZnEt_2$ CPCM Toluene (C-Zn-C bend = 151°)

Centre number	Atom type	Coordinates (Angstroms)		
		X	Y	Z
1	Zn	-0.18709	1.63729	-0.89666
2	C	-2.02241	1.38228	-0.22939
3	C	1.66226	2.27505	-0.67068
4	C	1.83701	2.76275	0.77886
5	H	1.88313	3.09320	-1.36424
6	H	2.39681	1.49315	-0.88872
7	H	1.13991	3.57050	1.02049
8	H	2.84763	3.14097	0.96965
9	H	1.65198	1.96052	1.49931
10	C	-2.20105	2.22975	1.04328
11	H	-2.19999	0.32697	0.00386
12	H	-2.78305	1.65636	-0.96707
13	H	-1.47306	1.95895	1.81372
14	H	-3.19694	2.11050	1.48447
15	H	-2.06482	3.29623	0.84116

$ZnEt_2$ CPCM Toluene (C-Zn-C bend = 145°)

Centre number	Atom type	Coordinates (Angstroms)		
		X	Y	Z
1	Zn	-0.20500	1.68189	-1.01475
2	C	-1.98908	1.38346	-0.22566
3	C	1.64461	2.28896	-0.68370
4	C	1.74040	2.72634	0.78878
5	H	1.92764	3.12162	-1.33558
6	H	2.37269	1.49540	-0.88064
7	H	1.05638	3.55026	1.01147
8	H	2.74865	3.06298	1.05468
9	H	1.48307	1.91023	1.47056
10	C	-2.12149	2.26348	1.02922
11	H	-2.08843	0.32859	0.05315
12	H	-2.81682	1.58896	-0.91124
13	H	-1.32965	2.05480	1.75413
14	H	-3.07758	2.10966	1.54194
15	H	-2.05506	3.32785	0.78567

$ZnEt_2$ CPCM Toluene (C-Zn-C bend = 140°)

Centre number	Atom type	Coordinates (Angstroms)		
		X	Y	Z
1	Zn	-0.22149	1.72453	-1.11319
2	C	-1.94174	1.37111	-0.20270
3	C	1.61960	2.32099	-0.69760
4	C	1.66439	2.65565	0.80371
5	H	1.92576	3.19515	-1.28056
6	H	2.35368	1.54044	-0.92285
7	H	0.98401	3.47367	1.05585
8	H	2.66669	2.95660	1.12844
9	H	1.37045	1.80005	1.41914
10	C	-2.06799	2.32447	0.99742
11	H	-1.93920	0.33356	0.14987
12	H	-2.82466	1.46522	-0.84223
13	H	-1.23670	2.20507	1.69692
14	H	-2.99206	2.15526	1.56167
15	H	-2.07043	3.37268	0.68414

XYZ coodinates for the relaxed scan of the $ZnEt_2$ C-C-Zn-C-C dihedral angle.

$ZnEt_2$ CPCM Hexane (C-C-Zn-C-C dihedral = 180°)

Centre number	Atom type	Coordinates (Angstroms)		
		X	Y	Z
1	Zn	0.09819	0.52460	0.04718
2	C	-1.76140	0.47486	0.65383
3	H	-1.90315	-0.42744	1.25524
4	H	-1.92127	1.30948	1.34216
5	C	-2.81480	0.51967	-0.46098
6	H	-2.71558	-0.32282	-1.14686
7	H	-3.83092	0.48898	-0.06129
8	H	-2.73395	1.42840	-1.05909
9	C	1.95777	0.57434	-0.55946
10	H	2.09952	1.47664	-1.16087
11	H	2.11764	-0.26027	-1.24779
12	C	3.01118	0.52953	0.55535
13	H	2.93033	-0.37920	1.15346
14	H	4.02729	0.56023	0.15566
15	H	2.91195	1.37202	1.24122

$ZnEt_2$ CPCM Hexane (C-C-Zn-C-C dihedral = 165°)

Centre number	Atom type	Coordinates (Angstroms)		
		X	Y	Z
1	Zn	0.10010	0.33314	0.03805
2	C	-1.76553	0.28757	0.62620
3	H	-1.98698	-0.71953	0.99038
4	H	-1.86733	0.93949	1.49831
5	C	-2.79696	0.68765	-0.43707
6	H	-2.75654	0.03226	-1.30821
7	H	-3.81751	0.64312	-0.05018
8	H	-2.63542	1.70498	-0.79624
9	C	1.96570	0.38109	-0.54999
10	H	2.05362	1.11504	-1.35593
11	H	2.20690	-0.58211	-1.00824
12	C	2.98996	0.69860	0.54752
13	H	2.96343	-0.03741	1.35228
14	H	4.01082	0.71144	0.15913
15	H	2.80853	1.67370	1.00174

$ZnEt_2$ CPCM Hexane (C-C-Zn-C-C dihedral = 150°)

Centre number	Atom type	Coordinates (Angstroms)		
		X	Y	Z
1	Zn	0.10010	0.33314	0.03805
2	C	-1.76553	0.28757	0.62620
3	H	-1.98698	-0.71953	0.99038
4	H	-1.86733	0.93949	1.49831
5	C	-2.79696	0.68765	-0.43707
6	H	-2.75654	0.03226	-1.30821
7	H	-3.81751	0.64312	-0.05018
8	H	-2.63542	1.70498	-0.79624
9	C	1.96570	0.38109	-0.54999
10	H	2.05362	1.11504	-1.35593
11	H	2.20690	-0.58211	-1.00824
12	C	2.98996	0.69860	0.54752
13	H	2.96343	-0.03741	1.35228
14	H	4.01082	0.71144	0.15913
15	H	2.80853	1.67370	1.00174

$ZnEt_2$ CPCM Toluene (C-C-Zn-C-C dihedral = 135°)

Centre number	Atom type	Coordinates (Angstroms)		
		X	Y	Z
1	Zn	0.10096	0.24170	0.03401
2	C	-1.77148	0.19857	0.60029
3	H	-2.03587	-0.83613	0.83573
4	H	-1.85753	0.74144	1.54571
5	C	-2.77476	0.76761	-0.41166
6	H	-2.75017	0.22363	-1.35693
7	H	-3.80049	0.71645	-0.03959
8	H	-2.56986	1.81421	-0.64121
9	C	1.97337	0.28801	-0.53212
10	H	2.04792	0.91797	-1.42293
11	H	2.25773	-0.71496	-0.86246
12	C	2.96625	0.78005	0.52925
13	H	2.95316	0.15018	1.41983
14	H	3.99244	0.78376	0.15496
15	H	2.74113	1.79658	0.85489

$ZnEt_2$ CPCM Toluene (C-C-Zn-C-C dihedral = 120°)

Centre number	Atom type	Coordinates (Angstroms)		
		X	Y	Z
1	Zn	0.10174	0.15521	0.03037
2	C	-1.77967	0.11453	0.56627
3	H	-2.08564	-0.93045	0.66802
4	H	-1.86042	0.53871	1.57101
5	C	-2.74534	0.84298	-0.37777
6	H	-2.72592	0.42008	-1.38316
7	H	-3.77797	0.78550	-0.02628
8	H	-2.49850	1.90133	-0.47275
9	C	1.98310	0.19959	-0.50538
10	H	2.05478	0.71486	-1.46731
11	H	2.30899	-0.82573	-0.70149
12	C	2.93546	0.85759	0.50181
13	H	2.92509	0.34469	1.46455
14	H	3.96868	0.85216	0.14741
15	H	2.66842	1.89798	0.69245

$ZnEt_2$ CPCM Toluene (C-C-Zn-C-C dihedral = 105°)

Centre number	Atom type	Coordinates (Angstroms)		
		X	Y	Z
1	Zn	0.10246	0.07508	0.02709
2	C	-1.78961	0.03687	0.52417
3	H	-2.13323	-1.00075	0.48900
4	H	-1.87592	0.33472	1.57287
5	C	-2.71053	0.91244	-0.33585
6	H	-2.68522	0.61826	-1.38602
7	H	-3.75142	0.84910	-0.01073
8	H	-2.42580	1.96465	-0.29303
9	C	1.99448	0.11737	-0.46984
10	H	2.07420	0.50929	-1.48767
11	H	2.35777	-0.91253	-0.52721
12	C	2.89936	0.92995	0.46570
13	H	2.88059	0.54271	1.48538
14	H	3.94099	0.91570	0.13707
15	H	2.59466	1.97618	0.51683

$ZnEt_2$ CPCM Toluene (C-C-Zn-C-C dihedral = 90°)

Centre number	Atom type	Coordinates (Angstroms)		
		X	Y	Z
1	Zn	0.10311	0.00243	0.02410
2	C	-1.80072	-0.03309	0.47417
3	H	-2.17585	-1.04561	0.30082
4	H	-1.90313	0.13301	1.55020
5	C	-2.67241	0.97491	-0.28639
6	H	-2.63057	0.81486	-1.36467
7	H	-3.72244	0.90667	0.00678
8	H	-2.35601	2.00297	-0.104 62
9	C	2.00689	0.04263	-0.42578
10	H	2.10525	0.30497	-1.48287
11	H	2.40115	-0.97401	-0.34220
12	C	2.86010	0.99576	0.42151
13	H	2.82217	0.74040	1.48142
14	H	3.91099	0.97343	0.12434
15	H	2.52427	2.02972	0.33095

Example input files:

Optimisation/Frequency calculation in ORCA 5.0.3

```
! wB97X-D3BJ ZORA-def2-TZVPP SARC/J RIJCOSX TightSCF SlowConv CPCM(toluene) ZORA defgrid3
! LargePrint
! Opt Freq

%SCF
	MaxIter 500
End

* xyzfile 0 1 opt.xyz
```

Zn 1s TDDFT calculation in ORCA 5.0.3

```
! wB97X-D3BJ ZORA-def2-QZVPP SARC/J RIJCOSX TightSCF CPCM(toluene) ZORA
! LargePrint

%tddft
	nroots 50
	maxdim 500
	OrbWin[0] = 0,0,-1,-1
	DoQuad True
	TDA True
end

* xyzfile 0 1 XAS.xyz
```

Zn XES KS-DFT calculation in ORCA 5.0.3

```
! wB97X-D3BJ ZORA-def2-QZVPP SARC/J RIJCOSX TightSCF CPCM(toluene) ZORA defgrid3
! LargePrint

%xes
CoreOrb 0
OrbOp 0
CoreOrbSoc 0
DOSOC True
DoQuad true
end

* xyzfile 0 1 XES.xyz
```

Relaxed scan (dihedral) calculation in ORCA 5.0.3

```
! wB97X-D3BJ ZORA-def2-TZVPP SARC/J RIJCOSX tightscf tightopt slowconv CPCM(Toluene) ZORA
! LargePrint
! Opt

%geom Scan
D 4 6 16 17 = 0, 180, 18
end
end

* xyzfile 0 1 opt.xyz
```

Relaxed scan (bend) calculation in ORCA 5.0.3

```
! wB97X-D3BJ def2-TZVPP def2/J RIJCOSX CPCM(toluene) TightSCF SlowConv
! LargePrint
! Opt

%geom Scan
A 1 0 2 = 180, 140, 8
end
end

* xyzfile 0 1 opt.xyz
```

Acknowledgements
KRJL acknowledges support from a Royal Society University Research Fellowship (URF\R\150353, URF\R\211005, RGF\EA\180089, RGF\R1\180053, RF\ERE\210061 and RF\ERE\231015). KRJL acknowledges support from an EPSRC Capital Award for Early Career Researchers. We acknowledge Diamond Light Source for time on Beamline I20 under Proposals SP24305, SP28565, SP30597, SP33163 and SP33520.

Supplementary references

1 Taft, R. W. in *Steric Effects in Organic Chemistry* (ed M. S. Newman) Ch. 13, (Wiley, 1956).

2 Markies, P. R. *et al.* COORDINATIONAL BEHAVIOR OF SOLVENT-FREE DIORGANYLZINC COMPOUNDS - THE REMARKABLE X-RAY STRUCTURE OF DIMERIC DIPHENYLZINC. *Organometallics* **9**, 2243-2247 (1990). https://doi.org:10.1021/om00158a022

3 Diaz-Moreno, S. *et al.* in *14th International Conference on X-Ray Absorption Fine Structure* Vol. 190 *Journal of Physics Conference Series* (eds A. DiCicco & A. Filipponi) (Iop Publishing Ltd, 2009).

4 Hayama, S. *et al.* Photon-in/photon-out spectroscopy at the I20-scanning beamline at diamond light source. *J. Phys.-Condes. Matter* **33**, 11 (2021). https://doi.org:10.1088/1361-648X/abfe93
